# Unravelling the fungal endomicrobiome of *Picrorhiza kurrooa* for increasing *in-planta* picroside biosynthesis using endophytic *Trichoderma harzianum* PKRF1

**DOI:** 10.1186/s40793-026-00909-4

**Published:** 2026-05-11

**Authors:** Anish Tamang, Anil Kumar, Ankita Thakur, Rakshak Kumar, Dinesh Kumar, Vipin Hallan, Shiv Shanker Pandey

**Affiliations:** 1 Biotechnology Division, CSIR-Institute of Himalayan Bioresource Technology (IHBT), Palampur, H.P 176061 India; 2 Fermentation and Phytofarming Technology Division, CSIR-Institute of Himalayan Bioresource Technology (IHBT), Palampur, H.P 176061 India; 3 Chemical Technology Division, CSIR-Institute of Himalayan Bioresource Technology (IHBT), Palampur, H.P 176061 India; 4 Academy of Scientific and Innovative Research (AcSIR), Ghaziabad, 201002 India; 5 Present Address: Department of Molecular Biology and Bioinformatics, Tripura University (Central University), Agartala, Tripura India

**Keywords:** Endophytic fungi, *Trichoderma harzianum*, *Picrorhiza kurrooa*, Next-generation sequencing, Secondary metabolites, Picrosides, Plant-microbe interaction, Culture-based validation, Microbiome restoration

## Abstract

**Background:**

Endophytic fungi form an integral part of plant microbiomes, influencing host physiology, stress resilience, and secondary metabolism. While next-generation sequencing (NGS) has greatly advanced the identification of endophytes, it often falls short of assigning functional roles, necessitating integration with culture-based approaches for downstream applications. *Picrorhiza kurrooa*, a critically endangered Himalayan medicinal herb valued for its hepatoprotective picrosides, suffers from reduced metabolite content in tissue culture-derived plants, likely due to microbiome loss in the course of aseptic in-vitro practices. Moreover, the diversity and functional role of fungal endomicrobiome in *P. kurrooa* remain unexplored.

**Methods:**

Internal transcribed spacer (ITS)-based amplicon sequencing was performed to assess and compare the endophytic fungal communities of wild-type (Wt) and in-vitro propagated (Tc) *P. kurrooa*. Fungal taxa unique to Wt-plants were identified and cross-referenced with culturable isolates. A dominant isolate present only in Wt-plants, *Trichoderma harzianum* PKRF1, was reintroduced into Tc-plants to evaluate its effect on plant growth and picroside biosynthesis. Whole-genome sequencing and comparative genomics of PKRF1 were also conducted to elucidate its functional capabilities and possible candidates for its endophytic nature.

**Results:**

Metagenomic analysis revealed a significant reduction in fungal diversity in Tc plants, with several taxa, including *Trichoderma*, *Cyphellophora*, and *Preussia*, exclusively associated with Wt-plants. Inoculation of Tc-plants with PKRF1 led to successful root colonization, enhanced photosynthetic efficiency, biomass, and significantly higher levels of picrosides. Transcript profiling confirmed upregulation of key biosynthetic genes. Genomic analysis of PKRF1 revealed genes associated with multiple plant-beneficial traits, including nutrient acquisition, phytohormone production, stress tolerance, plant colonization, and competitive interactions, distinguishing it from non-endophytic *Trichoderma* isolates.

**Conclusions:**

These findings provide the first comprehensive insight into changes in endophytic fungal diversity of *P. kurrooa* associated with in-vitro cultivation. Furthermore, the application of cultivated endophytes from wild plants demonstrated the potential to restore microbial functions lost during in-vitro propagation and enhance secondary metabolite production in cultivated plants. Overall, this approach offers a promising strategy to integrate metagenomic information into beneficial plant–microbe interactions for practical applications.

**Supplementary Information:**

The online version contains supplementary material available at 10.1186/s40793-026-00909-4.

## Introduction

Plants in their natural environment exist in close association with diverse microbial communities that play pivotal roles in their growth, development, and adaptation to stress. Among these microbes, endophytes are organisms that live inside plant tissues without causing apparent harm. They have garnered significant attention due to their ability to enhance plant fitness, improve stress tolerance, and modulate secondary metabolism [1]. The emergence of next-generation sequencing (NGS) has significantly advanced our understanding of microbial communities by facilitating high-throughput, culture-independent analyses. Techniques like ITS and 16 S rRNA sequencing uncover vast taxonomic diversity, including rare and unculturable taxa, offering insights into microbial roles in stress tolerance and metabolite synthesis [2, 3]. NGS has streamlined the identification of key endophytes in medicinal plants through high-throughput computational screening. However, its ability to assign definitive functional roles to detected taxa remains limited [4]. NGS data alone are insufficient for downstream applications, as the organisms identified cannot be directly exploited without phenotypic characterization. This underscores the necessity of culture-based techniques, which enable the isolation, maintenance, and functional validation of specific microbial strains. Axenic culturing facilitates the development of endophytes as biofertilizers or biocontrol agents, supporting their application in sustainable agriculture through targeted inoculation [5]. Thus, the integration of NGS with culture-dependent approaches bridges the gap between microbial discovery and functional deployment.

Fungal endophytes are known to establish long-term, systemic colonization within host plants, often influencing physiological processes such as photosynthesis, nutrient uptake, and defense responses [5, 6]. Unlike bacterial endophytes, fungi possess extensive hyphal networks that enable deeper tissue penetration and more stable colonization, making them particularly effective in promoting plant growth and stress resilience [7]. Furthermore, fungal endophytes have been shown to enhance the biosynthesis of pharmaceutically valuable secondary metabolites in medicinal plants [8], highlighting their potential in biotechnological applications. However, modern agricultural practices, including domestication, intensive breeding, and the overuse of chemical inputs, have led to a substantial decline in these beneficial microbial populations, potentially compromising plant health and productivity [9]. Reintroducing lost microbial symbionts could offer a sustainable strategy to restore plant-microbe interactions and improve crop performance. *Picrorhiza kurrooa*, a critically endangered Himalayan medicinal herb, epitomizes this challenge and is valued for its hepatoprotective picrosides I and II; however, overharvesting and habitat loss have driven wild populations to near extinction. While in vitro propagation aids conservation, micropropagated plants exhibit reduced picroside levels, likely due to the loss of microbial symbionts during cultivation. Our previous investigation revealed that there is a loss of bacterial endophytes during in vitro propagation, and this reduced diversity correlated with diminished picroside biosynthesis [10]. These observations suggested a potential association between reduced microbial diversity and picroside content in in-vitro plants, highlighting the possible benefit of restoring lost endophytes. However, the relative contributions and functional roles of bacterial and fungal endophytes in picroside biosynthesis, particularly the fungal component in *P. kurrooa*, remain insufficiently understood.

To address the gap, the present study focuses on plant-associated fungal communities and their functional roles by integrating next-generation sequencing (NGS) and culture-based approaches.ITS-based amplicon sequencing of wild-type (Wt) and in vitro propagated (Tc) *P. kurrooa* was conducted to identify key fungal taxa associated with the native host, revealing several taxa unique to Wt plants. A distinctive endophytic fungus, PKRF1, was isolated from Wt plants and reintroduced into Tc plants to assess its influence on plant growth and picroside accumulation. Whole-genome sequencing, including comparative genomics of PKRF1, was also performed to explore its genetic potential in mediating plant-microbe interactions and endophytic potential. This represents the first comprehensive analysis of fungal endophytes in *P. kurrooa* and highlights the role of microbial restoration in enhancing secondary metabolite production in cultivated plants, offering a novel strategy for the conservation and sustainable utilization of this endangered medicinal species.

## Materials and methods

### Plant material, DNA extraction, and ITS amplicon library preparation

As described in our previous study [10], wild-type (Wt) *P. kurrooa* plants were collected from Rohtang Pass, Himachal Pradesh, India (N 32° 22′ 27.12″, E 77° 15′ 21.48″; 3992 masl). Whole plants, including intact roots and rhizomes with adhering soil, were carefully excavated using sterile tools, taxonomically verified at our institute, and deposited under voucher number PLP16488. Tissue culture-derived (Tc) plants were generated in-vitro from wild explants, and only those with well-developed leaves, roots, and rhizomes were selected for further analyses. For both Wt and Tc groups, composite samples were prepared by pooling 10 plants per replicate, with three biological replicates processed independently (*n* = 3).

Total DNA was extracted from surface-sterilized tissues following the protocol detailed in our previous publication [10]. Libraries were constructed using the QIAseq 16 S/ITS Region Panels (Qiagen, Hilden, Germany). Fungal ITS regions were amplified using proprietary phased ITS primers included in the QIAseq 16 S/ITS Region Panel (Qiagen, Hilden, Germany), which targets fungal ITS regions as part of a multiplex primer pool according to the manufacturer’s instructions. Sterile water was included as a negative control during library preparation, and no amplification was detected; thus, negative controls were excluded from sequencing. Library quality and quantity were assessed using a Bioanalyzer (Agilent Technologies, Santa Clara, CA, USA), and sequencing was performed on an Illumina MiSeq platform.

The QIAseq 16 S/ITS panels simultaneously amplify bacterial 16 S rRNA and fungal ITS regions, generating a dataset that can be used to investigate both bacterial and fungal communities. In our previous publication, only the 16 S rRNA reads from this dataset were analyzed to characterize bacterial endophytes. In contrast, the present study exclusively utilizes the ITS-derived reads to profile fungal endophyte diversity. Although the plant material, DNA preparation, and sequencing pipeline are identical to our earlier work, the fungal diversity explored here represents an entirely novel aspect of the *P. kurrooa* microbiome.

### Diversity analysis based on ITS-based amplicon sequencing

The reads were first demultiplexed and trimmed, following which quality was checked through FastQC v0.119. Cutadapt v3.4 was used to remove low-quality reads having a Phred score less than 30 [11]. Microbial diversity and composition were analyzed using the QIIME2 v2022.2 pipeline [12], where DADA2 was employed for denoising and removal of chimeric sequences [13]. Amplicon sequence variants (ASVs) were generated and taxonomically assigned using theUNITE ITS reference database (release 9.0) [14]. The resulting ASV table, along with taxonomic annotations and sample metadata, was uploaded to the MicrobiomeAnalyst R server for further analysis [15]. Features detected in fewer than 10% of samples were excluded, and the data were rarefied to the minimum library size of 21,331 reads per sample to normalize sequencing depth prior to diversity analysis. Alpha diversity indices including Observed species richness, Shannon index, and Simpson index were calculated, while beta diversity was assessed using Bray–Curtis dissimilarity and visualized through principal coordinate analysis (PCoA). Differentially abundant taxa between wild-type (Wt) and tissue culture–derived (Tc) plants were identified using Linear Discriminant Analysis Effect Size (LEfSe, v1.0) with a linear discriminant analysis (LDA) score cutoff of ≥ 2. The Kruskal–Wallis test was applied to detect significantly enriched taxa, followed by LDA to estimate effect sizes [16]. To check the presence of metabolite pathways present in the fungal communities, functional prediction was done using PICRUSt2 (Phylogenetic investigation of communities by reconstruction of unobserved states) [17] and the plots were generated using Microbiome Analyst R server.

### Isolation of endophytes

Endophytes were isolated from surface-sterilized leaves, roots, and rhizomes of the wild plants using the same biological replicates per tissue as described for ITS amplicon sequencing, following the protocol reported in our previous publication [18]. The tissues were first washed under running tap water, then surface sterilized by immersion in 1% sodium hypochlorite for 10 min, followed by 4–5 rinses with sterile 0.02 M potassium phosphate buffer (pH 7.0). To confirm sterility, 100 µl of the aliquot obtained from the final wash was inoculated into 5 ml of potato dextrose broth and incubated in a shaker (28 °C, 200 rpm) for a duration of 10 days. The surface-sterilized tissues were then cut into small pieces using sterile surgical blades and kept on potato dextrose agar (PDA) plates. After incubating the plates at 28 °C for 7 days, fungal colonies were subcultured to isolate pure cultures.

### Amplification of fungal ITS fragment, sequencing, and phylogenetic analysis

The genomic DNA of the isolated endophytes was extracted using the CTAB method [19]. The primers ITS1(5′-TCCGTAGGTGAACCTGCGG-3′) and ITS4 (5′-TCCTCCG CTTATTGATATGC-3′) were used for the amplification of the ITS region and were taken from earlier publications [18]. The PCR reaction (25 µl) was set up with genomic DNA (100 ng), forward and reverse primer (1 ul each from 10 µM stock), deoxyribonucleotide triphosphate mixture (0.5 µl from 10 mM stock), 10X PCR buffer, 0.2 ul Taq DNA polymerase (5 U µl^−1^) (Sigma-Aldrich Inc., St. Louis, Missouri, USA), following which volume was made up with MilliQ water. PCR amplification was performed in a SimpliAmp™ Thermal Cycler (Applied Biosystems, USA) under the following conditions: initial denaturation at 94 °C (45 s), annealing at 57.4 °C (45 s), extension at 72 °C (2 min), and a final extension at 72 °C (5 min). The PCR amplicons were electrophoresed on a 0.8% agaroe gel for visualization, then purified via ExoSAP-IT treatment (Applied Biosystems, USA) following the manufacturer’s protocol prior to sequencing. Following this, the purified products were subjected to sequencing with Big Dye Terminator cycle sequencing kit v.3.1 (Applied Biosystems, Waltham, Massachusetts, USA). The sequencing was performed with about 5 µL reaction mixture having approximately 50 ng template DNA and 1 pmol of sequencing primers. The cleanup of the reaction product was done using the Montage SEQ96 Sequencing Reaction Cleanup Kit (Millipore, Burlington, Massachusetts, USA) using a vacuum pump assembly. Following this, sequencing was done on Genetic Analyzer ABI 3130XL (Applied Biosystems, Waltham, Massachusetts, USA). The similarity of nucleotide sequence was determined using the NCBI BLAST search. The partial sequence of the ITS fragment was deposited in the GenBank (NCBI). The nucleotide sequences were aligned using the MUSCLE algorithm in MEGA X to determine phylogenetic relationships among the fungal isolates. The resulting alignments were then employed to construct phylogenetic trees via Interactive Tree of Life (iTOL) v5 (https://itol.embl.de/) [20].

### In vitro screening for plant growth–promoting traits

Endophytic isolates were evaluated for plant growth–promoting traits, including phosphate solubilization, indole acetic acid (IAA) production, and siderophore production. All assays were performed in triplicate.

#### Phosphate solubilization

Quantitative phosphate solubilization was assessed in NBRIP broth containing 0.5% (w/v) tricalcium phosphate (TCP) [21]. Isolates were pre-cultured on PDA for 5 days, inoculated into NBRIP broth, and incubated at 28 °C with shaking (200 rpm) for 5 days. The cell-free supernatant was collected and soluble phosphorus was quantified by measuring absorbance at 882 nm using a Synergy H1 plate reader, with KH_2_PO_4_ used to generate a standard curve. The pH of each culture supernatant was also recorded [22].

#### Indole acetic acid (IAA) production

The quantification of IAA production was done by incubating 5 mm fungal discs in potato dextrose broth supplemented with 0.5 mg/ml L-tryptophan and incubated at 28 °C with shaking (200 rpm) for 5 days. After centrifugation, 100 µl of culture supernatant was mixed with 100 µl of Salkowski reagent (2 ml 0.5 M FeCl_3_ in 98 ml 35% HClO_4_) in 96-well plates, incubated in the dark for 20 min, and absorbance was measured at 530 nm. IAA concentration (µg/ml) was calculated using an IAA standard curve [23].

#### Siderophore production

The quantification of the siderophore production was done by inoculating the isolates in iron-free Czapek–Dox broth [24] and incubated at 28 °C with shaking (200 rpm) for 5 days. After centrifugation (6000 × g, 10 min), 100 µl of the supernatant was mixed with 100 µl of CAS dye solution, incubated in the dark for 15 min, and the absorbance was measured at 630 nm. Siderophore production was expressed as siderophore units (SU, %) calculated as:

$$ \% SU = \frac{{(Ar - As)}}{{Ar}} \times 100 $$

where *Ar* is the absorbance of the control and *As* is the absorbance of the sample [23].

### Treatment of in vitro propagated *P. kurrooa* plant with PKRF1

 In-vitro propagated plantlets, acclimatized under greenhouse conditions and bearing 4–5 fully expanded leaves, were utilized to investigate the effect of the treatment of isolated endophyte. For each condition, 40 plants were used for the PKRF1 treatment, and 40 plants served as mock-treated controls. The inoculation of the endophytic fungi, following the general approach described, in earlier publications [18], with some modification. Spores of PKRF1 were produced in liquid culture by inoculating the fungal isolate into sterile potato dextrose broth (PDB) and incubating at 28 ± 2 °C under shaking conditions (120–150 rpm) for 5–7 days until sufficient sporulation was achieved. The resulting culture was filtered through sterile muslin cloth to separate mycelial fragments, and the spore suspension was collected. The concentration of spores was determined using a hemocytometer and adjusted to the required concentration using sterile 0.8% NaCl solution prior to inoculation. Plant roots were immersed for 3 h in an endophyte spore suspension (1 × 10^8^ spores/conidia mL^−1^) prepared in 0.8% NaCl solution. After treatment, the plants were transferred to sterilized pots (17 cm height × 22 cm top diameter × 12 cm bottom diameter; 3.7 L capacity) containing an autoclaved soil mixture (soil: sand: vermicompost, 1:1:1 ratio). The plants were grown in a glasshouse maintained at 18 °C with a 16-h photoperiod and 350 µmol m^−2^ s^−1^ photosynthetic photon flux density, and irrigated with sterile water as needed. The plants treated with only saline (0.8%) solution were treated as controls. The plants were given a booster dose after 15 days with 10 mL pot^−1^ of fungal suspension culture, which was applied as a soil drench around the root zone. Further, to minimize the variation in the plant development stage and other variables, sampling for all the analyses was carried out during the same stage, neither too young nor too old, and at the position of the leaves (third leaf from the top). Control plants were treated exclusively with 0.8% slt solution. Further, to minimize the variation in the plant development stage and other variables, sampling for all the analyses was carried out at 180 days after inoculation, using leaves collected from a consistent position (third leaf from the top).

### Confirmation of colonization by PKRF1

The confirmation of colonization of PKRF1 was done through confocal microscopy on six randomly selected plants from both the treatment and mock control groups. Root tissues were cut into segments and immersed in 10% KOH (overnight at 4 °C). The samples were further washed with Phosphate-buffered saline (PBS) buffer (8 mM Na_2_HPO_4,_ 2.7 mM KCL, 1.5 mM KH_2_PO_4_, 137 mM NaCl) (pH 7.4) 4–5 times for neutralization. For visualization of the endophytic colonization in the root tissue samples, Wheat Germ Agglutinin Alexa Fluor 488 (WGA-AF488) and Propidium iodide (PI) dyes were used. The tissue was treated with a staining solution (10 µg/mL WGA-AF488, 20 µg/mL PI, 0.1% Tween 20 in PBS buffer), and the samples were subjected to vacuum infiltration (250 millibars for 5 min), repeated for 3–4 times with 5 min time interval between each cycle. After the infiltration, the samples were washed twice in PBS (pH 7.4), following which the imaging was done through confocal microscopy [25].

### Measurement of plant biomass

To estimate the effect of PKRF1 treatment on growth promotion, the biomass of the above-ground (leaves) and below-ground (roots) parts of the plants was determined. For each treatment and mock control, plants were analyzed as five independent sets, each consisting of pooled samples from eight plants. The procedure followed was according to our previous publication [26]. The plants were gently harvested and cleaned to remove soil and dirt. Plant biomass was evaluated by separating above and below-ground plant parts, recording fresh weights, and subsequently drying the tissues at 70 °C for five days to determine dry mass.

### Determination of photosynthetic pigments

Photosynthetic pigment analysis was performed on fully expanded leaves (third leaf from the apex) of both PKRF1 and mock-treated *P. kurrooa* plants. For each treatment and mock control, five independent biological sets were analyzed, each consisting of pooled leaf samples from eight plants, and each biological sample was measured using three technical replicates. Chlorophyll *a* (Chl *a*), chlorophyll *b* (Chl *b*), total chlorophyll (Chl *a* + *b*), Chl *a*/*b* ratio, and carotenoid content. Fresh leaf tissue (100 mg) was homogenized in 10 ml of ice-cold 100% methanol and incubated at 4 °C until complete tissue decolorization. The extract was centrifuged (4000 × g, 5 min) to obtain a clear supernatant, which was then analyzed for pigment concentrations according to the previously described method [27].

### Chlorophyll fluorescence imaging

Chlorophyll fluorescence imaging was performed using a FluorCAM FC 800-C system (Photon Systems Instruments, Czech Republic) following a 20-m dark adaptation period. Five plants were randomly selected from both the treatment and mock controls, and each plant sample was analyzed individually, with three technical replicates per sample. Key photosynthetic parameters were quantified, including maximum PSII photochemical efficiency (Qy_max), PSII quantum yield (ΦPSII), non-photochemical quenching (NPQ), photochemical quenching parameters (qP and qL), non-photochemical quenching coefficient (qN), and relative fluorescence decrease ratio (Rfd) under steady-state conditions. Fluorescence images were acquired and analyzed using FluorCam7 software, with results visualized as pseudo-color images representing fluorescence intensity gradients from low (blue) to high (red). All measurements were conducted on fully dark-adapted leaves to ensure accurate assessment of photosynthetic performance [28].

### Determination of starch

The starch content was quantified using pooled leaf samples from plants of each treatment and mock control, prepared as five independent sets, each consisting of leaves collected from eight plants. The same sampling strategy was followed for all subsequent analyses described in Sects. “Measurement of hydrogen peroxide”, “Measurement of malondialdehyde”, “Secondary metabolite profiling” and “Expression analysis of key secondary metabolite biosynthesis genes”, unless otherwise stated. Briefly, 100 mg of pooled leaf tissues was homogenized in liquid nitrogenfollowed by sequential washes with hot 80% ethanol and acetone to remove soluble sugars and pigments until the extract became colorless. Starch was then extracted using 35% perchloric acid and quantified colorimetrically using anthrone reagent (prepared with 1.146 g anthrone powder, 500 mL concentrated sulfuric acid, and 200 mL water). After boiling the samples for 12 min, they were immediately cooled on ice, and starch concentration was determined by measuring absorbance at 625 nm [29].

### Measurement of hydrogen peroxide

The hydrogen peroxide content was measured by homogenizing 100 mg of leaf tissue in liquid nitrogen and extracting with 1 mL of 0.1% (w/v) trichloroacetic acid. After centrifugation (1200 × g, 15 min, 4 °C), the supernatant was mixed with 0.4 ml of 10 mM potassium phosphate buffer (pH 7.0) and 0.8 ml of 1 M potassium iodide, followed by dark incubation for 20 min. Absorbance was measured at 390 nm against an H_2_O_2_ standard curve prepared with known concentrations [30].

### Measurement of malondialdehyde

Lipid peroxidation was assessed by quantifying malondialdehyde (MDA) content using the thiobarbituric acid (TBA) method [31].Fresh leaf tissue (200 mg) was homogenized in liquid nitrogen and extracted with 2 ml of TBA reaction solution [containing 0.5% TA, 20% tichloroacetic acid, and 0.25 ml of 175 mM NaCl in 50 mM Tris-Cl buffer (pH 8.0)]. The mixture was heated at 95 °C for 5 min, rapidly cooled on ice, and centrifuged (14,000 × g, 5 min, 4 °C). The absorbance of the supernatant was measured at 450, 532, and 600 nm, and MDA concentration was calculated using the standard formula:

$$ \begin{aligned} & {\text{MDA }}(\mu {\mathrm{mol}}\;{\mathrm{g}}^{{ - 1}} {\mathrm{FW}}) = {\mathrm{6}}.{\mathrm{45}} \times \\ & \left( {{\mathrm{OD}}_{{{\mathrm{532}}}} - {\mathrm{OD}}_{{{\mathrm{6}}00}} } \right) \\ & - \left( {0.{\text{56 }} \times {\text{ OD}}_{{{\mathrm{45}}0}} } \right) \\ \end{aligned} $$

### Secondary metabolite profiling

Secondary metabolite profiling was conducted from leaf and root tissues of both treated and control plants Dried samples were finely ground, and 100 mg aliquots were extracted in 5 mL HPLC-grade methanol for 24 h. The samples were further sonicated (35 °C, 30 min) and centrifugation (1,000 rpm, 2 min). The clarified supernatant was filtered (0.22 μm) and analyzed by UHPLC-PDA (Shimadzu LC-MS 2020) using a Waters BEH C18 column (2.1 × 100 mm, 1.7 μm particle size) with PDA detection. The mobile phase consisted of 0.1% formic acid in water (Solvent A) and 0.1% formic acid in acetonitrile (Solvent B) at a flow rate of 0.28 ml/min, with the following gradient program: 0 min (95:5), 9 min (65:35), 11 min (60:40), 12 min (10:90), 16 min (10:90), 16.5 min (95:5), and 20 min (95:5) (A: B ratio). Target compounds (P-I, P-II, P-III, caffeic acid, cinnamic acid, vanillic acid, catalpol, and aucubin) were quantified according to established methods [32]. Statistical significance was determined using one-way ANOVA with Duncan’s post hoc test (*p* ≤ 0.05).

### Expression analysis of key secondary metabolite biosynthesis genes

Total RNA was isolated from leaves and root tissues of PKRF1-treated and control plants using TRIzol reagent (ThermoFisher Scientific, Waltham, Massachusetts, USA), followed by DNase treatment. cDNA was synthesized with 2 µg of total RNA using a Verso cDNA synthesis kit (ThermoFisher Scientific, Waltham, Massachusetts, USA). Relative expression analysis of key genes of picroside biosynthesis pathways was analyzed for PKRF1-treated and mock-inoculated plants. Quantitative PCR was performed in 10 µl reactions containing: 5 µl SYBR Green master mix (Thermo Fisher Scientific, USA), 300 nM of each primer, and 1 µl of 1:10 diluted cDNA template. Thermal cycling conditions consisted of an initial denaturation at 95 °C for 10 min, followed by 40 cycles of 95 °C for 15 s (denaturation) and 60 °C for 1 min (annealing/extension). Gene expression levels were quantified using the comparative Ct method, with normalization to the endogenous 26 S rRNA reference gene of *P. kurrooa*. The expression analysis of key picroside biosynthesis genes was carried out encompassing the phenylpropanoid, mevalonate and non-mevalonate pathways viz. *DAHPS*, *DXS*, *DXR*, *G10H*, *PAL*, *C4H* and *CAM*. The primer sequences used for quantitative real-time PCR (qRT-PCR) analysis are listed in Supplementary Table S11.

### Whole genome sequencing: DNA isolation, library preparation and sequencing

The genomic DNA of PKRF1 was isolated using cetyltrimethylammonium bromide extraction buffer (CTAB, 100 mM Tris–HCl, 1.4 M NaCl, 20 mM EDTA, 2-mercaptoethanol, pH 8.0) as detailed in Sambrook and Russell [19]. Further, the isolated DNA was processed for library preparation, and the quality of the prepared library was analyzed through Bioanalyzer (Agilent Technologies, Santa Clara, CA, USA). The prepared library was sequenced on an Illumina NovaSeq 6000 platform (Illumina, San Diego, USA) using paired-end sequencing with a read length of 2 × 150 bp and NovaSeq 6000 S4 reagent kit v1.5.

### Genome assembly and annotation

The raw reads obtained after sequencing were evaluated and assembled using a previously described method [33]. In detail, the raw reads were checked for quality using the FastQC toolkit, followed by quality trimming using the cutadapt tool. The good-quality Illumina reads were assembled using the Unicycler assembler [34]. The assembly quality was checked using BUSCO ascomycota_odb10 dataset [35] and QUAST tools [36]. Further, the assembled genome was annotated using the Funannotate tool (https://zenodo.org/record/4054262#.YhxHjOpBxPY) using a seed species of *Verticillium longisporum*. In detail, the assembled contigs were cleaned and sorted before the annotation, and further, the repetitive elements were soft-masked from the assembly. The genes and tRNAs in the assembly were predicted using AUGUSTUS [37], GlimmerHMM [38], and tRNAscan-SE [39] employed in Funannotate. The predicted genes were annotated using MEROPS [40], UniProtKB (UniProt Consortium), Pfam [41], GO ontology [42], and InterProScan databases [43]. The PKRF1 assembly was also annotated using COG [44] and the KEGG database [45] to evaluate the functional pathways.

### Phylogenomic and comparative genomics for PGP and secondary metabolites synthesis

The phylogenomics tree of the strain PKRF1 was constructed using the UFCG tool [46]. In detail, the genomic sequences of strains for the *Trichoderma* genus were downloaded from NCBI. The single-copy core genes from all the fungal strains were extracted and aligned from genome assemblies, and further, a maximum likelihood tree was constructed using the UBCG tool. Average nucleotide identity (ANI) values between the PKRF1 strain and other *Trichoderma* species were evaluated by the FastANI tool [47], and results were plotted using the ANIclustermap tool. Further, non-endophytic *Trichoderma* strains were searched and downloaded from NCBI, and the PKRF1 genome was compared with non-endophytic strains using the OrthoVenn3 tool [48]. The gene family contraction and expansion analysis were done using the CAFE 5 tool [49] employed in OrthoVenn3. The expansion or contraction of genes happens during evolution, have the same structure or function, and contribute to species differentiation [50]. The annotated genes present in strain PKRF1, compared to the other non-endophytic strains, were searched for their function in plant growth promotion using the PLaBAse server [51]. The complete genome of PKRF1 was also annotated using the PLaBAse server to evaluate the overall plant growth promotion attributes of the fungal isolate. The secondary metabolite gene clusters present in the PKRF1 strain and non-endophytic *Trichoderma* strains were evaluated by using the Antismash server [52] and compared with the Multismash tool [53].

## Results

### Complete fungal diversity based on ITS-based amplicon sequencing

**Fig. 1:**
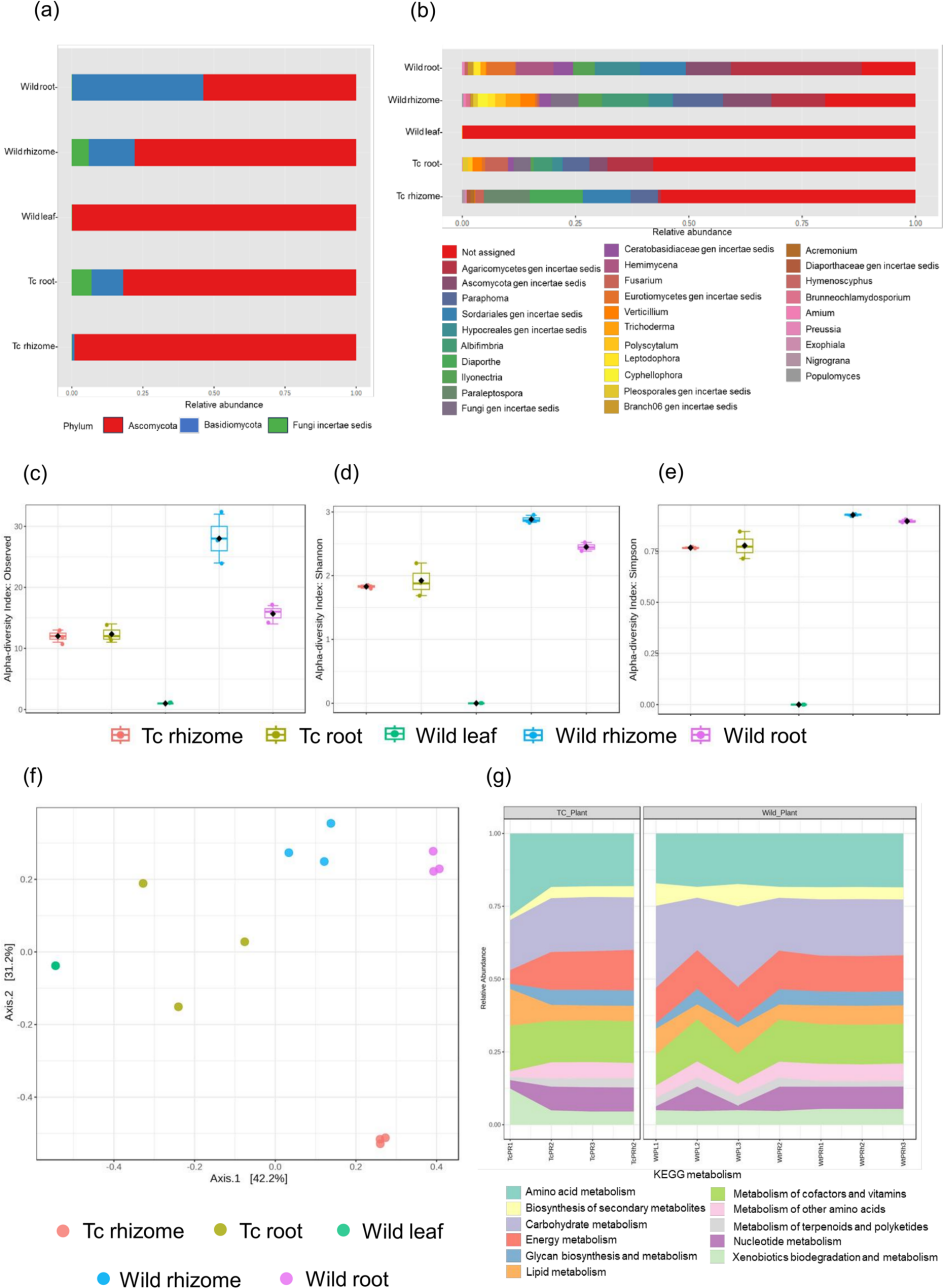
Relative abundance of fungal taxa in different anatomical parts of Wt and Tc *Picrorhiza kurrooa* at **a** Phylum and **b** genus level; Alpha diversity based on **c** observed number of species, **d** Shannon, and **e** Simpson indexes. The Y-axes indicate the values for the corresponding index, and the X-axes indicate the different parts anatomical parts of Wild and Tc plants of *P. kurrooa;* **f** Beta diversity analysis, two-dimensional scatter plots were generated using a PCoA based on the unweighted UniFrac distance metric Bray Curtis. Samples corresponding to different parts of Wild (Wt) and Tc plants of *P. kurrooa* were plotted as coloured dots; **g** Pathway abundance in the microbial community, Fungal endophytes in Wild and TC (tissue cultures) plants. “‘Not assigned’ indicates sequences that could not be classified to any fungal taxonomic group at the defined confidence threshold, whereas ‘Fungi_gen_incertae_sedis’ represents sequences assigned to the fungal kingdom but with unresolved genus-level classification

To understand the fungal endophytic diversity composition of wild (Wt) and tissue-cultured (Tc) plants, amplicon-based sequencing was carried out. The fungal endophytic taxa at the phylum level mostly belonged to Ascomycota and Basidiomycota, and the remaining belonged to unclassified fungi (Fungi_phy_Incertae_sedis) (Fig. 1a and S1a, Table S1). In Wt plants Ascomycota (71.64%), Basidiomycota (24.25%), and Unclassified fungi (4.12%) were observed, whereas Tc plants were predominantly composed of Ascomycota (94.94%), Basidiomycota (4.03%), and Unclassified fungi (1.04%). At the tissue level, in Wt plants, the rhizome harbored 77.55% Ascomycota, 16.45% Basidiomycota, and 6.01% unclassified fungi; the root tissues harbored 58.51% Ascomycota and 41.49% Basidiomycota, whereas the leaf tissues harbored 100% Ascomycota. Similarly, for the Tc plants, the rhizome tissues harbored 99.12% Ascomycota and 0.88% Basidiomycota; root tissues harbored 84.77% Ascomycota, 11.68% Basidiomycota, and 3.55% unclassified fungi (Table S1). Notably, Tc leaf tissues were also analyzed; however, no detectable endophytic fungal taxa were identified in these samples.

Further, the taxa identified at the genus level included the *Agaricomycetes* [Wt (17.33%), Tc (3.63%)], *Albifimbria* [Wt (7.08%), Tc (1.23%)], *Ilyonectria* [Wt (5.01%) and Tc (0.21%)], *Paraphoma* [Wt (7.53%), Tc (6.00%)], *Polyscytalum* [Wt (1.63%) and Tc (0.32%)], *Verticillium* [Wt (2.19%), Tc (0.57%)], *Fusarium* [Wt (0.43%) and Tc (2.93%)], present in both Wt and Tc *P. kurrooa* plants. Some taxa not identified at the genus level were depicted in the next higher taxa level, such as *Ceratobasidiaceae* [Wt (3.21%), Tc (0.40%)], *Eurotiomycetes* [Wt (2.34%), Tc (0.22%)], *Hypocreales* [Wt (6.75%), Tc (0.62%)], *Pleosporales* [Wt (0.55%), Tc (0.37%)], and *Sordariales* [Wt (3.09%), Tc (7.34%)]. Some of the distinct taxa only present on Wt included *Trichoderma* (2.51% %), *Hemimycena* (2.51%), *Cyphellophora* (1.61%), *Leptodophora* (1.61%), *Brunneochlamydosporium* (0.51%), *Arnium* (0.49%), *Hymenoscyphus* (0.24%), *Populomyces* (0.16%), and *Preussia* (0.15%) (Fig. 1b; Fig. S1b and Table S2).

In Wt plants, at the genus level the most dominant taxa in the rhizome tissues were *Paraphoma* (10.99%), followed by *Albifimbria* (10.32%), *Ilyonectria* (5.18%), *Trichoderma* (3.11%), *Verticillium* (3.19%) and other less abundant taxa. whereas in the root tissues the most abundant identified taxa included *Agaricomycetes* (29.02%), *Hemimycena* (8.01%), *Ilyonectria* (4.68%), *Trichoderma* (1.22%). In contrast, in the leaf tissues, most taxa were under the unclassified fungi. The Tc plants tissue-specific taxa included *Fusarium* (5.06%), *Paraphoma* (5.87%), and *Verticillium* (1.94%) in the roots, whereas the abundant taxa in rhizome were *Diaporthe* (11.94%). Among the distinct taxa present in Wt plants, most were associated with rhizome tissues (*Arnium*, *Brunneochlamydosporium*, *Cyphellophora*, *Populomyces*), whereas root tissues harbored *Hemimycena*, *Hymenoscyphus*, and *Preussia*. Whereas *Trichoderma* and *Leptodophora* were present in both root and rhizome tissues (Fig. 1b; Table S2).

### Diversity indices across different parts of Wt and Tc plants

Alpha diversity differed significantly among tissue types, with one-way ANOVA/Welch tests showing significant differences for Observed species [F(4,10) : 64.79, p : 4.115e−07), Shannon index [F(4,10) : 241.66, p: 6.6947e−10)], and Simpson index [F(4,10): 489.62, p: 2.0214e−11], with tissue type used as the experimental factor across all samples (Fig. 1c–e). Beta diversity analysis based on Bray–Curtis dissimilarity revealed distinct clustering among tissue groups in the PCoA plot, with PERMANOVA confirming significant differences (F = 44.668, R^2^ = 0.947, *p* = 0.001) (Fig. 1f).

LEfSe analysis identified taxa that were differentially abundant among tissue types in Wt and Tc plants using the Kruskal–Wallis test (*p* < 0.05) with an LDA score cutoff of ≥ 2, and these taxa were considered potential tissue-specific biomarkers.Wt rhizomes were enriched with taxa including *Paraphoma*, *Albifimbria*, *Ilyonectria*, *Verticillium*, *Trichoderma*, *Polyscytalum*,* Leptodophora*, and *Brunneochlamydosporium*, whereas Wt root tissues were enriched with *Hemimycena*, *Hymenoscyphus*, *Eurotiomycetes*, *Agaricomycetes*, *Ceratobasidiaceae*, and *Hypocreales*. In Tc rhizomes, enriched taxa included *Paraleptospora*, *Myrmecridium*, *Diaporthe*, and *Acremonium*, while *Fusarium* was enriched in Tc root tissues. Additionally, several potential biomarkers belonged to incertae sedis, indicating unresolved taxonomic placement. (Fig. S2, Table S3).

The functional potential of the endophytic microbiota in both wild-type (Wt) and transgenic (Tc) *P. kurrooa* plants was predicted using PICRUSt2 software. Based on ITS sequence analysis and the KEGG pathway database, the putative functional capabilities of microbial communities were inferred. The predicted functions were primarily associated with metabolic pathways such as carbohydrate and amino acid processing, along with the synthesis and breakdown of terpenoids, polyketides, and energy metabolism. A substantial proportion of predicted genes were also associated with secondary metabolite biosynthesis, lipid metabolism, nucleotide metabolism, and xenobiotic biodegradation (Fig. 1g). Predicted microbial genes related to terpenoid backbone biosynthesis and phenylpropanoid-related pathways, which are components of plant picroside biosynthesis pathways (MVA, MEP, iridoid, and shikimate/phenylpropanoid pathways), were more abundant in the endophytic communities associated with Wt plants than Tc plants (Fig. S3). These results do not indicate direct microbial production of picrosides, but suggest a potential role of endophytic microbiota in influencing or supporting plant secondary metabolism.

### Isolation and phylogenetic diversity of cultivable endophytes

*P. kurrooa* plants collected from their natural habitat were used for the isolation of endophytic fungi. To ensure the rigor of the axenic isolation process, sterility checks of the final wash aliquot showed no microbial growth, confirming the effectiveness of the sterilization process. A total of 17 fungal isolates were obtained from different plant tissues: 4 from rhizomes, 5 from roots, and 8 from leaves. Pure cultures of these isolates were maintained and identified through amplification of the internal transcribed spacer (ITS) region. The resulting sequences were submitted to NCBI (Table S4).

Among the isolated fungi, the majority belonged to the phylum Ascomycota, with Dothideomycetes being the most abundant class, followed by Sordariomycetes and Leotiomycetes. Only one isolate was identified from the Basidiomycota phylum. These findings are consistent with amplicon-based diversity analysis, where Ascomycota also emerged as the dominant phylum. The ITS-based phylogenetic tree (Fig. S4), constructed using molecular data, revealed strong clustering of most isolates within Ascomycota, including *Fusarium* spp., *Trichoderma* sp., *Alternaria* spp., *Pezicula ericae*, *Cladosporium tenuissimum*, *Leptosphaeria* sp., *Ramularia cynarae*, and *Septoria* spp. In contrast, *Bjerkandera adusta* formed a separate branch under Basidiomycota. Within Ascomycota, clear sub-clustering was observed among *Fusarium* isolates (*F. acuminatum*, *F. tricinctum*, and two unidentified *Fusarium* sp.), indicating close genetic relatedness. *Trichoderma* sp. PKRF1, while clustering under Ascomycota, appeared more distantly related to the other genera, reflecting its distinct taxonomic lineage. Additionally, *Pezicula ericae* isolates grouped closely together, supporting species-level identification. *Alternaria alternata* and *Alternaria* sp., as well as *Septoria carvi* and *Septoria* sp., also clustered closely, indicating intra-generic diversity. Interestingly, *Trichoderma* was exclusively detected in wild-type (Wt) plants in the ITS-based amplicon diversity analysis. The phylogenetic placement of the isolate PKRF1 with other known *Trichoderma* sp. suggests its closest relation to *Trichoderma zeloharzianum* and *Trichoderma lentiforme* (Fig. S5). However, accurate species-level identification would require whole-genome analysis, which is discussed in the following section.

### In-vitro plant growth-promoting traits, screening of putative isolates for *in-planta* treatment

The qualitative and quantitative estimation of plant growth-promoting properties of the isolated was done. The phosphate solubilization capacity was reported in most of the isolated isolates, which ranged from 1.13 to 380.58 µg/mL, with the highest in PKRF1 (380.6 µg/mL), then by PKRF2 (360.3 µg/mL), PKFL8 (310.4 µg/mL), followed by other isolates (Fig. S6a, Table S5). Indole acetic acid production was also estimated in the fungal endophytes, which ranged from 2.26 to 4.75 µg/mL, wherein the highest production was reported in PKRF1 (4.75 µg/mL) and PKLF3 (4.56 µg/mL), followed by PKLF4 (4.015 µg/mL), PKRF4 (3.97 µg/mL), PKLF2 (3.91 µg/mL), and followed by PKRF1 (3.80 µg/mL) (Fig. S6b, Table S5). Similarly, in the case of siderophore, production ranged from 24.76% to 85.78%, with the highest production in PKLF1 (85.78%), followed by PKRF1 (83.96%), PKRF2 (78.22%), PKRF4 (70.99%), and other isolates (Fig. S6c, Table S5). Throughout the analysis, isolate PKRF1 exhibited promising plant growth-promoting properties. Consequently, it was selected for *in-planta* treatment, based on this evidence and its specific association with Wt plants identified through amplicon diversity analysis, to examine the impact of reintroducing this native endophyte on in vitro plants.

### *In-planta* treatment and colonization of PKRF1 in in-vitro propagated plants

**Fig. 2:**
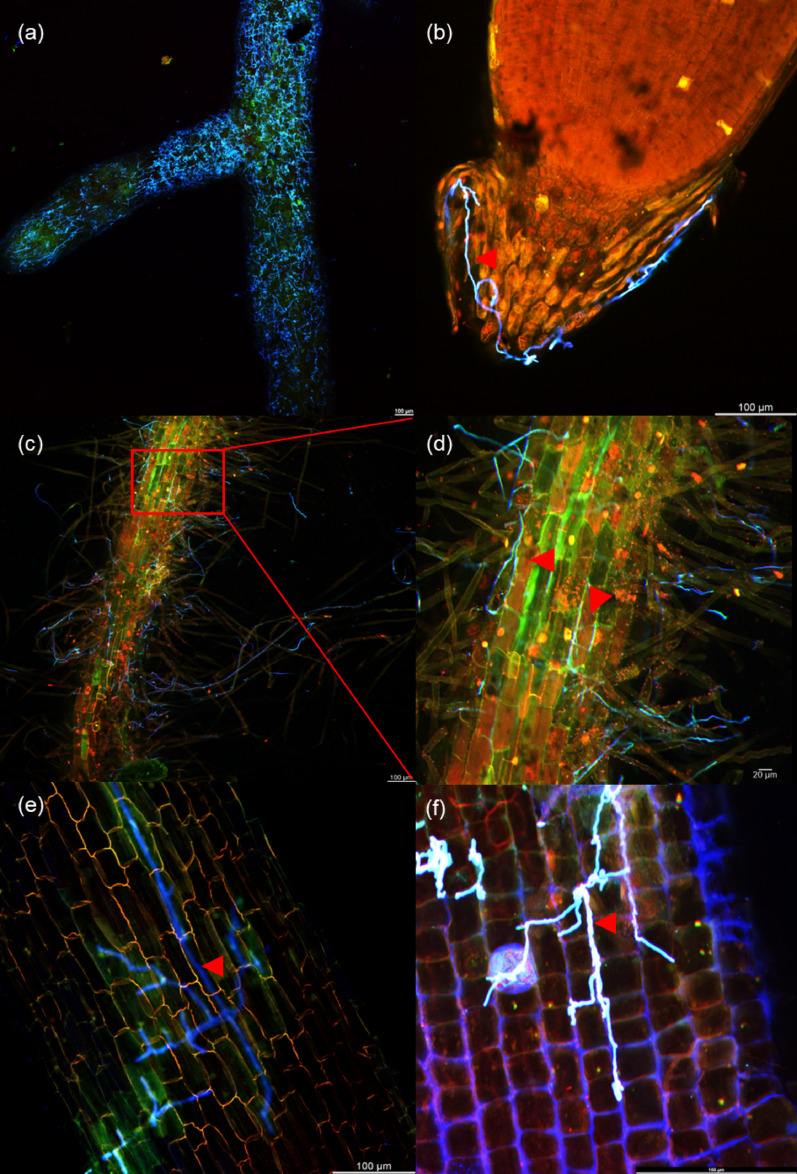
The colonization of PKRF1 was confirmed through confocal microscopy of the roots of *P. kurrooa* treated with PKRF1. The fungal mycelia were found to penetrate the cells of the root tissues. Fungal structures appear in blue after WGA-Alexa Fluor 488 staining, whereas root cell walls appear in red after propidium iodide staining. **a** Colonization at primary and lateral root junctions. **b** Fungal hyphae at root tip internalization. **c** The fungal hyphae forming coils wrapping around the root hairs, panel **d** shows the inset indicated by a *red-line square* in panel **c**, red arrows depict the internalization at the cell periphery by the fungal hyphae; **e** and **f** the fungal hyphae growth in the plant root suggesting colonization

To further confirm the effectiveness of the *in-plant* treatment and ascertain the true endophytic nature of PKRF1, colonisation of the fungus was confirmed through confocal microscopy. The mycelium layers were found to profusely cover the large surface of both the primary and lateral roots (Fig. 2a). The possible entry point was not evident, but the fungal hyphae were found to coil/attached around the root tips (Fig. 2b), which could be a potential way to enter the plant system through the rapidly multiplying cells of the roots. Further, the mycelia were observed to grow on the intercellular spaces, on the periphery of the cell walls (Fig. 2c and d), and possible cell penetration in some regions of the root tissue (Fig. 2e and f).

### Plant growth and biomass accumulation

**Fig. 3:**
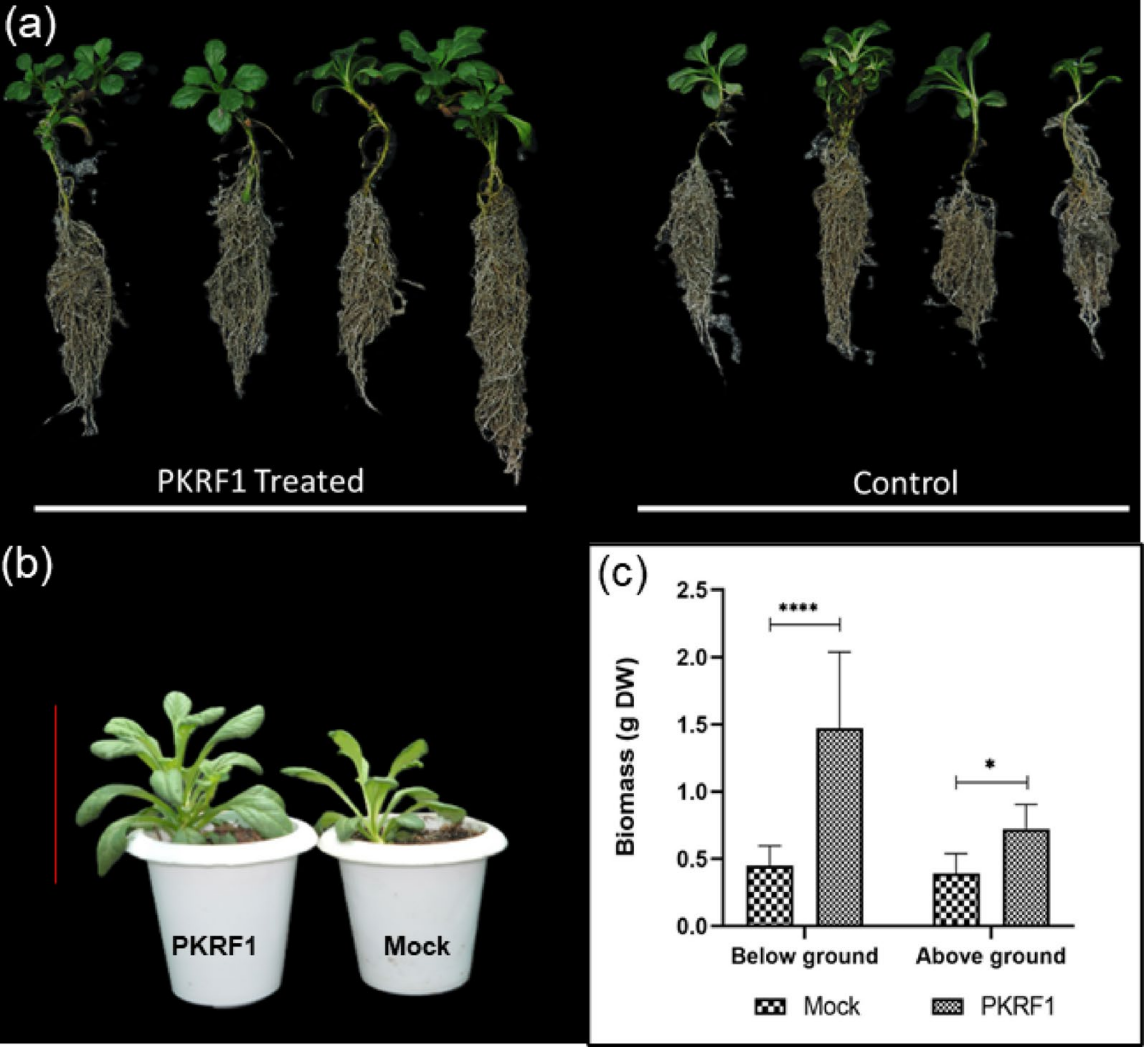
Effect of treatment on the in-vitro propagated plants of *P. kurrooa* with PKRF1. **a** and **b** and the estimation of below-ground and above-ground biomass. **c** of PKRF1 and mock (0.8% saline) treated plants. Statistical analysis for all the data shown in this figure was performed with “GraphPad Prism 8” using the Student’s t-test. Data are presented as mean ± standard deviation (SD) of five independent biological replicates, each consisting of pooled samples from eight plants per treatment. *p < 0.05, **p < 0.01, ***p < 0.001, ****p < 0.0001

Plants were destructively harvested 180 days after the booster treatment to evaluate growth and biomass accumulation, and PKRF1-treated plants showed significantly higher below-ground biomass (225.69% increase; Student’s t-test, *p* < 0.0001) and above-ground biomass (84.54% increase; Student’s t-test, *p* < 0.05) compared to mock-treated control plants (Fig. 3).

### Photosynthetic pigments, chlorophyll fluorescence and starch accumulation

**Fig. 4:**
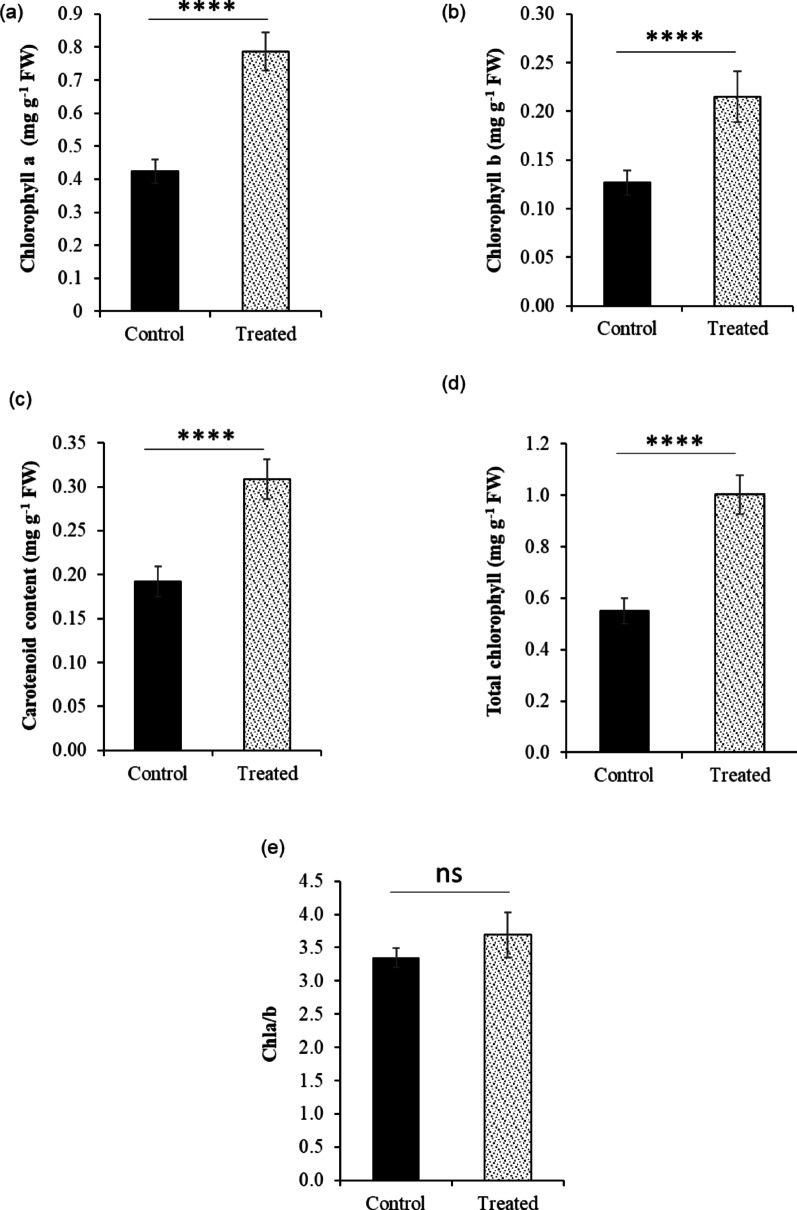
Estimation of photosynthetic pigments. **a** Chlorophyll a; **b** Chlorophyll b; **c** Carotenoid content; **d** Total chlorophyll (Chl a + b); **e** Chlorophyll a/b ratio. Statistical analysis for all the data shown in this figure was performed using GraphPad Prism 8 with Student’s t-test. Data are presented as mean ± standard deviation (SD) of five independent biological replicates, each consisting of pooled samples from eight plants per treatment. *p < 0.05, **p < 0.01, ***p < 0.001, ****p < 0.0001

**Fig. 5:**
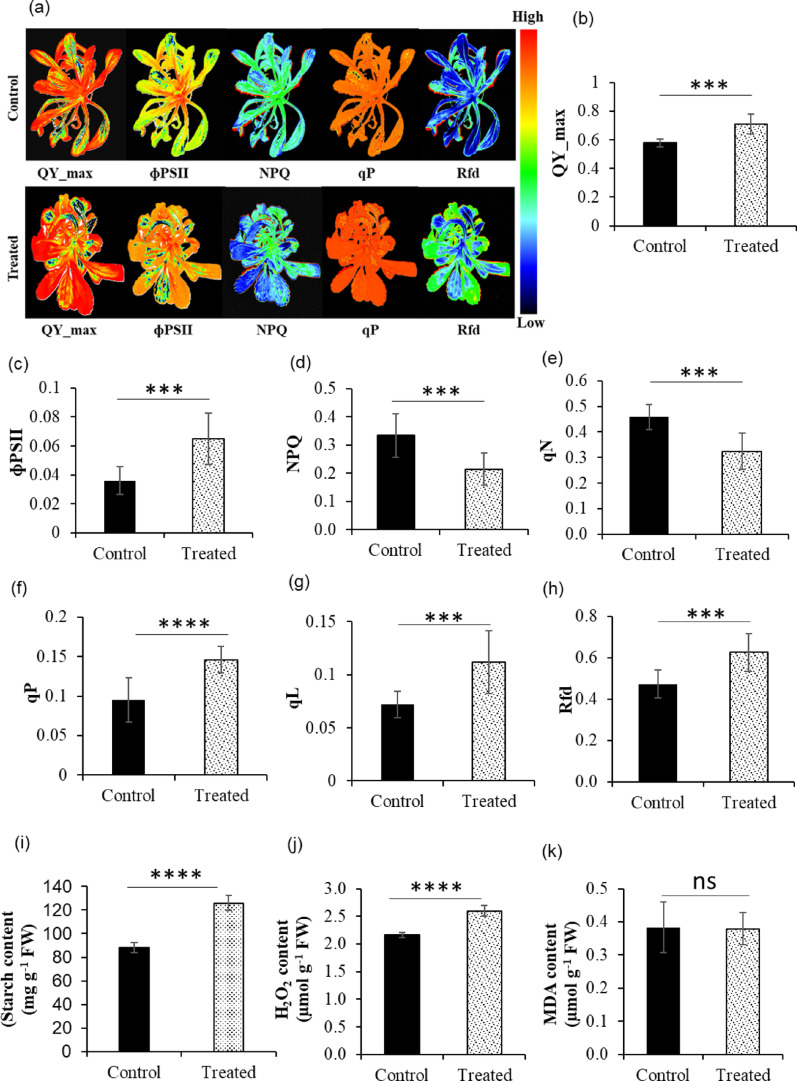
Effect of PKRF1 treatment on Chlorophyll fluorescence. **a** Chl fluorescence images of QY_max, ɸPSII, NPQ, qP, and Rdf in leaves of control and treated *P. kurrooa* plants. Quantification of chlorophyll fluorescence parameters. **b** QY_max, **c** ɸPSII, **d** NPQ, **e** qN, **f** qP, **g** qL, **h** Rfd, **i** Starch content **j** H_2_O_2_, **k** MDA. Statistical analysis for all the data shown in this figure was performed with “GraphPad Prism 8” using Student’s *t*-test: Error bars indicate mean ± SD. *p < 0.05, **p < 0.01, ***p < 0.001, ****p < 0.0001

The effect of PKRF1 treatment on the photosynthetic pigment revealed an increase in the content of Chl *a* (85% increase), Chl *b* (69% increase), Carotenoid (60%), and total chlorophyll (82% increase) compared to mock-treated plants. There was no significant difference in the ratio of Chl *a*/*b* between PKRF1-treated and mock plants (Fig. 4). The chlorophyll fluorescence was analyzed to investigate the effect of PKRF1 treatment on the photosynthetic activity of *P. kurrooa*. Different chlorophyll fluorescence parameters such as QY_max, ɸPSII, NPQ, qL, and qP, qN and Rfd were analysed. The quantum yield of photosystem II (QY_max) was significantly higher in PKRF1-treated plants, indicating improved photosynthetic efficiency. Additional parameters, including the effective quantum yield of PSII (ɸPSII), photochemical quenching (qP), and the fraction of open PSII reaction centers (qL), also exhibited significant increases in treated plants compared to the controls. The fluorescence decline ratio (Rfd), an indicator of photosynthetic performance, was also elevated in PKRF1-treated plants, reflecting enhanced photosynthetic activity. Conversely, the non-photochemical quenching (NPQ) and non-photochemical quenching coefficient (qN) were reduced in treated plants, suggesting a decrease in heat dissipation and a more efficient use of absorbed light energy for photochemistry (Fig. 5a–h). The accumulation of starch was also higher in PKRF1 treatment plants in comparison to control plants (Fig. 5i).

### Effect on H_2_O_2_ and MDA production

The effect of PKRF1 treatment was also investigated in the H_2_O_2,_ and MDA production to elucidate the effects of endophytes on plant stress responses. The production of H_2_O_2_ content was also higher in PKRF1-treated plants (Fig. 5j) in comparison to control plants; however, there was no significant change in the MDA content (Fig. 5k).

### UHPLC profiling to estimate secondary metabolites

**Fig. 6:**
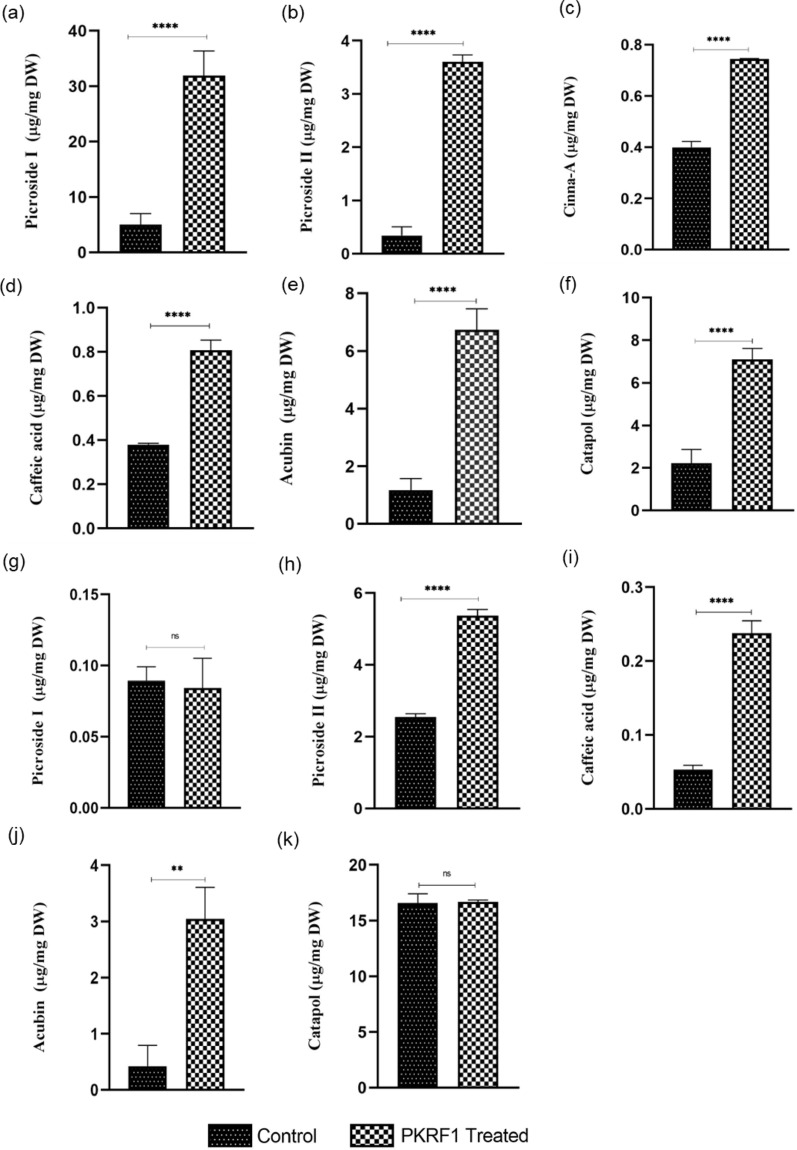
UHPLC estimation of secondary metabolite content in PKRF1-treated and mock plants. Leaf tissues: **a** P-I, **b** P-II, **c** cinnamic acid (Cinna-A), **d** caffeic acid, **e** aucubin, **f** catalpol; and root tissues: **g** P-I, **h** P-II, **i** caffeic acid, **j** aucubin, **k** catalpol. Statistical analysis for all the data shown in this figure was performed using GraphPad Prism 8 with Student’s t-test. Data are presented as mean ± standard deviation (SD) of five independent biological replicates, each consisting of pooled samples from eight plants per treatment. *p < 0.05, **p < 0.01, ***p < 0.001, ****p < 0.0001

To investigate the impact of PKRF1 treatment on the biosynthesis of secondary metabolites, ultra-high-performance liquid chromatography (UHPLC) profiling was conducted to measure the levels of picrosides (P-I and P-II) and their precursor molecules in leaf and root tissues of PKRF1-treated and control plants. PKRF1 treatment significantly influenced the biosynthesis of picrosides. In the leaf tissues, picroside-I (P-I) levels increased by 6.34-fold compared to the control, although no significant change was observed in the root tissues. For picroside-II (P-II), a marked enhancement was observed with a 10.4-fold increase in the leaf tissues and a 2.11-fold increase in the root tissues. These findings suggest that PKRF1 promotes the accumulation of picrosides, particularly in the leaf tissues (Fig. 6, Table S6). To further understand the effect of PKRF1 on the biosynthesis pathway, the levels of precursor molecules were measured. Caffeic acid, an intermediate in the phenylpropanoid pathway, showed a 2.12-fold increase in the leaf tissues and a 4.45-fold increase in the root tissues of PKRF1-treated plants and Cinnamic acid content increased by 1.86-fold in the leaf tissues, but it was not detectable in the root tissues (Fig. 6, Table S6). Precursors from the iridoid pathway were also analyzed. Acubin, an important intermediate, showed a 5.7-fold increase in the leaf tissues and a 7.2-fold increase in the root tissues of PKRF1-treated plants, and Catalpol, the immediate downstream intermediate of acubin and the backbone for picroside biosynthesis, exhibited a 3.2-fold increase in the leaf tissues. However, no significant changes were observed in the root tissues (Fig. 6, Table S6). The UHPLC profiling revealed that PKRF1 treatment significantly enhances the biosynthesis of picrosides and their precursor molecules. The effect was particularly pronounced in the leaf tissues, with notable increases in picrosides and their intermediates from the phenylpropanoid and iridoid pathways. These findings highlight the potential of PKRF1 in modulating secondary metabolite pathways, promoting the accumulation of bioactive compounds critical to the medicinal value of the plant.

### Expression of genes of picroside biosynthesis

**Fig. 7:**
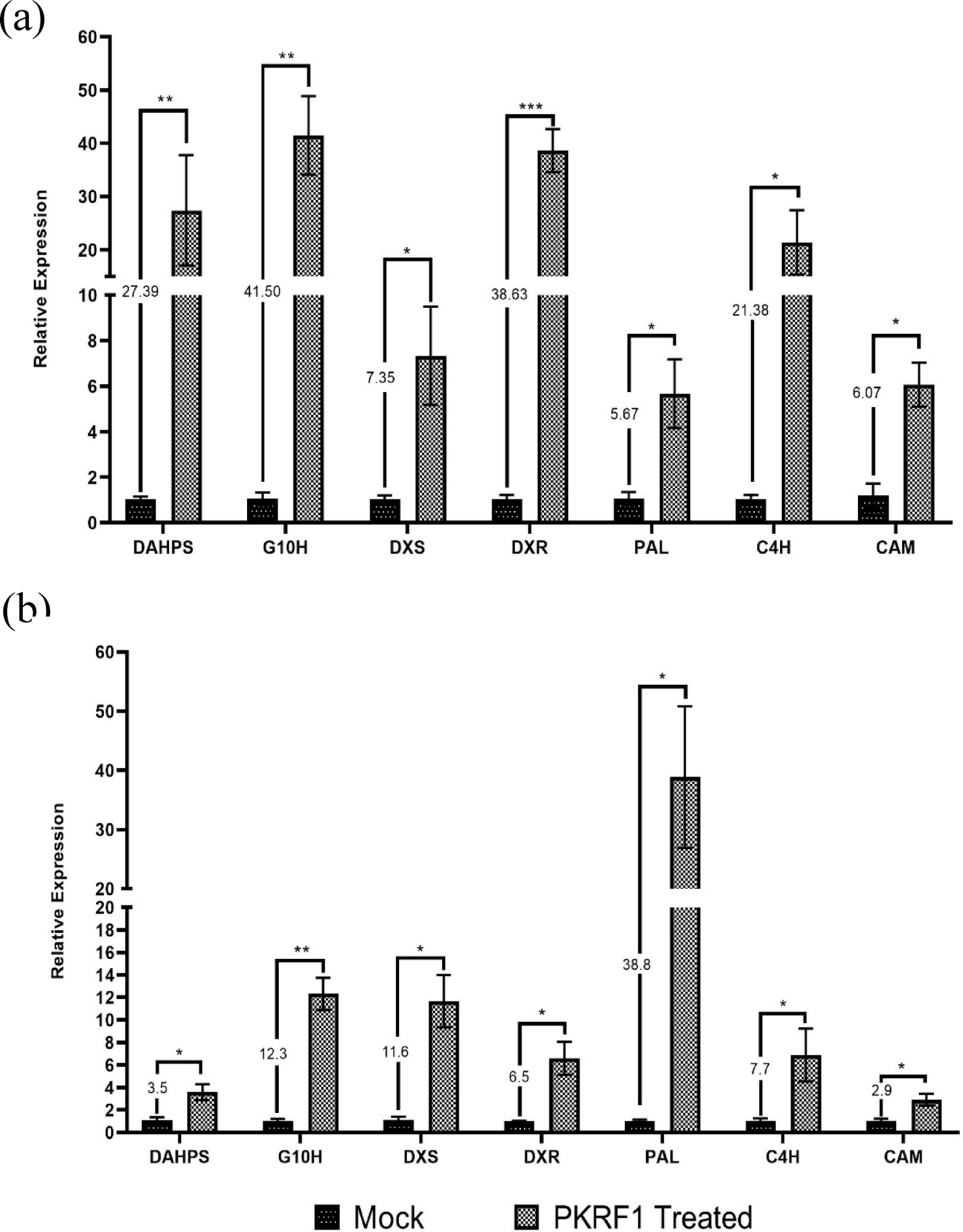
Expression analysis of key picroside biosynthesis genes in PKRF1- and mock-treated plants in **a** leaf and **b** root tissues. For normalization, the *P. kurrooa 26 S* gene was used as the endogenous reference gene, and the Y-axis represents relative quantity (RQ). Statistical analysis for all the data shown in this figure was performed using GraphPad Prism 8 with Student’s t-test. Data are presented as mean ± standard deviation (SD) of five independent biological replicates, each consisting of pooled samples from eight plants per treatment. *p < 0.05, **p < 0.01, ***p < 0.001

To further understand the role of PKRF1 in the modulation of picroside biosynthesis in the host plant, expression analysis of different genes involved in picroside biosynthesis was measured by quantitative real-time PCR (qRT-PCR). In leaf and root tissues different expression patterns were observed, with leaf tissues of PKRF1 treated plants having higher expression of *DAHPS* (27.39-fold), *G10H* (41.50-fold), *DXS* (7.35-fold), *DXR* (38.63-fold), *PAL* (5.67-fold), *C4H* (21.38-fold) and *CAM* (6.07-fold) whereas in root tissue the increased expression pattern involved *DAHPS* (3.5-fold), *G10H* (12.3-fold), *DXS* (11.6-fold), *DXR* (6.5-fold), *PAL* (38.8-fold), *C4H* (7.7-fold) and *CAM* (2.9-fold) (Fig. 7). Although the expression levels of all the genes were higher compared to the control plants, genes such as *DXS* and *PAL* showed notably higher expression, suggesting modulation of the associated biosynthetic pathway. Both leaf and root tissues of PKRF1-treated plants exhibited enhanced transcript levels of biosynthetic genes compared to the control. However, tissue-specific variations were evident: Leaf tissues exhibited higher expression of *DAHPS*, *G10H*, *DXR*, *C4H*, and *CAM*, aligning with the pronounced biosynthesis of picrosides in this tissue. Root tissues demonstrated a greater increase in *DXS* and *PAL* expression, suggesting a stronger activation of the early steps of the iridoid and phenylpropanoid pathways in roots. The qRT-PCR analysis revealed that PKRF1 significantly influences the transcriptional regulation of genes involved in picroside biosynthesis. The enhanced expression of key genes in leaf and root tissues highlights the systemic impact of PKRF1 treatment on the plant’s secondary metabolite pathways.

### Genome assembly statistics and phylogenomic

**Fig. 8:**
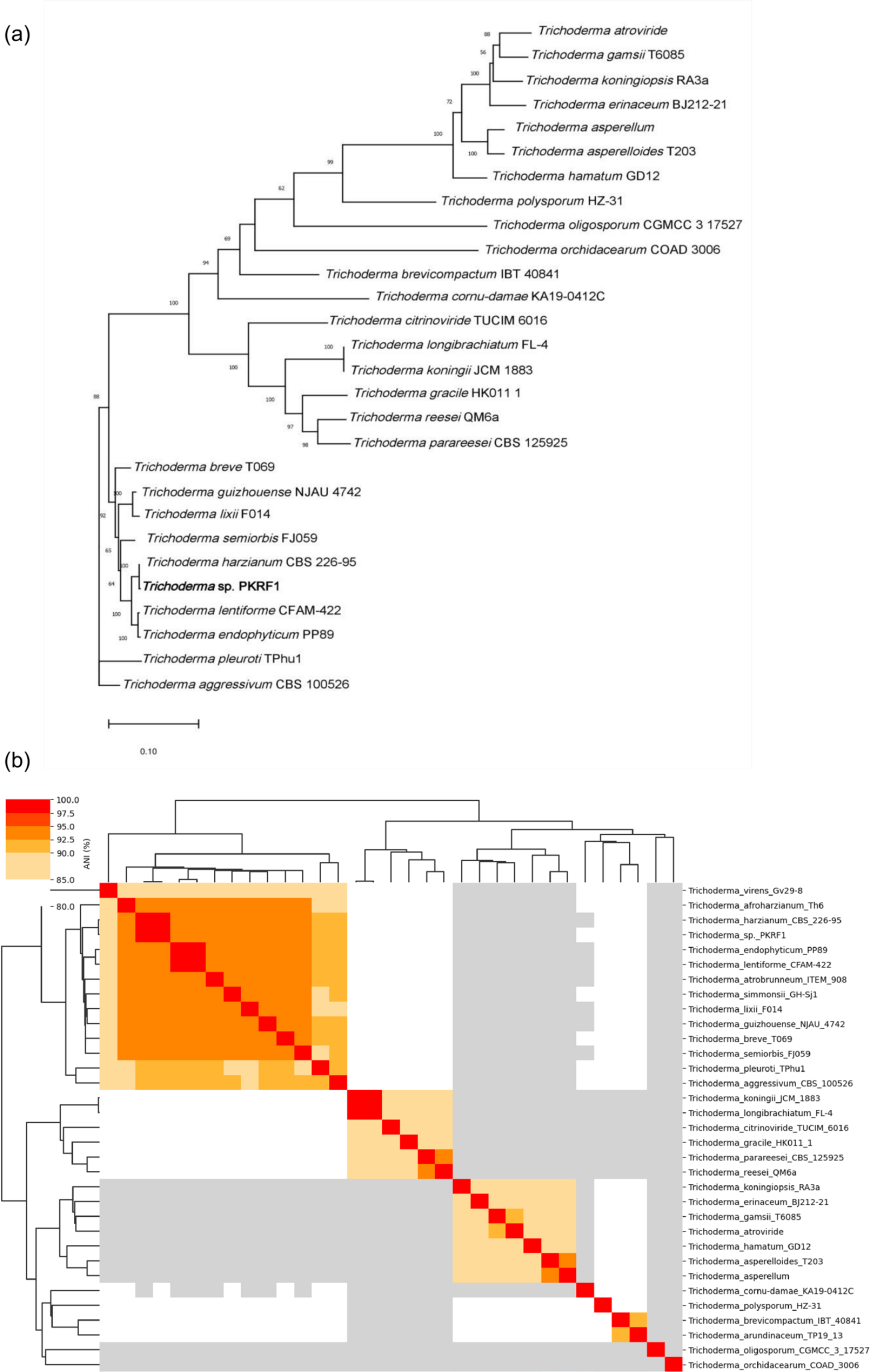
Genome-based phylogenetic analysis of PKTF1 **a** based on maximum likelihood phylogenomics generated using the UFCG tool with 1000 bootstrap replications; **b** ANI values and ANI-value-based hierarchical clustering evaluated by the FastANI tool plotted using the ANIclustermap tool

**Fig. 9:**
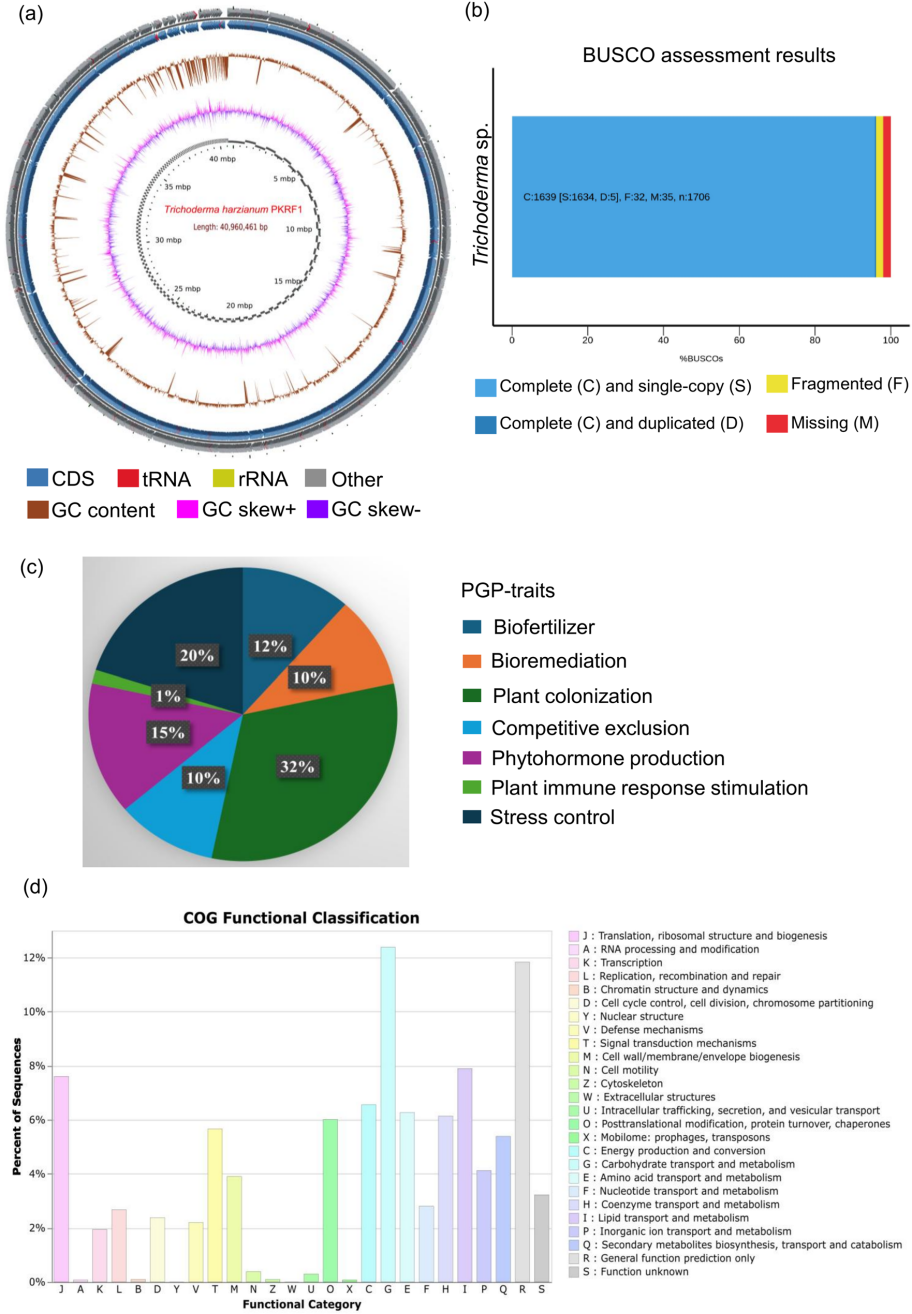
**a** Circular genome of PKRF1, depicting the colors depicting the CDS (blue), tRNA (red), rRNA (yellow), GC content (brown), GC skew+ (pink), GC skew – (violet); **b** BUSCO Assessment results; **c** Plant promoting properties annotated in PKRF1 genome; **d** COG annotation and classification

The whole genome of PKRF1 was 40.9 Mb in size with 100x coverage and G + C of 47.62% with 319 contigs and a total of 10,401 coding genes. The maximum likelihood phylogenomic tree generated using the UFCG tool with 1000 bootstrap replications revealed that the isolated PKRF1 belonged to *T*. *harzianum* species of *Trichoderma* genus (Fig. 8a). The ANI value between PKRF1 and *T. harzianum* CBS_226 − 95 was 99.5, confirming that the strain belongs to *T. harzianum* species (Fig. 8b). A circular genome representation of the strain is shown in (Fig. 9a) BUSCO analysis with the ascomycota_odb10 dataset showed that the fungal genome was 96.1, complete with 0.3 duplicates and 2 missing markers (Fig. 9b).

### Genome mining for PGP genes

The PKRF1 genome contained a unique set of genes which were not found in other non-endophytic *Trichoderma* strains, which were characterized based on their roles in promoting plant health. These genes are associated with functions such as plant colonization, competitive exclusion of other microbes, stress tolerance, and biocontrol activities, as detailed in Table S7. Specifically, genes involved in plant colonization were linked to the utilization of plant-derived glycosides and the degradation of plant glycosidases, facilitating effective root colonization. Competitive exclusion genes contributed to multidrug resistance and exopolysaccharide production, enhancing PKRF1’s ability to outcompete other soil microorganisms. Stress control and biocontrol-related genes played roles in scavenging reactive oxygen species (ROS) and synthesizing antimicrobial compounds. Moreover, PKRF1 harbors distinct genes associated with key biofertilizer functions, including nitrogen acquisition (*exoR*, *atzF*), phosphate and potassium solubilization (*gabD*), and phytohormone production (*amiE*). Additional genes support plant colonization (*iunH*, *nadE2*, *yncB*, *hypBA*, *deoC*, *add*), abiotic stress mitigation (*gst*), induction of systemic resistance (*ctyX*), and competitive exclusion (*TPO1*), highlighting the strain’s multifaceted role in promoting plant growth and resilience.

Further, the complete pan-genome analysis of *T. harzianum* PKRF1 was also performed to get an overall overview of PGP traits present in the strain. The analysis showed the presence of a diverse variety of plant growth-promoting genes in the PKRF1 genome (Fig. 9c). A detailed list of PGP genes present in the strain is shown in Table S8. The functional annotation based on COG, which helps in understanding the biological roles of genes within the genome, revealed highest percentage of sequence belonging to carbohydrate transport and metabolism, also approximately 5% belonged to secondary metabolite biosynthesis, transport, and catabolism (Fig. 9d). The pan-genome annotation of PKRF1 identified a total of 528 plant growth-promoting genes associated with biofertilization, plant colonization, stress responses to abiotic and biotic factors, and bioremediation or xenobiotic degradation. Among these, specific gene categories related to plant growth promotion were highlighted. Nitrogen acquisition genes included nitrogen fixation genes (*hyaD*,* hype*,* nifS*,* nifU*,* nifV*,* narK*,* frdA*,* graG*,* fixB*,* atzF*,* nirB*,* ureC*,* ureD*,* ureG*,* ycgI*,* ureF)* and other nitrogen acquisition genes *(nitA*,* nitB*,* nitR*,* exoR*,* glnA*,* uca*,* dur*,* urd*,* alc*,* amtB*,* fmdA*,* atzF*,* allB*,* gdhA*). Additionally, phosphate solubilization genes were identified, including *aceF*,* maeA*,* fmdA*,* frmB*,* dgoD*,* pqqL*,* dkgA*,* icd*,* Ira6*,* fumC*,* maeA*,* mdh*,* pckA*,* aco*,* acs*,* dml*,* prpE*,* dapA*,* dml*,* maeA*,* metC*,* gabD*,* icd*,* prpB*,* sucB*,* ilvD*,* paaF*,* ppa*,* ppk*,* phoA*,* phoD*,* phnY*,* pqqL*,* pqqI*,* and pqqF*. These findings underscore the genetic potential of PKRF1 in promoting plant growth through enhanced nutrient acquisition and stress resilience mechanisms. Additional genes involved in potassium solubilization were identified, including *gloB*,* prpF*,* gltA*,* icd*,* prpF*,* pqqL*,* pqqI*,* pqqF*,* prpE*,* hpaF*,* prpB*, and *alaA*. Genes responsible for siderophore production, such as *hemL*,* iroC*,* hemF*,* ddc*,* entB*,* hemB*,* hemC*,* and hemD*, were also present. Sulfur metabolism genes, including *cysK*,* ggt*,* ssuD*,* metB*,* nifS*,* sseA*,* cysQ*,* cysC*,* cysH*,* cysI*,* cysJ*, and *cysNC*, were annotated in the genome of PKRF1. Additionally, carbon dioxide fixation genes such as *cynT*, *icd*, and *cah* were also identified.

The ability to colonize the plant system by PKRF1 was also investigated, root colonization genes included *nucA*,* iunH*,* nadE2*,* ppnK*,* nadC*,* npdA*,* nunA*,* pntB*,* pncA*,* pncB*,* pncC2*,* surE*,* fadD*,* atoB*,* fabF*,* bioB*,* bioC*,* bioF*,* bioJ*, and other genes, along with root nodulation metabolism genes such as *maa*,* nodL*,* cycNC*,* glmS*,* alpA* and genes such as *glpA* and *glpK* which regulate root nodulation formation. Surface attachment-promoting genes included *gmd*,* manC*,* dltA*,* dltE*,* galT*,* wbA*,* wlbA*,* gmd*,* mpg*,* manC*,* manA*,* rfbB yfnG*,* rfbB*. Further, other genes included such as *xynD*, *cbhA*,* celJ*,* eglS*,* pglA*,* xynY*,* srfJ*,* rrrD*,* xynB*,* xynA*,* purA*,* pcaD* for degradation of plant-based molecules for efficient colonization through evading the defense mechanism of the host, nutrient acquisition, they help in penetration of the cell wall and promote a symbiotic relationship.

The other gene annotated in the genome of PKRF1 was further categorized into functional coding protein and its role in stress response and bioremediation. The genes mitigating stress response were also annotated in the genome of PKRF1, which were classified into two major categories, namely abiotic and biotic factors. Among the abiotic factors genes responsible for heat shock proteins (*clpB*, *dnaJ*, *dnaK*, *groEL*, *groES*, *hptG*, *hsp20*, high-temperature regulation genes (*grpE*), low-temperature sensitive genes (*gdh*, *yfhF*), osmotic stress genes (*betA*, *betC*, *mscL*, *trkD*, *speB*, *speC*, *nadC*, *nadA*), salinity stress-responsive genes (*chaA*, *NHA1*, *hxt*, *cjsJ*, *cysNC*, *lon*, *cysQ*, *czcO*, *trkD*, *argHA*, *argJ*, *proB*, *proC*, *otsA*,* fmt*,* folA. folKP*), oxidative stress-responsive genes (*acpS*, *fabF*, *ymfl*, crtQ, *yqjG*, *katE*, *qor*, *speB*, *speC*). In biotic factors annoted genes belonged to phytohormone production (*trpE/phnA*,* yodT*,* oxdA*,* pbuG*,* idi*,* mvaK2*,* mvaK1*,* CHO1/pssA*,* pgsA*,* speC*,* speB*,* folKP*,* folA*,* pabAB*,* pabC*,* glyA*,* purH*,* fmt*,* gcvT*,* thyA*,* purU*,* metF*,* ygfA*,* budA*,* ilvH*,* adh2*,* bdh*,* atoB*,* hydroxymethylglutaryl_CoA_synthase*,* desA*,* moaA*,* modF*,* hemA2*,* hemE*,* hemY*,* hemH*,* RIB2*,* FLAD1*,* panB*,* PPCS*,* coaE*,* acpS*,* ilvE*,* bkdA1*,* UPB1*,* adk*,* cytX*,* PPOX)* and plant signal transduction (*crtQ*,* trpS*,* fadD*,* PHO*,* acd*,* pdxH*,* qor*,* ipdC/ppdC*,* cfa*,* psd/PISD*,* budC*,* fabF*,* THI20*,* THI6*,* ydbC*,* serC/pdxF*,* qorB*). In the case of bioremediation, heavy metal detoxification genes such as cadmium and cobalt resistance (*czcD*), zinc resistance (*zntC*), arsenic resistance (*arsA*, *arsB*), copper resistance (*copA*, *cutC*), mercury resistance (*merA*), nickel resistance (*ddpB*, *hoxN*, *nixA*). PKRF1 also harbored genes involved in xenobiotic degradation, such as benzoate degradation (*chqB*, *catA*, *catE*), bisphenol degradation (*dad*, *hapE*), phenol degradation (*mobA*), organophosphate degradation (*opaB*, *pepP*).

### Secondary metabolite biosynthetic gene clusters

The secondary metabolite gene clusters were searched in the genome of PKRF1 strain and compared with non-endophytic *Trichoderma* strains. The list of clusters present in each strain is given in Table S9. Essentially, all the strains had a similar number of secondary metabolite gene clusters. The secondary metabolite biosynthetic gene clusters present in PKRF1 were further evaluated. The type and number of gene clusters present in the PKRF1 genome were as follows: Non-ribosomal Peptide Synthetase (NRPS) 3, NRPS-like 5, Type-1 Polyketide synthase (T1PKS) 17, fungal-RiPP-like 6, isocyanide-nrp 1, hybrid 11, and terpene 7. Out of all these gene clusters, there were two NRPS, five NRPS-like, nine T1PKS, six fungal-RiPP-like, one isocyanide-nrp, eight hybrids, and five terpenes novel gene clusters with no close homologs in the current databases are present in the PKRF1 genome (Table S10).

## Discussion

In recent years, there has been growing interest in exploring complete plant microbiomes through metagenomic approaches, which have revealed the presence of diverse plant-beneficial microbial communities. While these culture-independent methods provide valuable insights, their reliance on non-cultivable microbes limits their practical application. To overcome this, it is crucial to integrate metagenomic information with culture-dependent techniques, enabling the effective use of beneficial microbes in agriculture. In this study, we employed a dual strategy that combines both culture-independent and culture-dependent methods to comprehensively characterize the endophytic fungal communities associated with wild-type (Wt) and tissue culture-derived (Tc) *P. kurrooa* plants, with the aim of enhancing plant yield and picroside biosynthesis.

Plant-associated microorganisms have been widely recognized as key contributors to enhanced plant growth and stress resilience through their ability to improve nutrient availability, strengthen disease resistance, and mitigate environmental stressors via symbiotic relationships [54]. Domestication of wild plants provides an alternative to constant supply of raw materials; however, during this process, there is a loss of native microbial diversity associated with these plants and reintroduction of these lost communities could potentially help improve the plant health and overall fitness [55]. Several studies have found that the breakdown of mutualistic relationships between plants and microbes in cultivated crops has negatively impacted fungal colonization and reduced growth-promoting effects from symbiotic fungi [56]. The association of the microbiome with the host plant is a dynamic process developed through evolutionary relationships involving the interaction between microbe-to-host, host-to-microbe, and microbe-to-microbe. Endophytic fungi are known to inhabit very distinct biological niches and have been classified into highly diverse phylogenetic groups, which are capable of colonizing plant tissues asymptomatically without initiating any disease or exerting negative symptoms [57].

To understand the changes in the fungal diversity during the domestication of *P. kurrooa*, ITS-amplicon-based sequence was performed. The diversity analysis revealed a loss of diversity during the process of in-vitro propagation; the major taxa identified at the phylum level included Ascomycota, Basidiomycota, and some unclassified fungi. Earlier, the loss of endophytic bacterial community during in-vitro propagation of *P. kurrooa* has been established [10]. Similarly, reports have also been made in other crops, such as during in-vitro propagation of *Oleo europaea*, the overall number of OTUs decreased compared to plants generated using non-aseptic conditions [58]. The endophytic microbial community in wild rice cultivars had higher diversity and number of root endophytes compared to the cultivated counterparts in the first generation after cross-breeding. Additionally, the network analysis also revealed that the fungal communities in wild plants formed closed correlation clusters in comparison to the cultivated plants [59].

Most of the classified fungi belonged to the Ascomycota phylum; this has been corroborated in many other investigations, including medicinal plants such as *Arnebia euchroma* [23], *Vaccinium ovatum* [60] and *Dysosma versipellis* [61], among many others. Following this, Basidiomycota was a major identified phylum; many fungi from this phylum have been known to show endophytic properties in many plants. The other fungi present in the *P. kurrooa* belonged to an unclassified fungus, with Wt plants having a higher percentage of this unclassified fungus, suggesting the presence of many fungi that remain to be identified and their possible potential role in plant synergism. Approximately 420,000 plant species are known to exist in nature, and only a few have been explored for endophytic diversity [62]. The number of unidentified fungal species is believed to be very high. Although approximately 120,000 species have been described so far, the total number of fungal species is estimated to be between 2.2 and 3.8 million. This implies that over 90% of fungal species are yet to be discovered. This disparity underscores the vast diversity of fungi and the considerable effort required in mycology to thoroughly document these organisms [63].

The endophytic diversity at the genus level included *Albifimbria*, the endophytic nature of this genus has been reported in other plants, too, such as in grapes, where it imparts biocontrol properties against gray mold disease-causing fungus *Botrytis cinerea* [64], from *Coptis chinensis*, a medicinal plant, where it was found to produce pyrrole alkaloid which has immunosuppressive properties [65]. *Ilyonectria* is reported as an endophyte with growth promotion, along with increasing the accumulation of bioactive molecules [66]. *Paraphoma* is a common soil-borne pathogen; however, in many plants, endophytic nature and plant-benefiting roles have been discovered, such as increasing plant growth and improving tolerance to salt stress, drought stress, and heavy metals [67, 68]. *Polyscytalum* is known to be associated with many plants as an endophyte [69]. Further, the *Verticillium* genus is known to cause wilt disease in many crops such as potato and mint; however, it also colonizes endophytically to sympatric host plants such as mustard and grasses, suggesting evolution from pathogenic to endophyte [70]; its endophytic nature is also reported in other plants such as in sunflower [71]. Similarly, *Fusarium* is known to be pathogenic in many plants; however, its endophytic nature has also been well established in many plants, such as an endophytic *F. decemcellulare* isolated from rice, with antifungal properties by producing specialized secondary metabolites [72], whereas *F. equiseti*, an endophyte isolated from roots of *Vicia villosa*, revealed growth-promoting attributes and reduced the severity of root rot diseases in pea [73]. *Ceratobasidiaceae* family of fungi has been reported as an endophyte in plant species with many plant-beneficial properties [66]. Similarly, the *Eurotiomycetes* class of fungi is also known to be associated as an endophyte [74]. Among the different taxa only present in wild plants, one major identified taxa was *Trichoderma*; this genus is a versatile filamentous ascomycete that has a wide range of habitats, including soil, as saprophytes in woods, and as a parasite on fungi, animals, and insects [75, 76]. Although it shows diverse habitat selections, its colonization of plants and endophytic nature has been reported in many plants such as *Pandanus* sp [77]., *Triticum aestivum* [78]. *Hemimycena* is a fungus belonging to the Mycenaceae family and has been specifically associated with the root of the olive plant [79]. *Cyphellophora*, belong to the *Herpotrichiellaceae*, within the order *Chaetothyriales*, and is reported to be associated with plants as an endophyte [80]. *Leptodophora* colonizes roots as an endophyte in a diverse range of plants [81, 82]. *Hymenoscyphus* is a fungus of the Helotiaceae family, and some species of this genus are known to be pathogenic to plants, while some species are known to be endophytes with synergistic activity [83]. Similarly, *Preussia* genera are known to be associated with a diverse range of plants as endophytes [84] with many plant growth promotion attributes [85].

The presence of these diverse fungal communities with many plant-beneficial properties suggests the synergistic association of these communities with the host plants, which also suggests the possible role of these endophytes in health and fitness in the case of *P. kurrooa*. Endophytes spend most of their life span inside the host plants, providing a large and complex micro-habitat environment in the intercellular spaces of plant tissue [86]. The roots serve as pivotal entry points for microbes, where colonization is initiated by root exudates that shape microbial community diversity; these below-ground microbial communities, in turn, synergistically interact with plants and provide beneficial properties that support plant growth and health [87, 88]. Our investigation supported this finding, as we observed that fungal endophytes isolated from below-ground plant parts exhibited plant growth-promoting (PGP) traits in vitro assay. Among the various isolates, PKRF1 was selected for *in-planta* treatment to evaluate its effectiveness in promoting plant growth and enhancing secondary metabolite biosynthesis in *P. kurrooa.*

The increase in the plant biomass on treatment with PKRF1 observed in the present investigation could be due to the multifaceted plant growth-promoting attributes of the strain, such as the mobilization of micronutrients, phytohormone production, and siderophore production. There are many significant investigations suggesting the efficacy of *Trichoderma* sp. in increasing plant growth. An endophytic *Trichoderma gamsii* isolated from roots of lentils growing in the Himalayan region showed growth promotion in different crops and provided biocontrol properties [89]. Combined application of *T. asperellum* and *T. harzianum* enhanced plant growth while providing defense against *Colletotrichum truncatum* through increased cell wall lignification and activation of defense-related genes [90]. The phosphate solubilization attribute of *Trichoderma* isolates is well documented; among the different strains isolated from the Amazon rainforest, about 19.5% of the isolated strains were able to solubilize phosphorus and showed growth promotion in soybean plants [91]; and this attribute is also reported in other studies [92, 93]. Other PGP attributes observed in PKRF1 included IAA and siderophore production; this property of the *Trichoderma* genus has been reported in many other investigations, suggesting a multifaceted role of this fungus and its close association with the plant, which is defined by the multiple PGP traits it harbors [94, 95].

The *in-planta* treatment of PKRF1 on in-vitro propagated plants revealed an increase in the plant biomass, particularly the root biomass, which was enhanced in treated plants; the observation suggests the growth enhancement attribute of PKRF1 on *P. kurrooa*, which can be explained by the plant growth-promoting attributes of the strains. The production of IAA by *Trichoderma* stimulates the ethylene biosynthesis catalyzed by ACC synthase; the application of *T. guizhouense* NJAU 4742 strain on cucumber seedlings increased the plant biomass, modified the root architecture, and increased the lateral root tips by 64.7% in comparison to controls [96]. Additionally, the in-situ biosynthesis of auxin by *T. guizhouense* during the interaction with the roots showed a gradual increase in the growing media in treated plants compared to control plants. These findings suggest that the interaction with host roots significantly increased the exogenous production of IAA by *Trichoderma*, which could be one of the underlying reasons behind plant growth promotion. The genus also produces several proteins and metabolites with target-specific components regulating different physiological responses, viz., 6-pentyl-α-pyrone (6PP), which improved plant height, root system, and lycopene content in fruits [97], xylanase elicited ethylene biosynthesis [98], isoharzianic acid improves seed germination [99], harzianolide which promotes seedling growth [100], hydrophobin induces systemic resistance and stimulates root formation and growth [101], harzianic acid enhanced plant growth and increased the seed germination rate [99, 102, 103] and cremenolide, which promotes plant growth [104]. *Trichoderma* can regulate plant growth either directly through the molecules it releases or indirectly by altering the surrounding environment: it can modify the soil microbiome or reduce soil pH, thereby increasing the availability of macro- and micronutrients to plants [105]. Many such reports suggest the role of *Trichoderma* spp. in modulating physiological, biochemical, and molecular mechanisms in a wide range of plants growing under different growth conditions; all these properties indicate the huge potential of the members of this genus to be utilized as biofertilizers, bio-stimulants, and biocontrol agents in agriculture [106, 107].

Further, to understand the effect of PKRF1 on the photosynthesis activity of the plant, we measured the different photosynthetic parameters and pigments in the PKRF1-treated and control plants. The photosynthetic parameters estimated included QY_max (maximum yield of photosystem II), ɸPSII, NPQ, qP, qL, qN, and Rfd. QY_max, which indicates the efficiency by which the photons are used to drive the photochemistry in PSII, especially the conversion of light energy into chemical energy during the process of water splitting and oxygen evolution; in our observation, the PKRF1-treated plants have higher QY_max indicating a higher efficiency of utilizing the absorbed light photochemistry in PSII, which directly relates to production of more carbohydrates and essential for increasing plant biomass. In the case of the effective quantum yield of photosystem II (PSII) (ɸPSII), which provides a snapshot of how effectively PSII is converting absorbed light into chemical energy under actual light conditions, reflecting real-time photosynthetic performance, we also observed that PKRF1 treated plant showed higher value in comparison to the control plants, a low ɸPSII value typically suggests reduced photosynthetic efficiency. Some plants have high phenotypic plasticity, meaning they can adjust their physiology and morphology to different environmental conditions, including treatment with endophytes, which could provide overall fitness by regulating these parameter [108]. Further, Nonphotochemical chlorophyll fluorescence quenching (NPQ) is defined as the process through which excess light energy is dissipated into heat, which takes place in the photosynthetic membrane of the plants [109, 110]. The lower NPQ values in treated plants imply better resilience to stress, likely due to enhanced water and nutrient uptake facilitated by PKRF1 treatment [111]. *Trichoderma* are reported to enhance plant resilience by improving nutrient uptake, increasing water efficiency, or producing stress-related metabolites, thus reducing the overall stress load on the plant, including better photosynthetic efficiency [112]. This suggests that PKRF1 plants are better able to use absorbed light energy for photosynthesis rather than dissipating it as heat. Moreover, higher qP and qL values in treated plants demonstrated increased photochemical activity as more PSII reaction centers were actively engaged in photochemistry [113, 114]. This improvement could be due to several factors, including enhanced chloroplast function, better light utilization, or an overall healthier physiological state. The increased photochemical quenching in endophytic-treated crops, alleviating the plants against stress conditions, has been reported in earlier investigations [115, 116]. The treated plants also showed lower qN values, indicating reduced reliance on non-photochemical quenching for energy dissipation [117], which aligns with reduced physiological stress and more efficient energy use [118]. Finally, the Rfd, a marker of photosynthetic efficiency and plant vitality, was significantly higher in PKRF1-treated plants. This indicates enhanced photosynthetic performance and suggests that the treatment positively impacted plant growth and metabolism [119]. The increased starch levels observed in PKRF1-treated plants further support the enhancement of photosynthetic activity and plant growth. Evidence suggests that endophytes can significantly influence the accumulation of photosynthetic products, including starch, by improving photosynthetic efficiency and metabolic processes [120]. These collectively demonstrate that PKRF1 treatment improves photosynthetic efficiency by enhancing energy utilization, reducing stress-related losses, and promoting overall plant vitality and productivity.

The estimation of H_2_O_2_ content in PKRF1-treated plants revealed an elevated level, which may signify a controlled oxidative burst, a signaling mechanism triggered by the fungus-plant interaction [121]. This increase in H_2_O_2_ likely reflects an enhanced basal defense status, preconditioning the plant to cope with potential stress. Such preconditioning is a hallmark of the systemic resistance often induced by beneficial endophytes [122]. Studies suggest that endophytic symbiosis between plants and fungi mediates a heightened efflux of H_2_O_2_, which is essential for establishing root endophyte-plant interactions [123]. Moreover, this H_2_O_2_ signaling mechanism has been linked to the enhancement of secondary metabolite biosynthesis in plants, a process vital for growth and stress resilience [124, 125]. Interestingly, no significant difference was observed in the malondialdehyde (MDA) levels, indicative of lipid peroxidation, oxidative stress, and cell membrane injury [126]. Similar findings have been reported in previous studies, where endophyte-treated plants showed no significant change in MDA levels compared to controls [127]. However, in some cases, an initial rise in MDA levels during endophyte inoculation was followed by a notable reduction at later stages, suggesting a transient oxidative response that stabilizes over time [128]. These observations highlight the complex interplay between oxidative signaling and plant-endophyte interactions, contributing to enhanced plant defense and secondary metabolite production.

The impact of PKRF1 treatment on secondary metabolite biosynthesis was evident, with a significant increase in P-I content observed in leaf tissues but no notable difference in root tissues. Conversely, P-II content was elevated in both leaf and root tissues. Previous studies have established that the biosynthesis of picrosides is site-specific, with P-I synthesized in leaves and P-II predominantly in root tissues, followed by accumulation in the below-ground parts of the plant [129]. However, there are also reports indicating the presence of P-II in leaf tissues [130]. The increased accumulation of P-II in leaf tissues observed in the present study may be attributed to the translocation of picrosides from roots to aerial parts through the vascular system, along with possible endophyte-mediated modulation of metabolite biosynthesis and distribution; however, further studies are required to elucidate the precise mechanisms governing tissue-specific biosynthesis and accumulation of these compounds. In the current study, the precursors involved in the phenylpropanoid and iridoid pathways showed higher accumulation in PKRF1-treated plants. These findings suggest that reintroducing endophytes, such as PKRF1, could play a critical role in enhancing secondary metabolite production in medicinal plants. Further analysis of key genes associated with picroside biosynthesis in both leaf and root tissues revealed significant upregulation, providing molecular evidence for enhanced metabolite production. The role of endophytes in modulating secondary metabolite biosynthesis is well-documented. These observations underscore the potential of employing endophyte-mediated strategies for the sustainable production of valuable secondary metabolites in medicinal plants. The role of the *Trichoderma* genus in enhancing secondary metabolite production in various crops has been widely documented. For instance, extracts from endophytic *Trichoderma atroviride* have been shown to promote hairy root growth and stimulate tanshinone biosynthesis in *Salvia miltiorrhiza* by regulating key genes involved in tanshinone biosynthesis [131]. Similarly, in *Withania somnifera*, co-inoculation of *T. viride* with other endophytes significantly increased both plant biomass and withanolide content [132].

The picroside biosynthesis pathway comprises multiple interconnected pathways, including the non-mevalonate (MEP), mevalonate (MVA), phenylpropanoid, and iridoid pathways [133]. Enzymes within these pathways play pivotal roles in regulating picroside biosynthesis. Disruption of these enzymes has been shown to influence the overall metabolic flux, leading to deficiencies in precursor molecules required for picroside production [134, 135]. In PKRF1-treated plants, significant upregulation of key biosynthetic genes was observed, including *DAHPS*, *G10H*, *DXS*, *DXR*, *PAL*, *C4H*, and *CAM*. *DAHPS* (3-deoxy-D-arabino-heptulosonate 7-phosphate synthase) catalyzes the first committed step in aromatic amino acid biosynthesis, serving as an entry point into the phenylpropanoid pathway. This enzyme is primarily active in the chloroplast, and its transcript abundance has been positively correlated with increased picroside content [136]. *G10H* (geraniol 10-hydroxylase), a cytochrome P450 enzyme, catalyzes the hydroxylation of geraniol to 10-hydroxygeraniol, a rate-limiting step in iridoid monoterpenoid biosynthesis, which is crucial for picroside production [137, 138]. Additionally, *DXS* and *DXR* are key enzymes of the MEP pathway that regulate the formation of isoprenoid precursors, IPP (isopentenyl diphosphate) and DMAPP (dimethylallyl diphosphate), which are necessary for geraniol biosynthesis and downstream picroside production [139]. *PAL* (phenylalanine ammonia-lyase) catalyzes the deamination of phenylalanine to trans-cinnamic acid, an immediate precursor of picroside-I. This enzyme exhibits the highest expression in leaves, correlating with elevated picroside accumulation in this tissue [140]. Similarly, *C4H* (cinnamate-4-hydroxylase) catalyzes the conversion of cinnamic acid to 4-coumaric acid, a critical intermediate in phenylpropanoid and picroside biosynthesis. Increased transcript levels of *C4H* have been linked to higher picroside accumulation [141, 142]. Finally, *CAM* (caffeic acid O-methyltransferase) catalyzes the methylation of caffeic acid to ferulic acid, which serves as the precursor for vanillic acid, a key metabolite in the synthesis of picroside-II [143]. These findings highlight the integral role of these enzymes in facilitating the biosynthesis of picrosides and the potential of PKRF1 treatment to enhance their production.

The role of PKRF1 in stimulating metabolite production can be elucidated through the dynamic functions of *Trichoderma* and its regulatory influence on various host factors. The *Trichoderma* genus is well-recognized for triggering salicylate (SA) and jasmonate/ethylene (JA/ET)-mediated signaling in host plants, which, in turn, induces systemic resistance mechanisms [144]. These phytohormones significantly contribute to metabolite synthesis and act as potent elicitors, enhancing secondary metabolite production in numerous medicinal plants [145]. For instance, SA and JA signaling have been shown to stimulate picroside biosynthesis [146]. Previous studies have reported that during the colonization of *Trichoderma* in *Arabidopsis* roots, several plant genes involved in metabolism were differentially regulated. These included genes associated with ethylene and jasmonic acid (JA) signaling pathways, such as WRKY41, WRKY55, ERF1, and ERF6; JA-specific signaling genes like WRKY18, WRKY40, WRKY33, JIN1-7, and TDR1; and genes involved in the biosynthesis of secondary metabolites, such as MYB51 [147]. Additionally, genes involved in the phenylpropanoid pathway, such as phenylalanine ammonia-lyase (*PAL1* and *PAL2*) and *4CL*, exhibited increased expression. These enzymes are crucial to picroside biosynthesis, with their upregulation positively correlating with enhanced picroside production [133, 148]. These findings provide valuable insights into the mechanisms by which PKRF1 regulates picroside biosynthesis and its potential application in enhancing its production. Furthermore, this highlights the significance of reintroducing lost endophytic fungi to promote secondary metabolite biosynthesis, paving the way for integrating additional endophytic microbes into such strategies.

Notably, PKRF1 inoculation resulted in a concurrent increase in plant biomass and enhanced accumulation of secondary metabolites, indicating a positive influence on overall plant growth and metabolic activity. Traditionally, increased metabolite production has been considered to impose a metabolic trade-off that may affect plant growth; however, recent studies suggest that endophytes can play a dual role in promoting both biomass accumulation and secondary metabolite production. This could be supported by the fact that nearly 90% of plant dry matter is derived from photosynthetic carbon fixation, which provides the primary precursors for secondary metabolite biosynthesis [149]. Endophytes are known to enhance photosynthetic efficiency and growth in medicinal plants, thereby supporting higher biosynthesis of secondary metabolites [149, 150]. Similar observations of simultaneous stimulation of plant growth and metabolite production by endophytes have been reported in different studies [151, 152]. Nevertheless, the long-term effects of sustained enhancement of biomass and metabolite production on plant fitness remain unclear, and further ecological and physiological studies are required to evaluate the potential metabolic costs associated with prolonged endophyte-mediated stimulation of secondary metabolite biosynthesis Further, to understand the genomic evidence of the fungal strain and to understand the plant growth promotion, plant colonization, and secondary metabolite enhancement, whole genome sequencing of the endophytic fungus was carried out. Phylogenetic analysis identified the isolated strain as *Trichoderma harzianum*. This study represents the first report of an endophyte from this genus associated with *P. kurrooa*. The identification of PKRF1 was conducted following the molecular guidelines established [153], which recommend using three DNA barcodes, ITS, tef1, and rpb2, for precise *Trichoderma* classification. The average nucleotide identity (ANI) allows comparative measurements between genomes and measures the nucleotide-level genomic similarity between genomes, and a cutoff of 95% is used to group genomes at the same species level [154, 155]. Genomic analysis revealed the presence of genes associated with multiple plant growth-promoting functions, including nitrogen acquisition, phosphorus and potassium solubilization, iron acquisition/siderophore production, sulfur metabolism, and carbon fixation. The presence of these functionally important genes in PKRF1 supports its plant growth-promoting attributes in *P. kurrooa*, consistent with previous reports describing their roles in enhancing plant growth [156]. Further evidence supporting the association with endophytic fungi is their ability to colonize plant roots. PKRF1 harbors genes associated with root colonization, including the niacin (vitamin B3) biosynthesis gene cluster, which produces precursors of nicotinamide adenine dinucleotide (NAD^+^) and nicotinamide adenine dinucleotide phosphate (NADP^+^), molecules that regulate several downstream processes promoting root colonization [157] and root nodulation [88]. During colonization, microbes first attach to root surfaces before invading root hairs and tips; in *Trichoderma*, genes related to surface attachment and adhesion play a key role in this initial phase [158]. The genomic evidence of its endophytic nature is also highlighted by the presence of genes to utilize plant-based substrate and degradation of complex carbohydrates such as glucosidase, cellulase, hydrolase, mannosidase, along with amino acids, lipid metabolism, benzoate, and lignin utilization, which have been reported in many investigations as a strategy utilized by endophytes for their establishment in the host plant [159, 160]. Among the other genes annotated belonged to abiotic/biotic stress tolerance; this aspect has been particularly well established in the case *of the Trichoderma* genus, which shows the multifaceted role of this endophyte in the overall synergistic role in plant health and fitness [161, 162]. The bioremediation potential of PKRF1 has also been established, offering additional benefits, particularly in aiding the establishment of Himalayan plants in non-niche areas, as supported by other studies highlighting this important characteristic [163, 164].

To further investigate the potential of PKRF1 in enhancing picroside biosynthesis, antiSMASH analysis was conducted to identify secondary metabolite biosynthetic gene clusters within its genome. The analysis revealed the presence of gene clusters associated with NRPS, T1PKS, and terpene biosynthesis, which are linked to the production of various metabolites with plant growth-promoting and therapeutic benefits. Metabolites such as choline, which are synthesized by filamentous fungi for their growth [165], are also known to enhance plant growth and stress tolerance when produced by endophytes [166]. Metachelin, a specific class of siderophores, plays a role in iron acquisition for fungi while making iron more bioavailable to plants, thus promoting plant growth [96]. Dichlorodiaporthin, a polyketide compound, exhibits a range of plant-beneficial biological activities [167, 168]. Similarly, harzianopyridone, originally isolated from *Trichoderma*, is known for its antifungal properties and has recently been found to possess multiple therapeutic and plant-beneficial activities [169, 170]. Clavaric acid, a steroid produced by fungi, exhibits antibacterial and antitumor properties [171]. Tricholignan A, a redox-active ortho-hydroquinone, is involved in reductive iron assimilation in plants [172]. Other metabolites, such as harziphilone (a polyketide-derived azaphilone), display diverse and potent biological activities [173], while decumbenones contribute to plant growth and biocontrol capabilities [174]. Depudecin, an inhibitor of histone deacetylase produced by *Alternaria porri*, has both antitumor properties and biocontrol potential [175]. Intriguingly, reports suggest that metabolites produced by endophytes can enhance the therapeutic efficacy of their host plants, adding to their medicinal potency [176]. Trichobrasilenol, synthesized by sesquiterpene cyclase through farnesyl diphosphate rearrangement, has the potential to serve as a precursor for numerous terpenoids [177, 178]. Additionally, squalestatin has been noted as a metabolic pathway switch, regulating secondary metabolite biosynthesis for many bioactive compounds [179]. The diverse array of metabolites produced by PKRF1 underscores its role in promoting plant growth and enhancing the therapeutic properties of its host plants, highlighting its biotechnological and ecological significance.

## Conclusion

Thus, this study highlights differences in endophytic fungal communities between Wt and Tc plants of *P. kurrooa*, including the loss of specific fungal taxa during micropropagation, which may be associated with the observed decrease in metabolite content in Tc plants. The insight generated from these could potentially provide valuable opportunities for utilizing the diverse endophytes from the wild plants to enhance both vegetative growth and metabolic processes in the cultivated plants. The promising endophyte PKRF1, a *Trichoderma* species exclusively associated with Wt plants and isolated from rhizomes, exhibited strong plant growth-promoting traits both in vitro and *in planta*, while enhancing photosynthesis, biomass accumulation, and picroside biosynthesis. Additionally, our findings also suggest a strong association of fungal endophytes with the plants through various plant colonizing genes, growth-promoting traits, and metabolic clusters, as evidenced by the pan-genome analysis. Consequently, our investigation provides a first-ever study of the endophytic fungal communities’ diversity of *P. kurrooa* and the potential use of PKRF1 in plant growth promotion and secondary metabolite enhancement.

As a result, our findings lay the groundwork for potentially boosting the productivity of *P. kurrooa* by leveraging the beneficial traits of endophytic fungi. This approach aims to enhance the quality of economically important phytocompounds through targeted modifications of metabolic pathways. It also presents a sustainable method to reduce the negative effects of chemical fertilizers on soil quality and crop productivity in the future. Endophytes with promising PGP activities could be used to develop bioformulations that can be directly applied to cultivated *P. kurrooa* crop, improving plant health and yield. In addition to single-isolate applications, future microbiome restoration strategies may benefit from the development of multi-strain endophytic consortia, incorporating taxa unique to wild plants, to better mimic native microbial diversity and enhance plant performance. However, it is essential to thoroughly examine the interactions between endophytes and other microorganisms, as well as their adaptability within diverse microbial communities. Additionally, extensive validation of their effectiveness under field conditions is necessary to ensure their practical application and success in agricultural settings.

## Supplementary Information

Below is the link to the electronic supplementary material.

Supplementary Material 1

Supplementary Material 2

## Data Availability

The amplicon metagenome sequencing data of Picrorhiza kurrooa generated in this study have been deposited in the NCBI GenBank repository under BioProject accession number PRJNA948802. The library for amplicon sequencing of bacterial 16 S rRNA and fungal ITS regions were prepared using the QIAseq 16 S/ITS Region Panel (Qiagen, Hilden, Germany), which generates mixed reads containing both 16 S and ITS fragments. The raw reads deposited under BioProject PRJNA948802 include both amplicon types, which were separated bioinformatically prior to downstream analysis. The draft genome and raw sequencing reads of the PKRF1 strain are available under BioProject accession number PRJNA1004438.

## References

[1] Gupta S, Chaturvedi P, Kulkarni MG, Van Staden J. A critical review on exploiting the pharmaceutical potential of plant endophytic fungi. Biotechnol Adv. 2020;39:107462. 10.1016/j.biotechadv.2019.107462. 31669137 10.1016/j.biotechadv.2019.107462

[2] Fadiji AE, Babalola OO. Metagenomics methods for the study of plant-associated microbial communities: a review. J Microbiol Methods. 2020;170:105860. 10.1016/j.mimet.2020.105860. 32027927 10.1016/j.mimet.2020.105860

[3] Caruso G, Abdelhamid MT, Kalisz A, Sekara A. Linking endophytic fungi to medicinal plants therapeutic activity. A case study on *Asteraceae*. Agriculture. 2020;10:286. 10.3390/agriculture10070286.

[4] Bielecka M, Pencakowski B, Nicoletti R. Using next-generation sequencing technology to explore genetic pathways in endophytic fungi in the syntheses of plant bioactive metabolites. Agriculture. 2022;12:187. 10.3390/agriculture12020187.

[5] Harman GE, Uphoff N. Symbiotic root-endophytic soil microbes improve crop productivity and provide environmental benefits. Scientifica (Cairo). 2019;2019:9106395. 10.1155/2019/9106395. 31065398 10.1155/2019/9106395 PMC6466867

[6] Grabka R, d’Entremont TW, Adams SJ, Walker AK, Tanney JB, Abbasi PA, et al. Fungal endophytes and their role in agricultural plant protection against pests and pathogens. Plants. 2022;11:384. 10.3390/plants11030384. 35161365 10.3390/plants11030384 PMC8840373

[7] Busby PE, Ridout M, Newcombe G. Fungal endophytes: modifiers of plant disease. Plant Mol Biol. 2016;90:645–55. 10.1007/s11103-015-0412-0. 26646287 10.1007/s11103-015-0412-0

[8] Teimoori-Boghsani Y, Ganjeali A, Cernava T, Müller H, Asili J, Berg G. Endophytic fungi of native *Salvia abrotanoides* plants reveal high taxonomic diversity and unique profiles of secondary metabolites. Front Microbiol. 2020;11:3013. 10.3389/fmicb.2020.580040. 10.3389/fmicb.2019.03013 PMC6978743 32010087

[9] Weese DJ, Heath KD, Dentinger BTM, Lau JA. Long-term nitrogen addition causes the evolution of less-cooperative mutualists. Evolution. 2015;69:631–42. 10.1111/evo.12594. 25565449 10.1111/evo.12594

[10] Tamang A, Swarnkar M, Kumar P, Kumar D, Pandey SS, Hallan V. Endomicrobiome of in vitro and natural plants deciphering the endophytes-associated secondary metabolite biosynthesis in *Picrorhiza kurrooa*, a Himalayan medicinal herb. Microbiol Spectr. 2023;11:e02279–23. 10.1128/spectrum.02279-23. 37811959 10.1128/spectrum.02279-23 PMC10715050

[11] Martin M. Cutadapt removes adapter sequences from high-throughput sequencing reads. EMBnet J. 2011;17:10–2.

[12] Bolyen E, Rideout JR, Dillon MR, Bokulich NA, Abnet CC, Al-Ghalith GA, et al. Reproducible, interactive, scalable and extensible microbiome data science using QIIME 2. Nat Biotechnol. 2019;37:852–7. 10.1038/s41587-019-0209-9. 31341288 10.1038/s41587-019-0209-9 PMC7015180

[13] Callahan BJ, McMurdie PJ, Rosen MJ, Han AW, Johnson AJA, Holmes SP. DADA2: high-resolution sample inference from Illumina amplicon data. Nat Methods. 2016;13:581–3. 10.1038/nmeth.3869. 27214047 10.1038/nmeth.3869 PMC4927377

[14] Abarenkov K, Nilsson RH, Larsson K-H, Taylor AFS, May TW, Frøslev TG, et al. The UNITE database for molecular identification and taxonomic communication of fungi and other eukaryotes: sequences, taxa and classifications reconsidered. Nucleic Acids Res. 2024;52:D791–7. 10.1093/nar/gkad1039. 37953409 10.1093/nar/gkad1039 PMC10767974

[15] Lu Y, Zhou G, Ewald J, Pang Z, Shiri T, Xia J. MicrobiomeAnalyst 2.0: comprehensive statistical, functional and integrative analysis of microbiome data. Nucleic Acids Res. 2023;51:W310–8. 37166960 10.1093/nar/gkad407 PMC10320150

[16] Segata N, Izard J, Waldron L, Gevers D, Miropolsky L, Garrett WS, et al. Metagenomic biomarker discovery and explanation. Genome Biol. 2011;12:1–18. 10.1186/gb-2011-12-6-r60 PMC3218848 21702898

[17] Douglas GM, Maffei VJ, Zaneveld JR, Yurgel SN, Brown JR, Taylor CM, et al. PICRUSt2 for prediction of metagenome functions. Nat Biotechnol. 2020;38:685–8. 10.1038/s41587-020-0548-6. 32483366 10.1038/s41587-020-0548-6 PMC7365738

[18] Pandey SS, Singh S, Babu CSV, Shanker K, Srivastava NK, Shukla AK, et al. Fungal endophytes of *Catharanthus roseus* enhance vindoline content by modulating structural and regulatory genes related to terpenoid indole alkaloid biosynthesis. Sci Rep. 2016;6:26583. 10.1038/srep26583. 27220774 10.1038/srep26583 PMC4879578

[19] Sambrook J, Fritsch EF, Maniatis T. Molecular cloning: a laboratory manual. 2nd edn. Cold Spring Harbor: Cold Spring Harbor Laboratory Press; 1989.

[20] Letunic I, Bork P. Interactive Tree of Life (iTOL) v4: recent updates and new developments. Nucleic Acids Res. 2019;47:W256–9. 10.1093/nar/gkz239. 30931475 10.1093/nar/gkz239 PMC6602468

[21] Nautiyal CS. An efficient microbiological growth medium for screening phosphate solubilizing microorganisms. FEMS Microbiol Lett. 1999;170:265–70. 9919677 10.1111/j.1574-6968.1999.tb13383.x

[22] Adhikari P, Jain R, Sharma A, Pandey A. Plant growth promotion at low temperature by phosphate-solubilizing *Pseudomonas* spp. isolated from high-altitude Himalayan soil. Microb Ecol. 2021;82:677–87. 33512536 10.1007/s00248-021-01702-1

[23] Jain R, Bhardwaj P, Pandey SS, Kumar S. *Arnebia euchroma*, a plant species of cold desert in the Himalayas, harbors beneficial cultivable endophytes in roots and leaves. Front Microbiol. 2021;12:1–16. 10.3389/fmicb.2021.696667 PMC8322769 34335527

[24] Sinha AK, Parli Venkateswaran B, Tripathy SC, Sarkar A, Prabhakaran S. Effects of growth conditions on siderophore producing bacteria and siderophore production from Indian Ocean sector of Southern Ocean. J Basic Microbiol. 2019;59:412–24. 30672596 10.1002/jobm.201800537

[25] Becker Y, Green KA, Scott B, Becker M. Artificial inoculation of *Epichloë festucae* into *Lolium perenne*, and visualisation of endophytic and epiphyllous fungal growth. Bio Protoc. 2018;8:e2990. 10.21769/BioProtoc.2990. 34395789 10.21769/BioProtoc.2990 PMC8328605

[26] Thakur A, Kumar A, Kumar D, Warghat AR, Pandey SS. Physiological and biochemical regulation of *Valeriana jatamansi* Jones under water stress. Plant Physiol Biochem. 2024;208:108304. 10.1016/j.plaphy.2024.108304. 10.1016/j.plaphy.2024.108476 38442628

[27] 27. Lichtenthaler HK, Buschmann C. Chlorophylls and carotenoids: measurement and characterization by UV-VIS spectroscopy. Curr Protoc Food Anal Chem. 2001;1:F4.3.1-8. 10.1002/0471142913.faf0403s01.

[28] Schlie T-P, Dierend W, Köpcke D, Rath T. Detecting low-oxygen stress of stored apples using chlorophyll fluorescence imaging and histogram division. Postharvest Biol Technol. 2022;189:111901. 10.1016/j.postharvbio.2022.111901.

[29] Biswal AK, Pattanayak GK, Pandey SS, Leelavathi S, Reddy VS, Govindjee, et al. Light intensity-dependent modulation of chlorophyll b biosynthesis and photosynthesis by overexpression of chlorophyllide a oxygenase in tobacco. Plant Physiol. 2012;159:433–49. 10.1104/pp.112.195859. 22419827 10.1104/pp.112.195859 PMC3375976

[30] Perez-Ruiz JM, Spínola MC, Kirchsteiger K, Moreno J, Sahrawy M, Cejudo FJ. Rice NTRC is a high-efficiency redox system for chloroplast protection against oxidative damage. Plant Cell. 2006;18:2356–68. 10.1105/tpc.106.041541. 16891402 10.1105/tpc.106.041541 PMC1560923

[31] Dangol S, Chen Y, Hwang BK, Jwa N-S. Iron- and reactive oxygen species-dependent ferroptotic cell death in rice-*Magnaporthe oryzae* interactions. Plant Cell. 2019;31:189–209. 10.1105/tpc.18.00535. 30563847 10.1105/tpc.18.00535 PMC6391706

[32] Kumar D, Kumar R, Singh B, Singh Ahuja P. Comprehensive chemical profiling of *Picrorhiza kurroa* Royle ex Benth using NMR, HPTLC and LC-MS/MS techniques. Comb Chem High Throughput Screen. 2016;19:200–15. 10.2174/1386207319666151203002553. 26777484 10.2174/1386207319666160114092538

[33] Mukhia S, Kumar A, Kumar R. Antioxidant prodigiosin-producing cold-adapted *Janthinobacterium* sp. ERMR3:09 from a glacier moraine: genomic elucidation of cold adaptation and pigment biosynthesis. Gene. 2023;857:147178. 10.1016/j.gene.2023.147178. 36627092 10.1016/j.gene.2023.147178

[34] Wick RR, Judd LM, Gorrie CL, Holt KE. Unicycler: resolving bacterial genome assemblies from short and long sequencing reads. PLoS Comput Biol. 2017;13:e1005595. 10.1371/journal.pcbi.1005595. 28594827 10.1371/journal.pcbi.1005595 PMC5481147

[35] Manni M, Berkeley MR, Seppey M, Simão FA, Zdobnov EM. BUSCO update: novel and streamlined workflows along with broader and deeper phylogenetic coverage for scoring of eukaryotic, prokaryotic, and viral genomes. Mol Biol Evol. 2021;38:4647–54. 10.1093/molbev/msab199. 34320186 10.1093/molbev/msab199 PMC8476166

[36] Gurevich A, Saveliev V, Vyahhi N, Tesler G. QUAST: quality assessment tool for genome assemblies. Bioinformatics. 2013;29:1072–5. 10.1093/bioinformatics/btt086. 23422339 10.1093/bioinformatics/btt086 PMC3624806

[37] Stanke M, Diekhans M, Baertsch R, Haussler D. Using native and syntenically mapped cDNA alignments to improve de novo gene finding. Bioinformatics. 2008;24:637–44. 10.1093/bioinformatics/btn013. 18218656 10.1093/bioinformatics/btn013

[38] Majoros WH, Pertea M, Salzberg SL. TigrScan and GlimmerHMM: two open source ab initio eukaryotic gene-finders. Bioinformatics. 2004;20:2878–9. 10.1093/bioinformatics/bth315. 15145805 10.1093/bioinformatics/bth315

[39] Chan PP, Lowe TM. tRNAscan-SE: Searching for tRNA genes in genomic sequences. In: Kollmar M, editor. Gene Prediction: methods and protocols. New York, NY: Springer; 2019. pp. 1–14. 10.1007/978-1-4939-9173-0_1. 10.1007/978-1-4939-9173-0_1 PMC6768409 31020551

[40] Rawlings ND, Barrett AJ, Thomas PD, Huang X, Bateman A, Finn RD. The MEROPS database of proteolytic enzymes, their substrates and inhibitors in 2017 and a comparison with peptidases in the PANTHER database. Nucleic Acids Res. 2018;46:D624–32. 10.1093/nar/gkx1134. 29145643 10.1093/nar/gkx1134 PMC5753285

[41] Mistry J, Chuguransky S, Williams L, Qureshi M, Salazar GA, Sonnhammer ELL, et al. Pfam: the protein families database in 2021. Nucleic Acids Res. 2021;49:D412–9. 10.1093/nar/gkaa913. 33125078 10.1093/nar/gkaa913 PMC7779014

[42] Consortium TGO, Aleksander SA, Balhoff J, Carbon S, Cherry JM, Drabkin HJ, et al. The gene ontology knowledgebase in 2023. Genetics. 2023;224:iyad031. 10.1093/genetics/iyad031. 36866529 10.1093/genetics/iyad031 PMC10158837

[43] Blum M, Chang H-Y, Chuguransky S, Grego T, Kandasaamy S, Mitchell A, et al. The InterPro protein families and domains database: 20 years on. Nucleic Acids Res. 2021;49:D344–54. 10.1093/nar/gkaa977. 33156333 10.1093/nar/gkaa977 PMC7778928

[44] Galperin MY, Wolf YI, Makarova KS, Vera Alvarez R, Landsman D, Koonin EV. COG database update: focus on microbial diversity, model organisms, and widespread pathogens. Nucleic Acids Res. 2021;49:D274–81. 10.1093/nar/gkaa1018. 33167031 10.1093/nar/gkaa1018 PMC7778934

[45] Kanehisa M, Goto S. KEGG: Kyoto Encyclopedia of Genes and Genomes. Nucleic Acids Res. 2000;28:27–30. 10.1093/nar/28.1.27. 10592173 10.1093/nar/28.1.27 PMC102409

[46] Kim D, Gilchrist CLM, Chun J, Steinegger M. UFCG: database of universal fungal core genes and pipeline for genome-wide phylogenetic analysis of fungi. Nucleic Acids Res. 2023;51:D777–84. 10.1093/nar/gkac894. 36271795 10.1093/nar/gkac894 PMC9825530

[47] Jain C, Rodriguez-R LM, Phillippy AM, Konstantinidis KT, Aluru S. High throughput ANI analysis of 90K prokaryotic genomes reveals clear species boundaries. Nat Commun. 2018;9:5114. 10.1038/s41467-018-07641-9. 30504855 10.1038/s41467-018-07641-9 PMC6269478

[48] Sun J, Lu F, Luo Y, Bie L, Xu L, Wang Y. OrthoVenn3: an integrated platform for exploring and visualizing orthologous data across genomes. Nucleic Acids Res. 2023;51:W397–403. 10.1093/nar/gkad313. 37114999 10.1093/nar/gkad313 PMC10320085

[49] Mendes FK, Vanderpool D, Fulton B, Hahn MW. CAFE 5 models variation in evolutionary rates among gene families. Bioinformatics. 2021;36:5516–8. 10.1093/bioinformatics/btaa1022. 33325502 10.1093/bioinformatics/btaa1022

[50] Mushegian AR, Garey JR, Martin J, Liu LX. Large-scale taxonomic profiling of eukaryotic model organisms: a comparison of orthologous proteins encoded by the human, fly, nematode, and yeast genomes. Genome Res. 1998;8:590–8. 10.1101/gr.8.6.590. 9647634 10.1101/gr.8.6.590

[51] Patz S, Gautam A, Becker M, Ruppel S, Rodríguez-Palenzuela P, Huson DH. PLaBAse: a comprehensive web resource for analyzing the plant growth-promoting potential of plant-associatedbacteria. bioRxiv. 2021:2021.12.21.473721. 10.1101/2021.12.21.473721

[52] Blin K, Shaw S, Steinke K, Villebro R, Ziemert N, Lee SY, et al. antiSMASH 5.0: updates to the secondary metabolite genome mining pipeline. Nucleic Acids Res. 2019;47:W81–7. 10.1093/nar/gkz310. 31032519 10.1093/nar/gkz310 PMC6602434

[53] Reitz ZL. zreitz/multismash: v0.3.0. Zenodo; 2023. 10.5281/zenodo.10152996.

[54] Pandey SS, Jain R, Bhardwaj P, Thakur A, Kumari M, Bhushan S, et al. Plant Probiotics - Endophytes pivotal to plant health. Microbiol Res. 2022;263:127148. 10.1016/j.micres.2022.127148. 35940110 10.1016/j.micres.2022.127148

[55] Bello MGD, Knight R, Gilbert JA, Blaser MJ. Preserving microbial diversity. Science. 2018;362:33–4. 10.1126/science.aau8816. 30287652 10.1126/science.aau8816

[56] Martín-Robles N, Lehmann A, Seco E, Aroca R, Rillig MC, Milla R. Impacts of domestication on the arbuscular mycorrhizal symbiosis of 27 crop species. New Phytol. 2018;218:322–34. 10.1111/nph.14962. 29281758 10.1111/nph.14962

[57] Aly AH, Debbab A, Proksch P. Fungal endophytes: unique plant inhabitants with great promises. Appl Microbiol Biotechnol. 2011;90:1829–45. 10.1007/s00253-011-3270-y. 21523479 10.1007/s00253-011-3270-y

[58] Anguita-Maeso M, Haro C, Montes-Borrego M, De La Fuente L, Navas-Cortés JA, Landa BB. Metabolomic, ionomic and microbial characterization of olive xylem sap reveals differences according to plant age and genotype. Agronomy. 2021;11:1179. 10.3390/agronomy11061179.

[59] Tian L, Wang E, Lin X, Ji L, Chang J, Chen H, et al. Wild rice harbors more root endophytic fungi than cultivated rice in the F1 offspring after crossbreeding. BMC Genomics. 2021;22:1–12. 10.1186/s12864-021-07609-y. 33865333 10.1186/s12864-021-07587-1 PMC8052703

[60] Oono R, Black D, Slessarev E, Sickler B, Strom A, Apigo A. Species diversity of fungal endophytes across a stress gradient for plants. New Phytol. 2020;228:210–25. 10.1111/nph.16704. 32472573 10.1111/nph.16709

[61] Tan X, Zhou Y, Zhou X, Xia X, Wei Y, He L, et al. Diversity and bioactive potential of culturable fungal endophytes of *Dysosma versipellis*; a rare medicinal plant endemic to China. Sci Rep. 2018;8:5929. 10.1038/s41598-018-24313-2. 29651009 10.1038/s41598-018-24313-2 PMC5897559

[62] Vuorela P, Leinonen M, Saikku P, Tammela P, Rauha J-P, Wennberg T, et al. Natural products in the process of finding new drug candidates. Curr Med Chem. 2004;11:1375–89. 10.2174/0929867043365116. 15180572 10.2174/0929867043365116

[63] Hyde KD, Jeewon R, Chen Y-J, Bhunjun CS, Calabon MS, Jiang H-B, et al. The numbers of fungi: Is the descriptive curve flattening? Fungal Divers. 2020;103:219–71. 10.1007/s13225-020-00458-2.

[64] Li Z, Chang P, Gao L, Wang X. The endophytic fungus *Albifimbria verrucaria* from wild grape as an antagonist of *Botrytis cinerea* and other grape pathogens. Phytopathology. 2020;110:843–50. 10.1094/PHYTO-11-19-0415-R. 31799903 10.1094/PHYTO-09-19-0347-R

[65] Wei P-P, Ji J-C, Ma X-J, Li Z-H, Ai H-L, Lei X-X, et al. Three new pyrrole alkaloids from the endophytic fungus *Albifimbria viridis*. Nat Prod Bioprospect. 2022;12:5. 10.1007/s13659-022-00327-2. 35199234 10.1007/s13659-022-00327-2 PMC8866607

[66] Chen J, Tang J, Wei Y, Ma H, Wu J. Diversity of endophytic microbes in roots of wild *Cymbidium goeringii* in Yunnan. Microbiol Res. 2023;266:127254. 10.1016/j.micres.2022.127254. 36371871 10.1016/j.micres.2022.127254

[67] Hou L, Li X, He X, Zuo Y, Zhang D, Zhao L. Effect of dark septate endophytes on plant performance of *Artemisia ordosica* and associated soil microbial functional group abundance under salt stress. Appl Soil Ecol. 2021;165:103998. 10.1016/j.apsoil.2021.103998.

[68] Li X, Zhang X, Xu M, Ye Q, Gao H, He X. Improved tolerance of *Artemisia ordosica* to drought stress via dark septate endophyte (DSE) symbiosis. J Fungi. 2022;8:730. 10.3390/jof8070730. 10.3390/jof8070730 PMC9320036 35887485

[69] Zhang J, Li X, Yin Y, Luo S, Wang D, Zheng H, et al. High-throughput sequencing-based analysis of the composition and diversity of the endophyte community in roots of *Stellera chamaejasme*. Sci Rep. 2024;14:8607. 10.1038/s41598-024-59256-4. 38615120 10.1038/s41598-024-59055-x PMC11016073

[70] Wheeler DL, Dung JKS, Johnson DA. From pathogen to endophyte: an endophytic population of *Verticillium dahliae* evolved from a sympatric pathogenic population. New Phytol. 2019;222:497–510. 10.1111/nph.15567. 30372525 10.1111/nph.15567

[71] Wheeler DL, Johnson DA. *Verticillium isaacii* is a pathogen and endophyte of potato and sunflower in the Columbia Basin of Washington. Plant Dis. 2019;103:3150–3. 10.1094/PDIS-03-19-0592-RE. 31596689 10.1094/PDIS-04-19-0779-RE

[72] Song Z, Sun YJ, Xu S, Li G, Yuan C, Zhou K. Secondary metabolites from the Endophytic fungi *Fusarium decemcellulare* F25 and their antifungal activities. Front Microbiol. 2023;14:1122369. 10.3389/fmicb.2023.1122369. 10.3389/fmicb.2023.1127971 PMC9929939 36819056

[73] Šišić A, Baćanović J, Finckh MR. Endophytic *Fusarium equiseti* stimulates plant growth and reduces root rot disease of pea (*Pisum sativum* L.) caused by *Fusarium avenaceum* and *Peyronellaea pinodella*. Eur J Plant Pathol. 2017;148:271–82. 10.1007/s10658-016-1086-4.

[74] Dong C, Shao Q, Zhang Q, Yao T, Huang J, Liang Z, et al. Preferences for core microbiome composition and function by different definition methods: evidence for the core microbiome of *Eucommia ulmoides* bark. Sci Total Environ. 2021;790:148091. 10.1016/j.scitotenv.2021.148091. 34380268 10.1016/j.scitotenv.2021.148091

[75] Berini F, Caccia S, Franzetti E, Congiu T, Marinelli F, Casartelli M, et al. Effects of *Trichoderma viride* chitinases on the peritrophic matrix of Lepidoptera. Pest Manag Sci. 2016;72:980–9. 10.1002/ps.4078. 26179981 10.1002/ps.4078

[76] Rosmana A, Nasaruddin N, Hendarto H, Akbar A, Agriansyah N. Endophytic association of *Trichoderma asperellum* within *Theobroma cacao* suppresses vascular streak dieback incidence and promotes side graft growth. Mycobiology. 2018;46:154–65. 10.1080/12298093.2018.1468056. 27790069 10.5941/MYCO.2016.44.3.180 PMC5078131

[77] Nascimento Brito V, Lana Alves J, Sírio Araújo K, de Souza Leite T, Borges de Queiroz C, Liparini Pereira O, et al. Endophytic *Trichoderma* species from rubber trees native to the Brazilian Amazon, including four new species. Front Microbiol. 2023;14:1095199. 10.3389/fmicb.2023.1095199. 37143529 10.3389/fmicb.2023.1095199 PMC10151590

[78] Pedrero-Méndez A, Insuasti HC, Neagu T, Illescas M, Rubio MB, Monte E, et al. Why is the correct selection of *Trichoderma* strains important? The case of wheat endophytic strains of *T. harzianum* and *T. simmonsii*. J Fungi. 2021;7:1087. 10.3390/jof7121087. 10.3390/jof7121087 PMC8704890 34947069

[79] Chialva M, De Rose S, Novero M, Lanfranco L, Bonfante P. Plant genotype and seasonality drive fine changes in olive root microbiota. Curr Plant Biol. 2021;28:100219. 10.1016/j.cpb.2021.100219.

[80] Azuddin NF, Mohd MH, Rosely NFN, Mansor A, Zakaria L. Extracellular enzymatic activity of endophytic fungi isolated from spines of rattan palm (*Calamus castaneus* Griff). Malays J Microbiol. 2024;20:1–12. 10.21161/mjm.230023.

[81] Koukol O, Maciá-Vicente JG. Leptodophora gen. nov. (Helotiales, Leotiomycetes) proposed to accommodate selected root-associated members of the genus Cadophora. Czech Mycol. 2022;74:1–15. 10.33585/cmy.74101.

[82] Maciá-Vicente JG, Bai B, Qi R, Ploch S, Breider F, Thines M. Nutrient availability does not affect community assembly in root-associated fungi but determines fungal effects on plant growth. mSystems. 2022;7:e00304–22. 10.1128/msystems.00304-22. 35695510 10.1128/msystems.00304-22 PMC9239174

[83] Kosawang C, Børja I, Herrero M-L, Nagy NE, Nielsen LR, Solheim H, et al. Fungal succession in decomposing ash leaves colonized by the ash dieback pathogen *Hymenoscyphus fraxineus* or its harmless relative *Hymenoscyphus albidus*. Front Microbiol. 2023;14:1154344. 10.3389/fmicb.2023.1154344. 37125194 10.3389/fmicb.2023.1154344 PMC10140306

[84] Massimo NC, Nandi Devan MM, Arendt KR, Wilch MH, Riddle JM, Furr SH, et al. Fungal endophytes in aboveground tissues of desert plants: infrequent in culture, but highly diverse and distinctive symbionts. Microb Ecol. 2015;70:61–76. 10.1007/s00248-014-0562-7. 25645243 10.1007/s00248-014-0563-6 PMC4457668

[85] Al-Hosni K, Shahzad R, Latif Khan A, Muhammad Imran Q, Al Harrasi A, Al Rawahi A, et al. *Preussia* sp. BSL-10 producing nitric oxide, gibberellins, and indole acetic acid and improving rice plant growth. J Plant Interact. 2018;13:112–8. 10.1080/17429145.2018.1432771.

[86] Hardoim PR, Van Overbeek LS, Berg G, Pirttilä AM, Compant S, Campisano A, et al. The hidden world within plants: ecological and evolutionary considerations for defining functioning of microbial endophytes. Microbiol Mol Biol Rev. 2015;79:293–320. 10.1128/MMBR.00050-14. 26136581 10.1128/MMBR.00050-14 PMC4488371

[87] Mohamed AH, Abd El-Megeed FH, Hassanein NM, Youseif SH, Farag PF, Saleh SA, et al. Native rhizospheric and endophytic fungi as sustainable sources of plant growth promoting traits to improve wheat growth under low nitrogen input. J Fungi. 2022;8:94. 10.3390/jof8010094. 10.3390/jof8020094 PMC8875171 35205849

[88] Liu R, Yang L, Zou Y, Wu Q. Root-associated endophytic fungi modulate endogenous auxin and cytokinin levels to improve plant biomass and root morphology of trifoliate orange. Hortic Plant J. 2023;9:463–72. 10.1016/j.hpj.2022.07.003.

[89] Rinu K, Sati P, Pandey A. *Trichoderma gamsii* (NFCCI 2177): a newly isolated endophytic, psychrotolerant, plant growth promoting, and antagonistic fungal strain. J Basic Microbiol. 2014;54:408–17. 10.1002/jobm.201200579. 23564225 10.1002/jobm.201200579

[90] Yadav M, Divyanshu K, Dubey MK, Rai A, Kumar S, Tripathi YN, et al. Plant growth promotion and differential expression of defense genes in chilli pepper against *Colletotrichum truncatum* induced by *Trichoderma asperellum* and *T. harzianum*. BMC Microbiol. 2023;23:54. 10.1186/s12866-023-02789-x. 36864373 10.1186/s12866-023-02789-x PMC9983198

[91] Bononi L, Chiaramonte JB, Pansa CC, Moitinho MA, Melo IS. Phosphorus-solubilizing *Trichoderma* spp. from Amazon soils improve soybean plant growth. Sci Rep. 2020;10:2858. 10.1038/s41598-020-59793-8. 32071331 10.1038/s41598-020-59793-8 PMC7028723

[92] Ali S, Khan MJ, Anjum MM, Khan GR, Ali N. *Trichoderma harzianum* modulates phosphate and micronutrient solubilization in the rhizosphere. Gesunde Pflanzen. 2022;74:853–62. 10.1007/s10343-022-00643-0.

[93] de Oliveira HP, de Melo RO, Cavalcante VS, Monteiro TSA, de Freitas LG, Lambers H, et al. Phosphate fertilizers coated with phosphate-solubilising *Trichoderma harzianum* increase phosphorus uptake and growth of *Zea mays*. Plant Soil. 2024;495:1–18. 10.1007/s11104-024-06818-0.

[94] Syed A, Elgorban AM, Bahkali AH, Eswaramoorthy R, Iqbal RK, Danish S. Metal-tolerant and siderophore producing *Pseudomonas fluorescence* and *Trichoderma* spp. improved the growth, biochemical features and yield attributes of chickpea by lowering Cd uptake. Sci Rep. 2023;13:4471. 10.1038/s41598-023-31686-6. 36934106 10.1038/s41598-023-31330-3 PMC10024765

[95] Chen D, Hou Q, Jia L, Sun K. Combined use of two *Trichoderma* strains to promote growth of pakchoi (*Brassica chinensis* L). Agronomy. 2021;11:726. 10.3390/agronomy11040726.

[96] Liu C, Ye Y, Liu J, Pu Y, Wu C. Iron biofortification of crop food by symbiosis with beneficial microorganisms. J Plant Nutr. 2021;44:2793–810. 10.1080/01904167.2021.1884706.

[97] Carillo P, Woo SL, Comite E, El-Nakhel C, Rouphael Y, Fusco GM, et al. Application of *Trichoderma harzianum*, 6-pentyl-α-pyrone and plant biopolymer formulations modulate plant metabolism and fruit quality of plum tomatoes. Plants. 2020;9:771. 10.3390/plants9060771. 32575500 10.3390/plants9060771 PMC7356659

[98] Rotblat B, Enshell-Seijffers D, Gershoni JM, Schuster S, Avni A. Identification of an essential component of the elicitation active site of the EIX protein elicitor. Plant J. 2002;32:1049–55. 10.1046/j.1365-313X.2002.01490.x. 12492845 10.1046/j.1365-313x.2002.01490.x

[99] Vinale F, Manganiello G, Nigro M, Mazzei P, Piccolo A, Pascale A, et al. A novel fungal metabolite with beneficial properties for agricultural applications. Molecules. 2014;19:9760–72. 10.3390/molecules19079760. 25006784 10.3390/molecules19079760 PMC6271495

[100] Cai F, Yu G, Wang P, Wei Z, Fu L, Shen Q, et al. Harzianolide, a novel plant growth regulator and systemic resistance elicitor from *Trichoderma harzianum*. Plant Physiol Biochem. 2013;73:106–13. 10.1016/j.plaphy.2013.09.004. 24080397 10.1016/j.plaphy.2013.08.011

[101] Ruocco M, Lanzuise S, Lombardi N, Woo SL, Vinale F, Marra R, et al. Multiple roles and effects of a novel *Trichoderma* hydrophobin. Mol Plant-Microbe Interact. 2015;28:167–79. 10.1094/MPMI-07-14-0194-R. 25317667 10.1094/MPMI-07-14-0194-R

[102] Yu W, Mijiti G, Huang Y, Fan H, Wang Y, Liu Z. Functional analysis of eliciting plant response protein Epl1-Tas from *Trichoderma asperellum* ACCC30536. 2018;8:7974. 10.1038/s41598-018-26328-1 10.1038/s41598-018-26328-1 PMC5964103 29789617

[103] Gomes EV, Ulhoa CJ, Cardoza RE, Silva RN, Gutiérrez S. Involvement of *Trichoderma harzianum* Epl-1 protein in the regulation of *Botrytis* virulence-and tomato defense-related genes. Front Plant Sci. 2017;8:880. 10.3389/fpls.2017.00880. 28611802 10.3389/fpls.2017.00880 PMC5446994

[104] Vinale F, Strakowska J, Mazzei P, Piccolo A, Marra R, Lombardi N, et al. Cremenolide, a new antifungal, 10-member lactone from *Trichoderma cremeum* with plant growth promotion activity. Nat Prod Res. 2016;30:2575–81. 10.1080/14786419.2015.1131985. 26728227 10.1080/14786419.2015.1131985

[105] Tandon A, Fatima T, Shukla D, Tripathi P, Srivastava S, Singh PC. Phosphate solubilization by *Trichoderma koningiopsis* (NBRI-PR5) under abiotic stress conditions. J King Saud Univ-Sci. 2020;32:791–8. 10.1016/j.jksus.2019.02.001.

[106] Poveda J. *Trichoderma parareesei* favors the tolerance of rapeseed (*Brassica napus* L.) to salinity and drought due to a chorismate mutase. Agronomy. 2020;10:118. 10.3390/agronomy10010118.

[107] Sun X, Sun M, Chao Y, Wang H, Pan H, Yang Q, et al. Alleviation of lead toxicity and phytostimulation in perennial ryegrass by the Pb-resistant fungus *Trichoderma asperellum* SD-5. Funct Plant Biol. 2020;48:333–41. 10.1071/FP19282. 10.1071/FP20237 33256897

[108] González-Teuber M, Urzúa A, Morales A, Ibáñez C, Bascuñán-Godoy L. Benefits of a root fungal endophyte on physiological processes and growth of the vulnerable legume tree *Prosopis chilensis* (Fabaceae). J Plant Ecol. 2018;12:264–71. 10.1093/jpe/rtx058.

[109] Ruban AV. Nonphotochemical chlorophyll fluorescence quenching: mechanism and effectiveness in protecting plants from photodamage. Plant Physiol. 2016;170:1903–16. 10.1104/pp.15.01935. 26864015 10.1104/pp.15.01935 PMC4825125

[110] Demmig-Adams B, Garab G, Adams W III, Govindjee U. Non-photochemical quenching and energy dissipation in plants, algae and cyanobacteria. Springer; 2014.

[111] González-Teuber M, Contreras RA, Zúñiga GE, Barrera D, Bascuñán-Godoy L. Synergistic association with root endophytic fungi improves morpho-physiological and biochemical responses of *Chenopodium quinoa* to salt stress. Front Ecol Evol. 2022;9:787318. 10.3389/fevo.2021.787318.

[112] Harman GE, Doni F, Khadka RB, Uphoff N. Endophytic strains of *Trichoderma* increase plants’ photosynthetic capability. J Appl Microbiol. 2021;130:529–46. 10.1111/jam.14368. 31271695 10.1111/jam.14368

[113] Maxwell K, Johnson GN. Chlorophyll fluorescence—a practical guide. J Exp Bot. 2000;51:659–68. 10.1093/jexbot/51.345.659. 10938857 10.1093/jxb/51.345.659

[114] Semer J, Navrátil M, Špunda V, Štroch M. Chlorophyll fluorescence parameters to assess utilization of excitation energy in photosystem II independently of changes in leaf absorption. J Photochem Photobiol B. 2019;197:111535. 10.1016/j.jphotobiol.2019.111535. 31319267 10.1016/j.jphotobiol.2019.111535

[115] Passari AK, Upadhyaya K, Singh G, Abdel-Azeem AM, Thankappan S, Uthandi S, et al. Enhancement of disease resistance, growth potential, and photosynthesis in tomato (*Solanum lycopersicum*) by inoculation with an endophytic actinobacterium, *Streptomyces thermocarboxydus* strain BPSAC147. PLoS ONE. 2019;14:e0218014. 10.1371/journal.pone.0218014. 31269087 10.1371/journal.pone.0219014 PMC6608948

[116] Siddiqui ZS, Wei X, Umar M, Abideen Z, Zulfiqar F, Chen J, et al. Scrutinizing the application of saline endophyte to enhance salt tolerance in rice and maize plants. Front Plant Sci. 2022;12:770084. 10.3389/fpls.2021.770084. 35251059 10.3389/fpls.2021.770084 PMC8891170

[117] Kromdijk J, Walter J. Relaxing non-photochemical quenching (NPQ) to improve photosynthesis in crops. Curr Opin Biotechnol. 2023;79:102854. 10.1016/j.copbio.2022.102854. 36455451 10.1016/j.copbio.2022.102854

[118] Horton P. Developments in research on non-photochemical fluorescence quenching: emergence of key ideas, theories and experimental approaches. In: Demmig-Adams B, Garab G, Adams WW, Govindjee III U, editors. Non-photochemical quenching and energy dissipation in plants, algae and cyanobacteria. Springer; 2014. pp. 73–95.

[119] Shahabivand S, Parvaneh A, Aliloo AA. Root endophytic fungus *Piriformospora indica* affected growth, cadmium partitioning and chlorophyll fluorescence of sunflower under cadmium toxicity. Ecotoxicol Environ Saf. 2017;145:496–502. 10.1016/j.ecoenv.2017.07.064. 28783599 10.1016/j.ecoenv.2017.07.064

[120] De Rocchis V, Jammer A, Camehl I, Franken P, Roitsch T. Tomato growth promotion by the fungal endophytes *Serendipita indica* and *Serendipita herbamans* is associated with sucrose de-novo synthesis in roots and differential local and systemic effects on carbohydrate metabolisms and gene expression. J Plant Physiol. 2022;276:153755. 10.1016/j.jplph.2022.153755. 35961165 10.1016/j.jplph.2022.153755

[121] Airin AA, Arafat MI, Begum RA, Islam MR, Seraj ZI. Plant growth-promoting endophytic fungi of the wild halophytic rice *Oryza coarctata*. Ann Microbiol. 2023;73:3. 10.1186/s13213-023-01707-w.

[122] Nassimi Z, Taheri P. Endophytic fungus *Piriformospora indica* induced systemic resistance against rice sheath blight via affecting hydrogen peroxide and antioxidants. Biocontrol Sci Technol. 2017;27:252–67. 10.1080/09583157.2016.1277690.

[123] Sun K, Pan Y-T, Jiang H-J, Xu J-Y, Ma C-Y, Zhou J, et al. Root endophyte-mediated alteration in plant H_2_O_2_ homeostasis regulates symbiosis outcome and reshapes the rhizosphere microbiota. J Exp Bot. 2024;75:3153–70. 10.1093/jxb/erae069. 38394357 10.1093/jxb/erae069

[124] Lima AS, Prieto KR, Santos CS, Paula Valerio H, Garcia-Ochoa EY, Huerta-Robles A, et al. In-vivo electrochemical monitoring of H_2_O_2_ production induced by root-inoculated endophytic bacteria in *Agave tequilana* leaves. Biosens Bioelectron. 2018;99:108–14. 10.1016/j.bios.2017.07.048. 28746900 10.1016/j.bios.2017.07.039

[125] Wang Y, Dai C-C, Zhao Y-W, Peng Y. Fungal endophyte-induced volatile oil accumulation in *Atractylodes lancea* plantlets is mediated by nitric oxide, salicylic acid and hydrogen peroxide. Process Biochem. 2011;46:730–5. 10.1016/j.procbio.2010.11.020.

[126] Tulkova Е, Kabashnikova L. Malondialdehyde content in the leaves of small-leaved linden (*Tilia cordata*) and Norway maple (*Acer platanoides*) under the influence of volatile organic compounds. Plant Biosyst. 2022;156:619–27. 10.1080/11263504.2021.1897701.

[127] Pan L, Cui S, Dinkins RD, Jiang Y. Plant growth, ion accumulation, and antioxidant enzymes of endophyte-infected and endophyte-free tall fescue to salinity stress. Acta Physiol Plant. 2021;43:95. 10.1007/s11738-021-03268-4.

[128] Li X, Bu N, Li Y, Ma L, Xin S, Zhang L. Growth, photosynthesis and antioxidant responses of endophyte infected and non-infected rice under lead stress conditions. J Hazard Mater. 2012;213–214:55–61. 10.1016/j.jhazmat.2012.01.066. 22356744 10.1016/j.jhazmat.2012.01.052

[129] Sharma N, Kumar V, Chauhan RS, Sood H. Modulation of picroside-i biosynthesis in grown elicited shoots of *Picrorhiza kurroa* in vitro. J Plant Growth Regul. 2016;35:965–73. 10.1007/s00344-016-9598-x.

[130] Kuniyal CP, Bisht R. Productivity and picroside contents of *Picrorhiza kurroa* Royle ex Benth. cultivated at multi-locations in Uttarakhand, India. Ind Crops Prod. 2024;207:117747. 10.1016/j.indcrop.2023.117747.

[131] Ming Q, Su C, Zheng C, Jia M, Zhang Q, Zhang H, et al. Elicitors from the endophytic fungus *Trichoderma atroviride* promote *Salvia miltiorrhiza* hairy root growth and tanshinone biosynthesis. J Exp Bot. 2013;64:5687–94. 10.1093/jxb/ert342. 24127517 10.1093/jxb/ert342

[132] Kushwaha RK, Singh S, Pandey SS, Rao DKV, Nagegowda DA, Kalra A, et al. Compatibility of inherent fungal endophytes of *Withania somnifera* with *Trichoderma viride* and its impact on plant growth and withanolide content. J Plant Growth Regul. 2019;38:1228–42. 10.1007/s00344-019-09928-7.

[133] Kharb A, Chauhan RS. Complexity of gene paralogues resolved in biosynthetic pathway of hepatoprotective iridoid glycosides in a medicinal herb, *Picrorhiza kurroa* through differential NGS transcriptomes. Mol Genet Genomics. 2021;296:863–76. 10.1007/s00438-021-01791-0. 33899140 10.1007/s00438-021-01787-w

[134] Kumar V, Chauhan RS, Tandon C. Biosynthesis and therapeutic implications of iridoid glycosides from *Picrorhiza* genus: the road ahead. J Plant Biochem Biotechnol. 2017;26:1–13. 10.1007/s13562-016-0366-6.

[135] Shitiz K, Pandit S, Chauhan RS, Sood H. Picrosides content in the rhizomes of *Picrorhiza kurroa* Royle ex Benth. traded for herbal drugs in the markets of North India. Int J Med Arom Plants. 2013;3:2249–4340.

[136] Langer KM, Jones CR, Jaworski EA, Rushing GV, Kim JY, Clark DG, et al. PhDAHP1 is required for floral volatile benzenoid/phenylpropanoid biosynthesis in *Petunia× hybrida* cv ‘Mitchell Diploid’. Phytochemistry. 2014;103:22–31. 10.1016/j.phytochem.2014.03.025. 24815009 10.1016/j.phytochem.2014.04.004

[137] Collu G, Unver N, Peltenburg-Looman AMG, van der Heijden R, Verpoorte R, Memelink J. Geraniol 10-hydroxylase1, a cytochrome P450 enzyme involved in terpenoid indole alkaloid biosynthesis. FEBS Lett. 2001;508:215–20. 10.1016/S0014-5793(01)03045-9. 11718718 10.1016/s0014-5793(01)03045-9

[138] Vashisht I, Pal T, Sood H, Chauhan RS. Comparative transcriptome analysis in different tissues of a medicinal herb, *Picrorhiza kurroa* pinpoints transcription factors regulating picrosides biosynthesis. Mol Biol Rep. 2016;43:1395–409. 10.1007/s11033-016-4070-3. 27633652 10.1007/s11033-016-4073-0

[139] Xu C, Wei H, Movahedi A, Sun W, Ma X, Li D, et al. Evaluation, characterization, expression profiling, and functional analysis of DXS and DXR genes of *Populus trichocarpa*. Plant Physiol Biochem. 2019;142:94–105. 10.1016/j.plaphy.2019.06.032. 31279136 10.1016/j.plaphy.2019.05.034

[140] Kim DS, Hwang BK. An important role of the pepper phenylalanine ammonia-lyase gene (*PAL1*) in salicylic acid-dependent signalling of the defence response to microbial pathogens. J Exp Bot. 2014;65:2295–306. 10.1093/jxb/eru109. 24642849 10.1093/jxb/eru109 PMC4036500

[141] Koopmann E, Logemann E, Hahlbrock K. Regulation and functional expression of cinnamate 4-hydroxylase from parsley. Plant Physiol. 1999;119:49–56. 10.1104/pp.119.1.49. 9880345 10.1104/pp.119.1.49 PMC32241

[142] Kumar V, Bansal A, Chauhan RS. Modular design of picroside-II biosynthesis deciphered through NGS transcriptomes and metabolic intermediates analysis in naturally variant chemotypes of a medicinal herb, *Picrorhiza kurroa*. Front Plant Sci. 2017;8:1–17. 10.3389/fpls.2017.00564. 28443130 10.3389/fpls.2017.00564 PMC5387076

[143] Shitiz K, Sharma N, Pal T, Sood H, Chauhan RS. NGS transcriptomes and enzyme inhibitors unravel complexity of picrosides biosynthesis in *Picrorhiza kurroa* Royle ex. Benth. PLoS ONE. 2015;10:e0144546. 10.1371/journal.pone.0144546. 26658062 10.1371/journal.pone.0144546 PMC4687646

[144] Shoresh M, Harman GE, Mastouri F. Induced systemic resistance and plant responses to fungal biocontrol agents. Annu Rev Phytopathol. 2010;48:21–43. 10.1146/annurev-phyto-073009-114450. 20192757 10.1146/annurev-phyto-073009-114450

[145] Jeyasri R, Muthuramalingam P, Karthick K, Shin H, Choi SH, Ramesh M. Methyl jasmonate and salicylic acid as powerful elicitors for enhancing the production of secondary metabolites in medicinal plants: an updated review. Plant Cell Tissue Organ Cult. 2023;153:447–58. 10.1007/s11240-023-02478-7. 37197003 10.1007/s11240-023-02485-8 PMC10026785

[146] Bhat WW, Dhar N, Razdan S, Rana S, Mehra R, Nargotra A, et al. Molecular characterization of UGT94F2 and UGT86C4, two glycosyltransferases from *Picrorhiza kurrooa*: comparative structural insight and evaluation of substrate recognition. PLoS ONE. 2013;8:e73804. 10.1371/journal.pone.0073804. 24066073 10.1371/journal.pone.0073804 PMC3774767

[147] Brotman Y, Landau U, Cuadros-Inostroza Á, Takayuki T, Fernie AR, Chet I, et al. *Trichoderma*-plant root colonization: escaping early plant defense responses and activation of the antioxidant machinery for saline stress tolerance. PLoS Pathog. 2013;9:e1003221. 10.1371/journal.ppat.1003221. 23516362 10.1371/journal.ppat.1003221 PMC3597500

[148] Bhat WW, Razdan S, Rana S, Dhar N, Wani TA, Qazi P, et al. A phenylalanine ammonia-lyase ortholog (*PkPAL1*) from *Picrorhiza kurrooa* Royle ex. Benth: molecular cloning, promoter analysis and response to biotic and abiotic elicitors. Gene. 2014;547:245–56. 10.1016/j.gene.2014.06.042. 24979341 10.1016/j.gene.2014.06.046

[149] Li Z, Xiong K, Wen W, Li L, Xu D. Functional endophytes regulating plant secondary metabolism: current status, prospects and applications. Int J Mol Sci. 2023;24(2):1153. 10.3390/ijms24021153. 36674663 10.3390/ijms24021153 PMC9867233

[150] Gupta S, Chaturvedi P. Enhancing secondary metabolite production in medicinal plants using endophytic elicitors: a case study of *Centella asiatica* (Apiaceae) and asiaticoside. In: Hodkinson TR, Doohan FM, Saunders MJ, Murphy BR, editors. Endophytes for a Growing World. Cambridge: Cambridge University Press; 2019. pp. 310–27. 10.1017/9781108607667.015.

[151] Yuan J, Zhang W, Sun K, Tang MJ, Chen PX, Li X, Dai CC. Comparative transcriptomics and proteomics of *Atractylodes lancea* in response to endophytic fungus *Gilmaniella* sp. AL12 reveals regulation in plant metabolism. Front Microbiol. 2019;10:1208. 10.3389/fmicb.2019.01208. 31191508 10.3389/fmicb.2019.01208 PMC6546907

[152] Aleynova OA, Suprun AR, Nityagovsky NN, Dubrovina AS, Kiselev KV. The influence of the grapevine bacterial and fungal endophytes on biomass accumulation and stilbene production by the in vitro cultivated cells of *Vitis amurensis* Rupr. Plants. 2021;10:1276. 10.3390/plants10071276. 34201750 10.3390/plants10071276 PMC8309151

[153] Cai F, Druzhinina IS. honor of John Bissett: authoritative guidelines on molecular identification of *Trichoderma*. Fungal Divers. 2021;107:1–69. 10.1007/s13225-020-00464-4.

[154] de Albuquerque NRM, Haag KL. Using average nucleotide identity (ANI) to evaluate microsporidia species boundaries based on their genetic relatedness. J Eukaryot Microbiol. 2023;70:e12944. 10.1111/jeu.12944. 36039868 10.1111/jeu.12944

[155] Wibberg D, Stadler M, Lambert C, Bunk B, Spröer C, Rückert C, et al. High quality genome sequences of thirteen Hypoxylaceae (Ascomycota) strengthen the phylogenetic family backbone and enable the discovery of new taxa. Fungal Divers. 2021;106:7–28. 10.1007/s13225-020-00447-5.

[156] Li E, Zhang H, Jiang H, Pieterse CMJ, Jousset A, Bakker PAHM. Experimental-evolution-driven identification of *Arabidopsis* rhizosphere competence genes in *Pseudomonas protegens*. mBio. 2021;12:e00927–21. 10.1128/mBio.00927-21. 34101491 10.1128/mBio.00927-21 PMC8262913

[157] Marek-Kozaczuk M, Skorupska A. Production of B-group vitamins by plant growth-promoting *Pseudomonas fluorescens* strain 267 and the importance of vitamins in the colonization and nodulation of red clover. Biol Fertil Soils. 2001;33:146–51. 10.1007/s003740000304.

[158] Taylor JT, Harting R, Shalaby S, Kenerley CM, Braus GH, Horwitz BA. Adhesion as a focus in trichoderma-root interactions. J Fungi. 2022;8:372. 10.3390/jof8040372. 10.3390/jof8040372 PMC9026816 35448603

[159] Schmoll M. Regulation of plant cell wall degradation by light in *Trichoderma*. Fungal Biol Biotechnol. 2018;5:10. 10.1186/s40694-018-0052-7. 29713489 10.1186/s40694-018-0052-7 PMC5913809

[160] Larriba E, Jaime MDLA, Nislow C, Martín-Nieto J, Lopez-Llorca LV. Endophytic colonization of barley (*Hordeum vulgare*) roots by the nematophagous fungus *Pochonia chlamydosporia* reveals plant growth promotion and a general defense and stress transcriptomic response. J Plant Res. 2015;128:665–78. 10.1007/s10265-015-0731-x. 25982739 10.1007/s10265-015-0731-x

[161] Nicolás C, Hermosa R, Rubio B, Mukherjee PK, Monte E. Trichoderma genes in plants for stress tolerance-status and prospects. Plant Sci. 2014;228:71–8. 10.1016/j.plantsci.2014.03.006. 25438787 10.1016/j.plantsci.2014.03.005

[162] Tseng YH, Rouina H, Groten K, Rajani P, Furch ACU, Reichelt M, et al. An endophytic trichoderma strain promotes growth of its hosts and defends against pathogen attack. Front Plant Sci. 2020;11:573672. 10.3389/fpls.2020.573672. 10.3389/fpls.2020.573670 PMC7793846 33424876

[163] Kidwai MK, Malik A, Dhull SB, Rose PK, Garg VK. Bioremediation potential of *Trichoderma* species for metal(loid)s. In: Saxena G, Kumar V, Shah MP, editors. Bioremediation of Toxic Metal(loid)s. CRC; 2022. pp. 137–52.

[164] Kumar V, Dwivedi SK. Bioremediation mechanism and potential of copper by actively growing fungus *Trichoderma lixii* CR700 isolated from electroplating wastewater. J Environ Manage. 2021;277:111370. 10.1016/j.jenvman.2020.111370. 32979751 10.1016/j.jenvman.2020.111370

[165] Markham P, Robson GD, Bainbridge BW, Trinci APJ. Choline: its role in the growth of filamentous fungi and the regulation of mycelial morphology. FEMS Microbiol Rev. 1993;10:287–300. 10.1111/j.1574-6976.1993.tb00025.x. 8318261 10.1111/j.1574-6968.1993.tb05872.x

[166] Sakamoto A, Murata N. The use of bacterial choline oxidase, a glycinebetaine-synthesizing enzyme, to create stress-resistant transgenic plants. Plant Physiol. 2001;125:180–8. 10.1104/pp.125.1.180. 11154327 10.1104/pp.125.1.180 PMC1539357

[167] Geremia F, Paim I, da Silva Camargo M, Schrank A, Sbaraini N. Secondary metabolite gene clusters from the phytopathogenic fungus *Gaeumannomyces tritici*. J Plant Pathol. 2024;106:657–69. 10.1007/s42161-024-01610-6.

[168] Han W, Wu Z, Zhong Z, Williams J, Jacobsen SE, Sun Z, et al. Assessing the biosynthetic inventory of the biocontrol fungus *Trichoderma afroharzianum* T22. J Agric Food Chem. 2023;71:11502–19. 10.1021/acs.jafc.3c03240. 37471583 10.1021/acs.jafc.3c03240

[169] Shah AA, Shah AN, Bilal Tahir M, Abbas A, Javad S, Ali S, et al. Harzianopyridone supplementation reduced chromium uptake and enhanced activity of antioxidant enzymes in Vigna radiata seedlings exposed to chromium toxicity. Front Plant Sci. 2022;13:881561. 10.3389/fpls.2022.881561. 35860543 10.3389/fpls.2022.881561 PMC9290437

[170] Zhang Z, Tang S, Liu X, Ren X, Wang S, Gao Z. The effects of *Trichoderma viride* T23 on rhizosphere soil microbial communities and the metabolomics of muskmelon under continuous cropping. Agronomy. 2023;13:1092. 10.3390/agronomy13041092.

[171] Martirena-Ramírez A, Serrano-Gamboa JG, Pérez-Llano Y, Zenteno-Alegría CO, Iza-Arteaga ML, del Rayo Sánchez-Carbente M, et al. *Aspergillus brasiliensis* E_15.1: a novel thermophilic endophyte from a volcanic crater unveiled through comprehensive genome-wide, phenotypic analysis, and plant growth-promoting trails. J Fungi. 2024;10:517. 10.3390/jof10070517. 10.3390/jof10080517 PMC11355416 39194843

[172] Landeis A, Schmidt-Heydt M. Sequencing and analysis of the entire genome of the mycoparasitic fungus *Trichoderma afroharzianum*. Microbiol Resour Announc. 2021;10:e00304–21. 10.1128/MRA.00304-21. 33858929 10.1128/MRA.00211-21 PMC8050971

[173] Pang G, Sun T, Yu Z, Yuan T, Liu W, Zhu H, et al. Azaphilones biosynthesis complements the defence mechanism of *Trichoderma guizhouense* against oxidative stress. Environ Microbiol. 2020;22:4808–24. 10.1111/1462-2920.15225. 32985773 10.1111/1462-2920.15246

[174] Yang L-J, Peng X-Y, Zhang Y-H, Liu Z-Q, Li X, Gu Y-C, et al. Antimicrobial and antioxidant polyketides from a deep-sea-derived fungus *Aspergillus versicolor* SH0105. Mar Drugs. 2020;18:636. 10.3390/md18120636. 33322355 10.3390/md18120636 PMC7764742

[175] Rush TA, Shrestha HK, Gopalakrishnan Meena M, Spangler MK, Ellis JC, Labbé JL, et al. Bioprospecting *Trichoderma*: a systematic roadmap to screen genomes and natural products for biocontrol applications. Front Fungal Biol. 2021;2:716511. 10.3389/ffunb.2021.716511. 37744103 10.3389/ffunb.2021.716511 PMC10512312

[176] Abdou R, Dawoud M. Cytotoxic metabolites of *Alternaria* alternata, an endophyte of the medicinal plant *Bidens bipinnata*. Int J Pharm Pharm Sci. 2020;12:42–8. 10.22159/ijpps.2020v12i1.35881.

[177] Murai K, Lauterbach L, Teramoto K, Quan Z, Barra L, Yamamoto T, et al. An unusual skeletal rearrangement in the biosynthesis of the sesquiterpene trichobrasilenol from *Trichoderma*. Angew Chem Int Ed. 2019;58:15046–50. 10.1002/anie.201908040. 10.1002/anie.201907964 PMC7687074 31418991

[178] Cong Z, Yin Q, Tian K, Mukoma NJ, Ouyang L, Hsiang T, et al. Genome mining of fungal unique trichodiene synthase-like sesquiterpene synthases. J Fungi. 2024;10:350. 10.3390/jof10050350. 10.3390/jof10050350 PMC11122449 38786705

[179] Gabriel HB, Silva MF, Kimura EA, Wunderlich G, Katzin AM, Azevedo MF. Squalestatin is an inhibitor of carotenoid biosynthesis in *Plasmodium falciparum*. Antimicrob Agents Chemother. 2015;59:3180–8. 10.1128/AAC.04570-14. 25779575 10.1128/AAC.04500-14 PMC4432196

