# Supplementary material for: Unravelling the fungal endomicrobiome of Picrorhiza kurrooa for increasing in-planta picroside biosynthesis using endophytic Trichoderma harzianum PKRF1
Source: Environ Microbiome. 2026 May 11;21:85. doi: 10.1186/s40793-026-00909-4 (PMC13330369; doi:10.1186/s40793-026-00909-4)
Supplement: Supplementary file 1 — Supplementary Material 1 [file 40793_2026_909_MOESM1_ESM.docx]

**Supplementary Information**

**Unravelling the fungal endomicrobiome of *Picrorhiza kurrooa* for increasing *in-planta* picroside biosynthesis using endophytic *Trichoderma harzianum* PKRF1**

**Anish Tamang^1,4^, Anil Kumar^1,4^, Ankita Thakur^2,4^, Rakshak Kumar^1,4,5^, Dinesh Kumar^3,4^, Vipin Hallan^1,4,#^, Shiv Shanker Pandey^2,4,#^**

^1^Biotechnology Division, CSIR-Institute of Himalayan Bioresource Technology (IHBT), Palampur- 176061 (H.P), India.

^2^Fermentation and Phytofarming Technology Division, CSIR-Institute of Himalayan Bioresource Technology (IHBT), Palampur- 176061 (H.P), India.

^3^Chemical Technology Division, CSIR-Institute of Himalayan Bioresource Technology (IHBT), Palampur- 176061 (H.P), India.

^4^Academy of Scientific and Innovative Research (AcSIR), Ghaziabad- 201002, India

Present Address:

^5^Department of Molecular Biology & Bioinformatics, Tripura University (A Central University), Agartala, Tripura, India

**# Address correspondence to: Shiv Shanker Pandey, E-mail: shivpandey@ihbt.res.in**

**ORCID ID: 0000-0001-9730-2547**

**Vipin Hallan, E-mail: hallan@ihbt.res.in**

**ORCID ID: 0000-0002-5189-7327**

**Number of pages: 21**

**Number of figures: 7**

**Number of tables: 10**

**
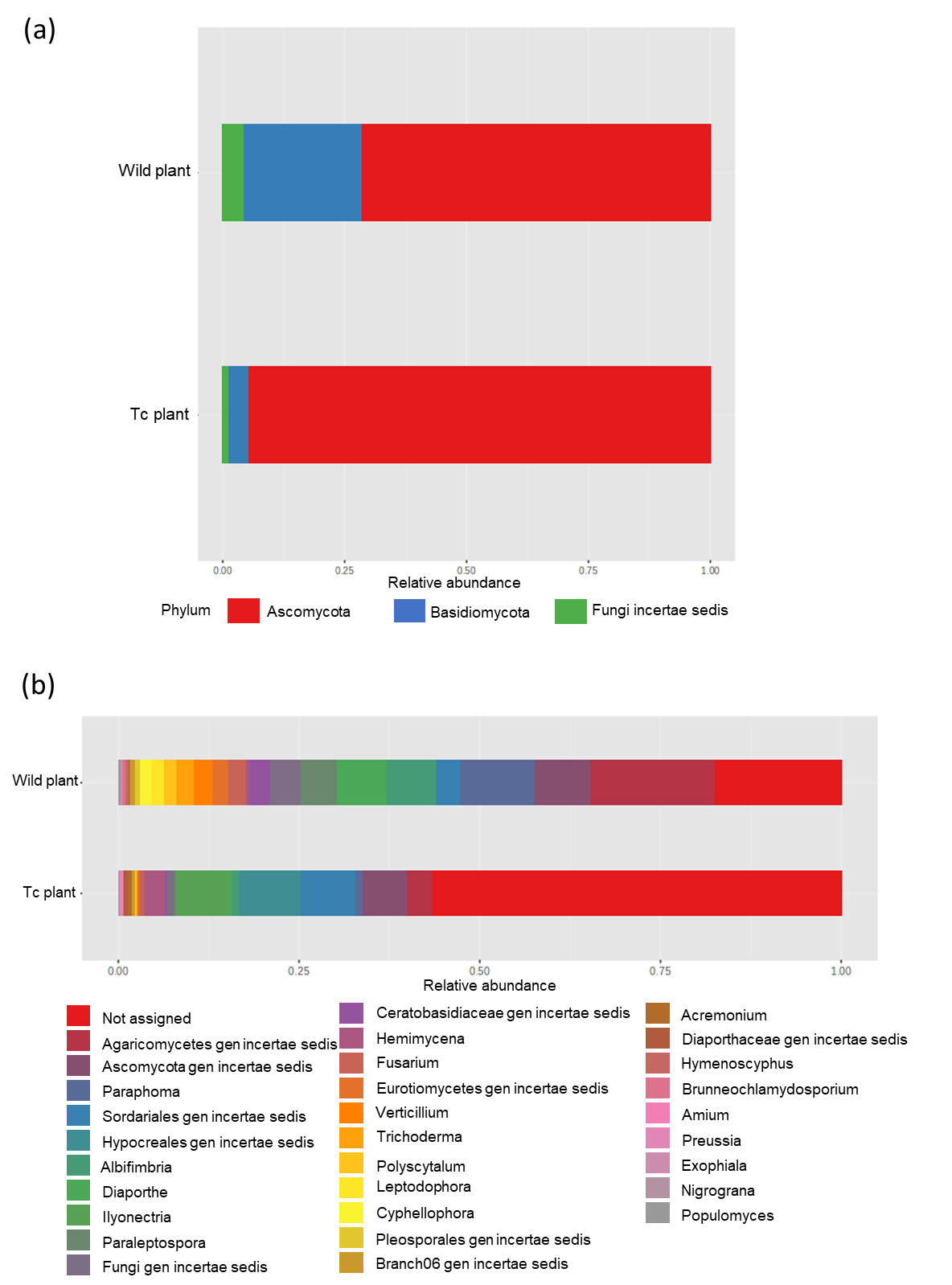
**

**Fig.** **S1** Relative abundance of fungal taxa in Wt and Tc *P. kurrooa* at **(a)** phylum and **(b)** genus level. ‘Not assigned’ indicates sequences that could not be classified to any fungal taxonomic group at the defined confidence threshold, whereas ‘Fungi_gen_incertae_sedis’ represents sequences assigned to the fungal kingdom but with unresolved genus-level classification.

**
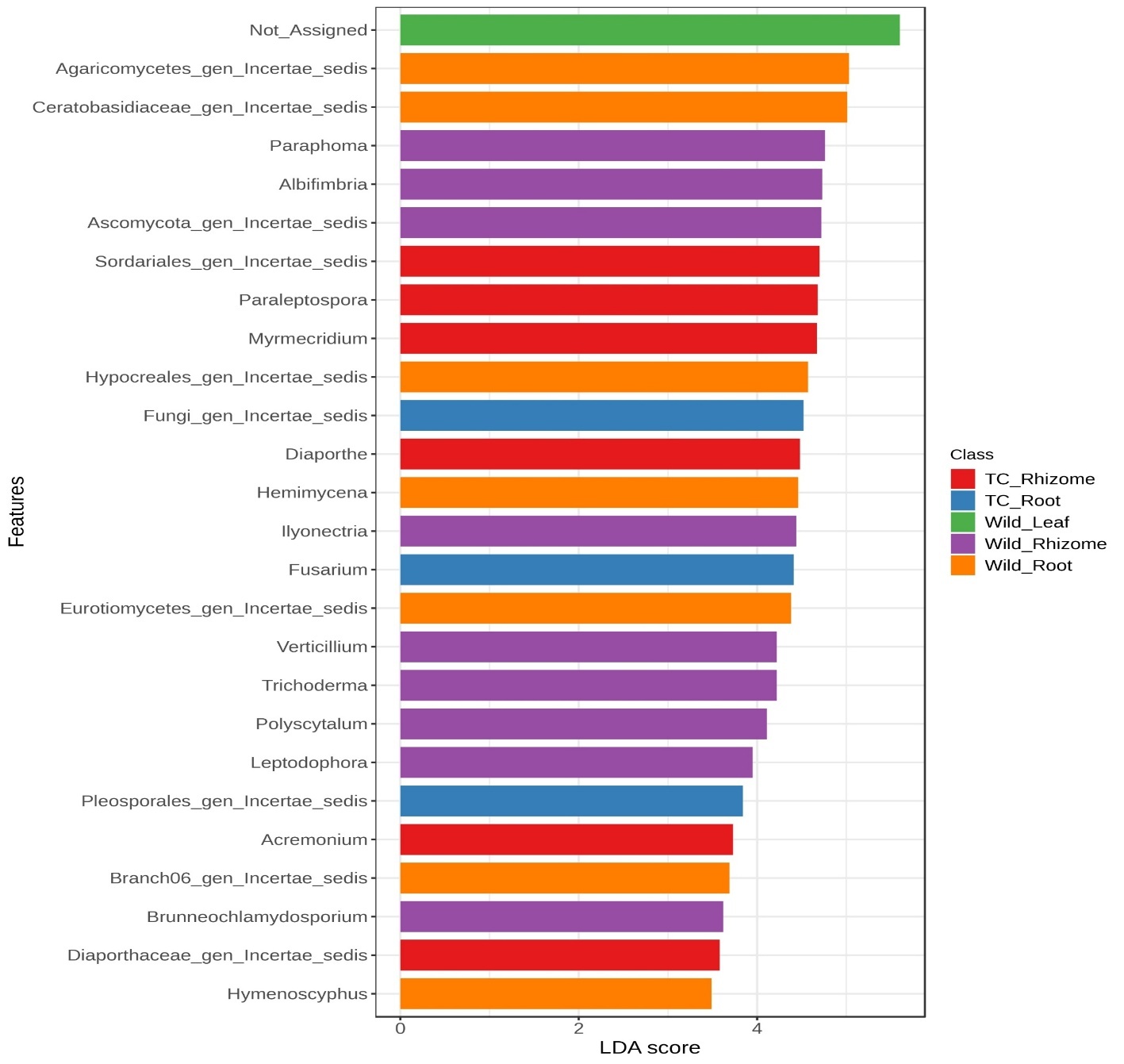
**

**Fig. S2** Differentially abundant fungal taxa found in *P. kurrooa*. Linear discriminant analysis (LDA) effect size (LEfSe) comparison of relative abundance. Horizontal bars represent the effect size for each taxon. LDA score cut-off 2 was used. ‘Not assigned’ indicates sequences that could not be classified to any fungal taxonomic group at the defined confidence threshold, whereas ‘Fungi_gen_incertae_sedis’ represents sequences assigned to the fungal kingdom but with unresolved genus-level classification.

**
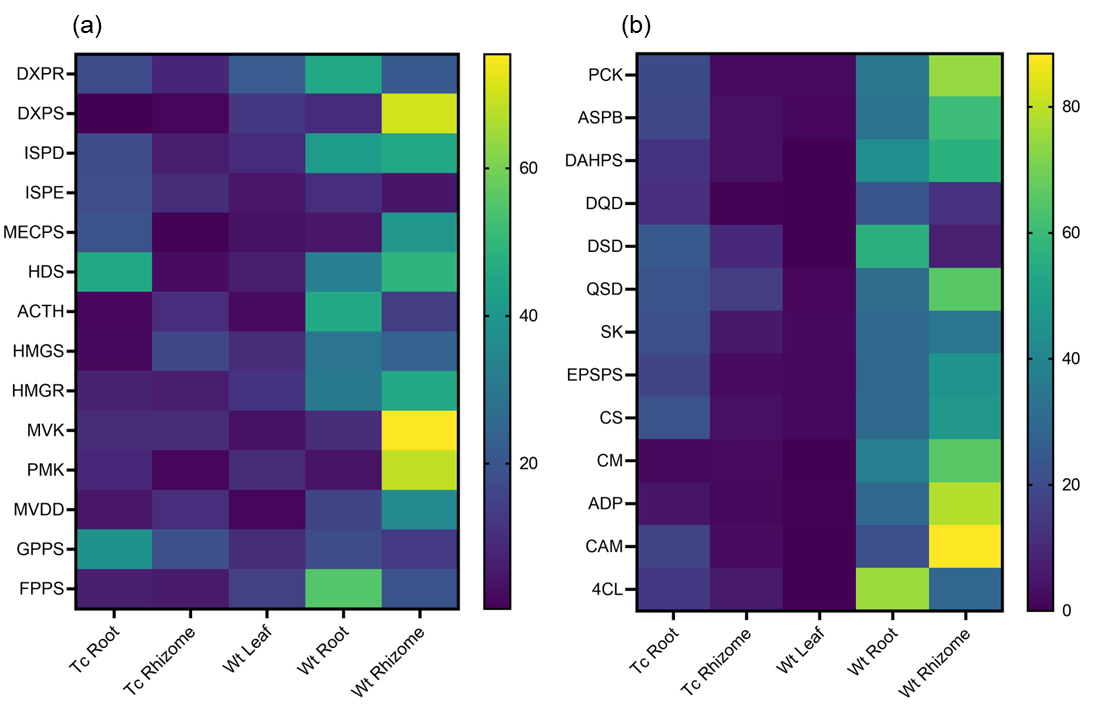
**

**Fig. S3** Predictive functional analysis of endophytic community using PICRUSt2, representing different anatomical parts of Wt and Tc plants. Heat map representing the predicted genes that encode key enzymes involved in picroside biosynthesis; **(a)** Terpenoid backbone (MVA and MEP) pathway and **(b)** Shikimate and Phenylpropanoid Pathway. DXPR, 1-deoxy-D-xylulose 5-phosphate reductase; DXPS, 1-deoxy-D-xylulose 5-phosphate synthase; ISPD, 2-C- methylerythritol 4-phosphate cytidyl transferase; ISPE, 4-(cytidine-50-diphospho)−2-Cmethylerythritol kinase; MECPS, 2-C-methylerythritol-2,4- cyclophosphate synthase; HDS, 1-hydroxy-2-methyl- 2-(E)-butenyl 4-diphosphate synthase; ACTH, Acetoacetyl-CoA thiolase; HMGS, Hydroxymethyl glutaryl CoA synthase; HMGR, Hydroxymethyl glutaryl CoA reductase; MVK, Mevalonate kinase; PMK, Phosphomevalonate kinase; MVDD, Mevalonate diphosphate decarboxylase; GPPS, Geraniol diphosphate synthase; FPPS, farnesyl-diphosphate synthase; PCK, phosphoenolpyruvate carboxykinase; ASPB, aspartate aminotransferase; DAHPS, 3-Deoxy-D-arabinoheptulosonate 7- phosphate synthase; DQD, Dehydroquinate dehydratase; DSD, Dehydroshikimate dehydratase; QSD, Quinate/shikimate dehydrogenase; SK, Shikimate kinase; EPSPS, 5- enolpyruvylshikimic acid-3-phosphate synthase; CS, Chorismate synthase; CM, Chorismate mutase; ADP, Arogenate/prephenate dehydratase; CAM, Caffeic acid-3- o-methyltransferase; 4Cl, 4-coumarate-CoA ligase; TAT, Tyrosine aminotransferase; PAL, Phenylalanine ammonia lyase; C4H, Cinnamate 4 hydroxylase.

**
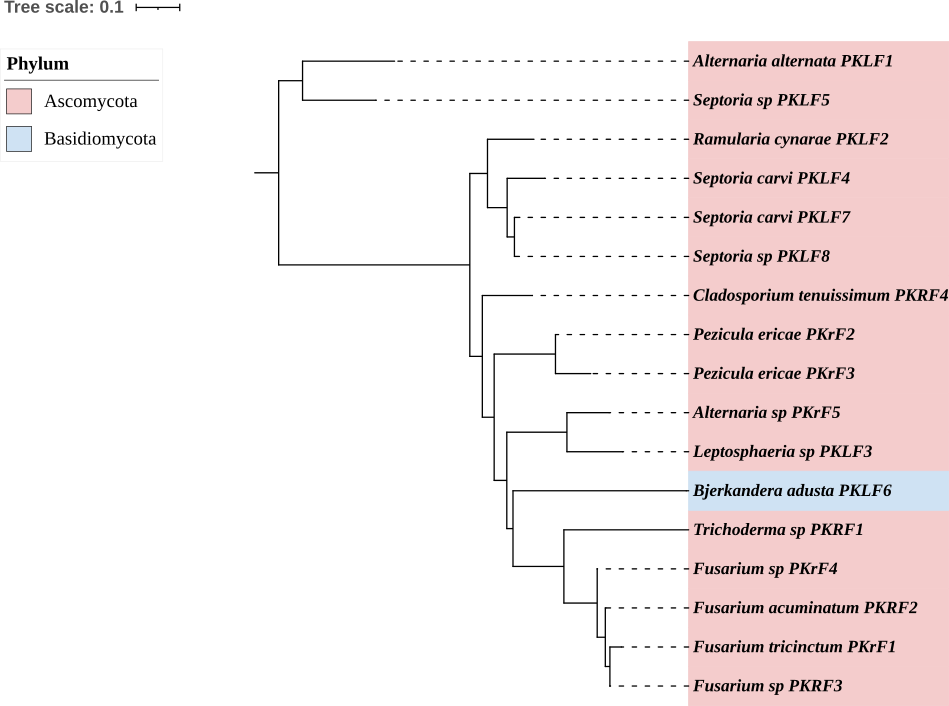
**

**Fig. S4** Phylogenetic relationship among isolated fungal endophytes from *Picrorhiza kurrooa*. ITS rDNA sequences of isolated fungal endophytes were aligned using the MUSCLE algorithm in MEGA X, and the phylogenetic tree was constructed using the Neighbor-Joining method with bootstrap analysis (1000 replicates). Taxa are color-coded by phylum: Ascomycota (pink) and Basidiomycota (blue). The isolate *Bjerkandera adusta* PKLF6 represents the only Basidiomycete, while the remaining isolates belong to Ascomycota, including genera such as *Fusarium*, *Trichoderma*, *Alternaria*, *Septoria*, *Ramularia*, and *Pezicula*. As no defined outgroup was included, the tree is presented as unrooted, and taxa from different phyla may appear interspersed depending on sequence similarity.


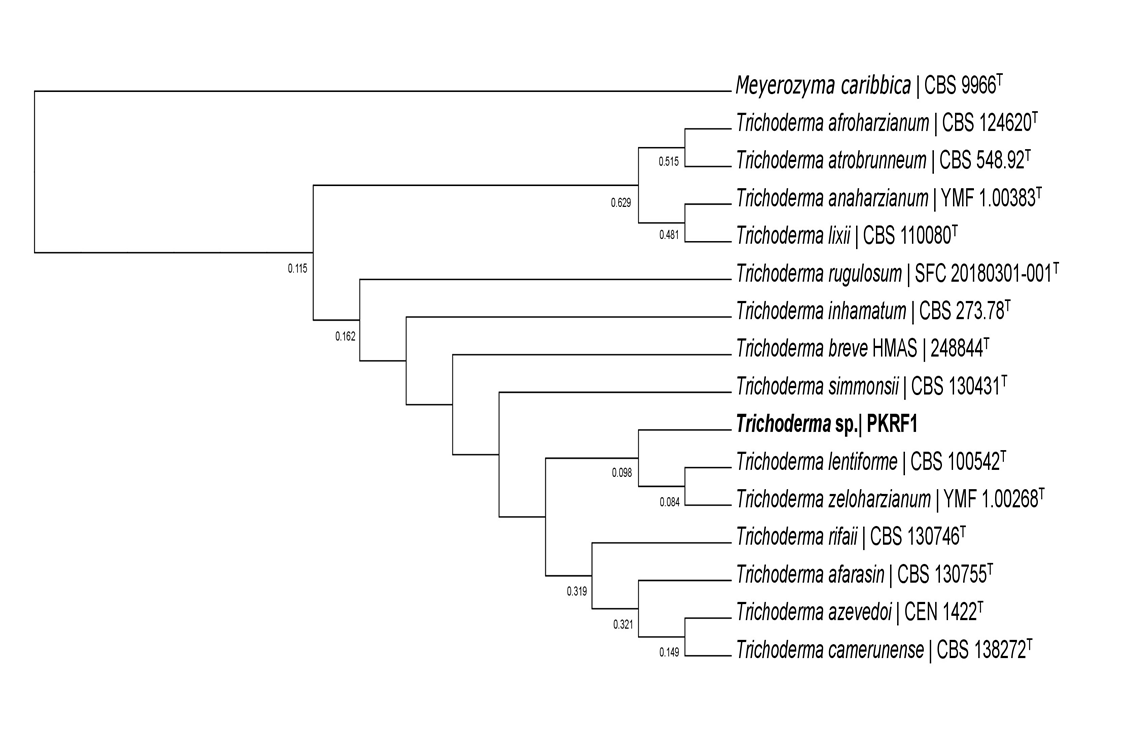


**Fig. S5** Phylogenetic tree based on ITS sequence analysis showing the relationship of *Trichoderma* sp. PKRF1 with closely related *Trichoderma* species and the outgroup *Meyerozyma caribbica* (CBS 9966ᵀ). The tree was constructed using the Neighbor-Joining method with evolutionary distances computed using the Kimura 2-parameter model. Bootstrap values are shown at the nodes (only values ≥0.05 are displayed). The strain *Trichoderma* sp. PKRF1 is highlighted in bold. GenBank accession or strain numbers are indicated after species names. The tree reveals the distinct phylogenetic placement of PKRF1 in relation to other known *Trichoderma* taxa.

**
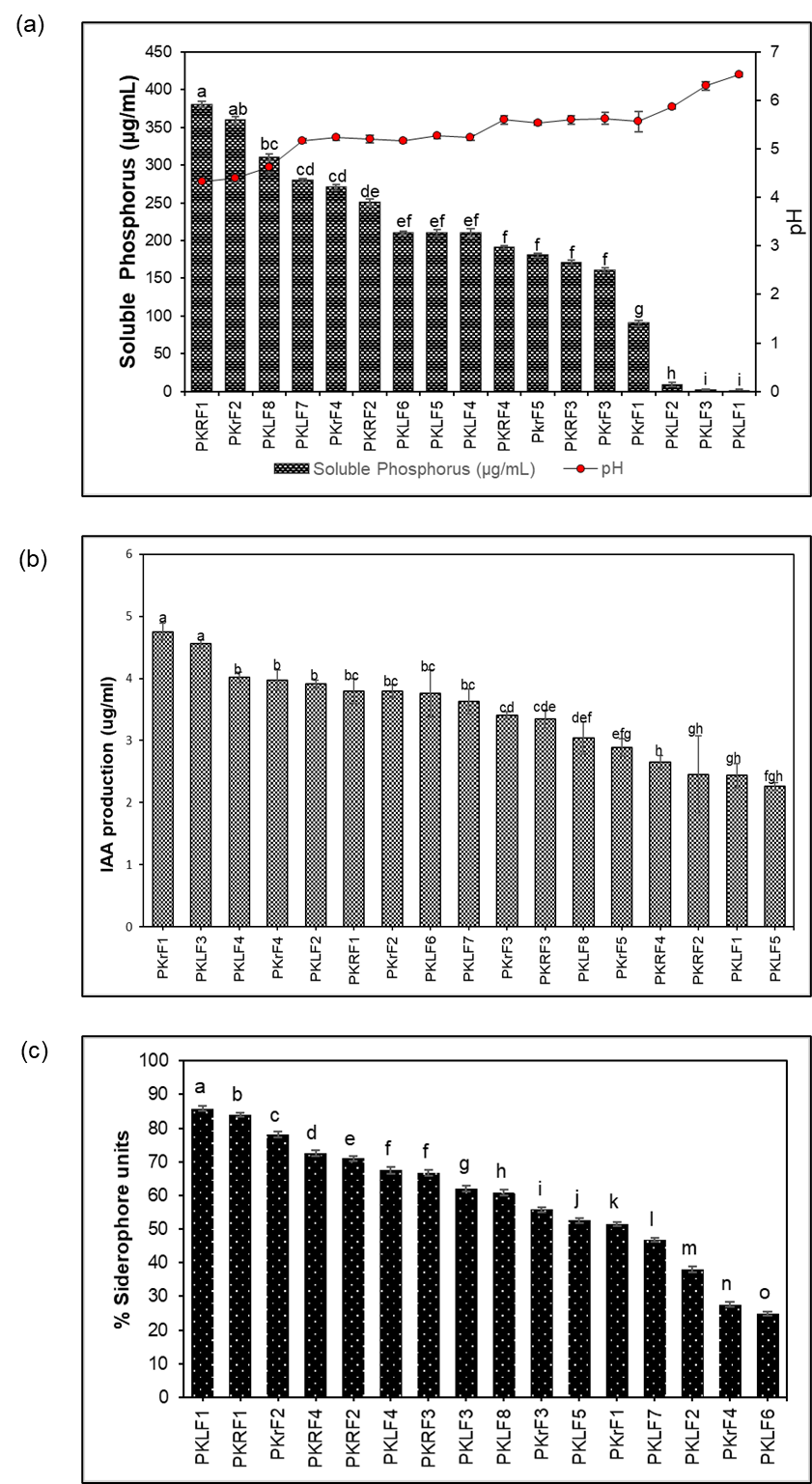
**

**Fig. S6** Plant growth-promoting traits of isolated fungal endophytes from P. kurrooa: (a) phosphate solubilization, where soluble phosphorus (μg/mL) in the culture supernatants was quantified and pH of supernatants was measured; (b) indole-3-acetic acid (IAA) production by endophytes quantified using Salkowski reagent; and (c) siderophore production by endophytes estimated using liquid Chrome Azurol S (CAS) assay. Data are presented as mean ± standard deviation (SD) of three independent replicates (n = 3). Bars with different letters indicate significant differences at p < 0.05, as determined using Duncan’s multiple range test.

**Supplementary Methods**

**Microscopic visualization of PKRF1 mycelia and spores**

Fungal mycelia and spores were visualized using light microscopy. A small portion of actively growing mycelium (5–7 days old, PDA plates) was gently scraped using a sterile scalpel and mounted on a glass slide in sterile water. One drop of lactophenol cotton blue (LPCB) stain was added, and the mycelial mat was carefully teased apart before covering with a coverslip. Samples were examined under a compound microscope at 10×, 40×, and 100× magnification, and representative images of hyphal structures and spore morphology were captured (Fig. S7 b & c) (Thomas et al. 1991).

**Plate-based assay for phosphate solubilization**

Qualitative assessment of phosphate solubilization was performed using Pikovskaya’s agar medium supplemented with tri-calcium phosphate (TCP, pH 7.2). Fungal disc (5 mm diameter) of PKRF1 was spot-inoculated at the center of agar plates and incubated at 28 °C for 7 days. The development of a clear halo around the colonies indicated phosphate solubilization (Fig. S7d) (Jian et al., 2020).

​ **Plate-based assay for siderophore production**

Siderophore production was qualitatively assayed on Chrome Azurol S (CAS) agar medium (Schwyn and Neilands, 1987). Fungal discs (5 mm diameter) of PKRF1 were inoculated at the center of CAS plates and incubated at 28 °C in the dark for 5–7 days. A distinct orange to yellow halo surrounding the colony was taken as evidence of siderophore production, reflecting decolorization of the CAS dye due to iron chelation (Fig. S7 c).

**References**

Schwyn B, Neilands JB. Universal chemical assay for the detection and determination of siderophores. Anal Biochem. 1987;160:47–56. https://doi: 10.1016/0003-2697(87)90612-9

Jain R, Bhardwaj P, Pandey SS, Kumar S. Arnebia euchroma, a plant species of cold desert in the Himalayas, harbors beneficial cultivable endophytes in roots and leaves. Front Microbiol. 2021;12:696667. https://doi: 10.3389/fmicb.2021.696667

Thomas PA, Kuriakose T, Kirupashanker MP, Maharajan VS. Use of lactophenol cotton blue mounts of corneal scrapings as an aid to the diagnosis of mycotic keratitis. Diagn Microbiol Infect Dis. 1991;14(3):219–224. https://doi: 10.1016/0732-8893(91)90036-2


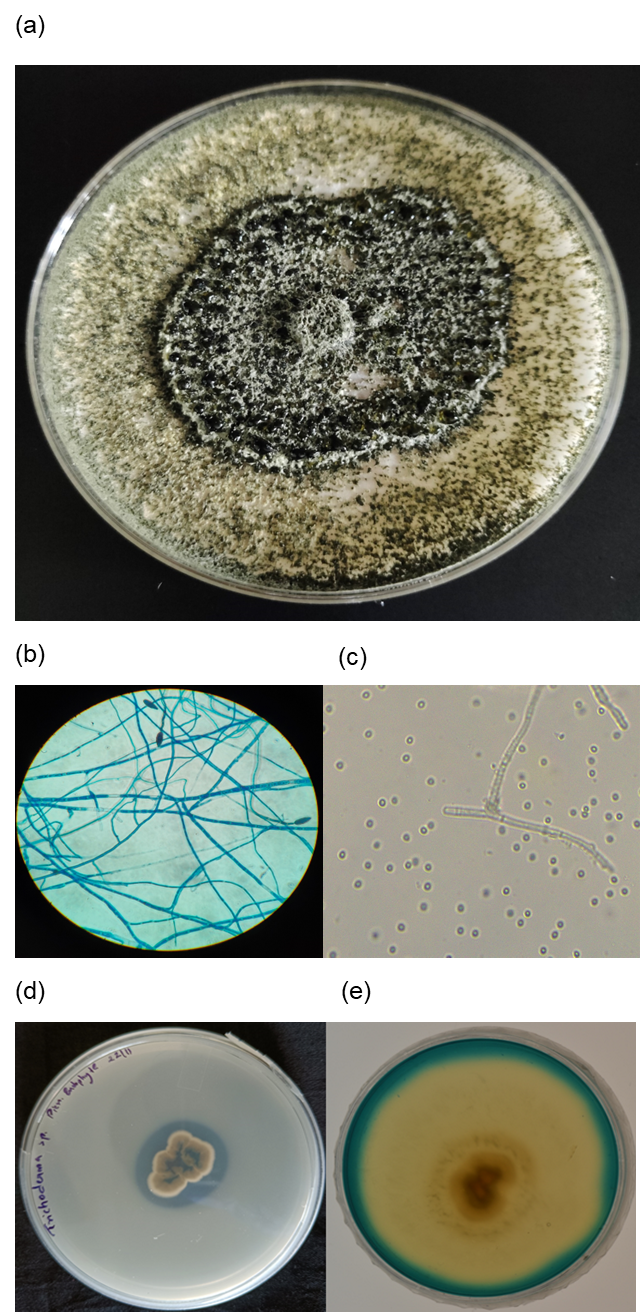


**Fig. S7** PKRF1 strain isolated from the rhizome of wild plants grown on potato dextrose agar (PDA); PKRF1 showing (b) mycelia and (c) spores. Qualitative plant growth-promoting (PGP) assays of PKRF1 strain showing (d) phosphate solubilization on Pikovskaya’s agar and (e) siderophore production on Chrome Azurol S (CAS) agar.

**Table S1 Relative abundance of different taxa at the phylum level present at various anatomical parts of Wt and Tc plants**

|  | **Plant tissues** | | | | | **Whole plant** | |
| --- | --- | --- | --- | --- | --- | --- | --- |
|  | TC rhizome | TC root | Wild leaf | Wild rhizome | Wild root | TC plant | Wild plant |
| Ascomycota | 99.12 | 84.77 | 100 | 77.55 | 58.51 | 94.94 | 71.64 |
| Basidiomycota | 0.88 | 11.68 | 0 | 16.45 | 41.49 | 4.03 | 24.25 |
| Fungi_phy_Incertae_sedis | 0 | 3.55 | 0 | 6.01 | 0 | 1.04 | 4.12 |

**Table S2: Relative abundance of different taxa at the genus level present at various anatomical parts of Wt and Tc plants**

|  | **Plant tissues** | | | | | **Whole plant** | |
| --- | --- | --- | --- | --- | --- | --- | --- |
| Taxonomy | TC rhizome | TC root | Wild leaf | Wild rhizome | Wild root | TC plant | Wild plant |
| *Acremonium* | 1.11 | 0 | 0 | 0.08 | 0 | 0.79 | 0.05 |
| *Agaricomycetes_gen_Incertae_sedis* | 0.88 | 10.31 | 0 | 12.05 | 29.02 | 3.63 | 17.33 |
| *Albifimbria* | 0 | 4.21 | 0 | 10.32 | 0 | 1.23 | 7.08 |
| *Arnium* | 0 | 0 | 0 | 0.72 | 0 | 0.00 | 0.49 |
| *Ascomycota_gen_Incertae_sedis* | 0 | 3.93 | 0 | 10.74 | 10 | 1.15 | 10.49 |
| *Branch06_gen_Incertae_sedis* | 0 | 0 | 0 | 0.76 | 1.32 | 0.00 | 0.93 |
| *Brunneochlamydosporium* | 0 | 0 | 0 | 0.75 | 0 | 0.00 | 0.51 |
| *Ceratobasidiaceae_gen_Incertae_sedis* | 0 | 1.37 | 0 | 2.66 | 4.45 | 0.40 | 3.21 |
| *Cyphellophora* | 0 | 0 | 0 | 2.35 | 0 | 0.00 | 1.61 |
| *Diaporthaceae_gen_Incertae_sedis* | 0.78 | 0 | 0 | 0 | 0 | 0.55 | 0.00 |
| *Diaporthe* | 11.94 | 0 | 0 | 0 | 0 | 8.46 | 0.00 |
| *Eurotiomycetes_gen_Incertae_sedis* | 0 | 0.76 | 0 | 0.45 | 6.5 | 0.22 | 2.34 |
| *Exophiala* | 0.47 | 0 | 0 | 0 | 0 | 0.33 | 0.00 |
| *Fungi_gen_Incertae_sedis* | 0 | 3.55 | 0 | 6.01 | 0 | 1.04 | 4.12 |
| *Fusarium* | 2.05 | 5.06 | 0 | 0.5 | 0.27 | 2.93 | 0.43 |
| *Hemimycena* | 0 | 0 | 0 | 0 | 8.01 | 0.00 | 2.51 |
| *Hymenoscyphus* | 0 | 0 | 0 | 0 | 0.77 | 0.00 | 0.24 |
| *Hypocreales_gen_Incertae_sedis* | 0 | 2.13 | 0 | 5.24 | 10.13 | 0.62 | 6.76 |
| *Ilyonectria* | 0 | 0.71 | 0 | 5.18 | 4.68 | 0.21 | 5.01 |
| *Leptodophora* | 0 | 0 | 0 | 1.67 | 1.52 | 0.00 | 1.62 |
| *Nigrograna* | 0.47 | 0 | 0 | 0 | 0 | 0.33 | 0.00 |
| *Not_Assigned* | 56 | 57.81 | 100 | 19.81 | 11.72 | 56.53 | 17.43 |
| *Paraleptospora* | 9.88 | 0 | 0 | 0 | 0 | 7.00 | 0.00 |
| *Paraphoma* | 6.06 | 5.87 | 0 | 10.99 | 0 | 6.00 | 7.53 |
| *Pleosporales_gen_Incertae_sedis* | 0 | 1.28 | 0 | 0.82 | 0 | 0.37 | 0.56 |
| *Polyscytalum* | 0 | 1.09 | 0 | 2.37 | 0 | 0.32 | 1.63 |
| *Populomyces* | 0 | 0 | 0 | 0.23 | 0 | 0.00 | 0.16 |
| *Preussia* | 0 | 0 | 0 | 0 | 0.47 | 0.00 | 0.15 |
| *Sordariales_gen_Incertae_sedis* | 10.37 | 0 | 0 | 0 | 9.91 | 7.34 | 3.10 |
| *Trichoderma* | 0 | 0 | 0 | 3.11 | 1.22 | 0.00 | 2.51 |
| *Verticillium* | 0 | 1.94 | 0 | 3.19 | 0 | 0.57 | 2.19 |

**Table S3 LefSe analysis statistical data for each taxon by sample groups for Figure S2**

| **Taxa** | **P values** | **FDR** | **TC rhizome** | **TC root** | **Wild leaf** | **Wild rhizome** | **Wild root** | **LDA score** |
| --- | --- | --- | --- | --- | --- | --- | --- | --- |
| Not_Assigned | 0.015115 | 0.038008 | 537600 | 548140 | 1.00E+06 | 209430 | 225450 | 5.6 |
| *Agaricomycetes_gen_Incertae_sedis* | 0.012923 | 0.038008 | 8275.8 | 103230 | 0 | 122880 | 215560 | 5.03 |
| *Ceratobasidiaceae_gen_Incertae_sedis* | 0.013448 | 0.038008 | 0 | 13858 | 0 | 28196 | 205700 | 5.01 |
| *Paraphoma* | 0.03628 | 0.05442 | 58176 | 62386 | 0 | 114690 | 0 | 4.76 |
| *Albifimbria* | 0.021204 | 0.039757 | 0 | 45432 | 0 | 106940 | 0 | 4.73 |
| *Ascomycota_gen_Incertae_sedis* | 0.026564 | 0.044273 | 0 | 34761 | 0 | 105230 | 73105 | 4.72 |
| *Sordariales_gen_Incertae_sedis* | 0.01647 | 0.038008 | 99568 | 0 | 0 | 0 | 23442 | 4.7 |
| *Paraleptospora* | 0.007977 | 0.038008 | 95163 | 0 | 0 | 0 | 0 | 4.68 |
| *Myrmecridium* | 0.007977 | 0.038008 | 93881 | 0 | 0 | 0 | 0 | 4.67 |
| *Hypocreales_gen_Incertae_sedis* | 0.057137 | 0.072751 | 0 | 18773 | 0 | 47732 | 74930 | 4.57 |
| *Fungi_gen_Incertae_sedis* | 0.01179 | 0.038008 | 0 | 66469 | 0 | 63021 | 0 | 4.52 |
| *Diaporthe* | 0.007977 | 0.038008 | 60324 | 0 | 0 | 0 | 0 | 4.48 |
| *Leptodophora* | 0.007977 | 0.038008 | 0 | 0 | 0 | 0 | 57891 | 4.46 |
| *Ilyonectria* | 0.01142 | 0.038008 | 0 | 8333.3 | 0 | 55154 | 35681 | 4.44 |
| *Fusarium* | 0.016262 | 0.038008 | 19638 | 51122 | 0 | 4653 | 1659.9 | 4.41 |
| *Eurotiomycetes_gen_Incertae_sedis* | 0.033746 | 0.053283 | 0 | 6768.2 | 0 | 3945.9 | 47677 | 4.38 |
| *Trichoderma* | 0.008153 | 0.038008 | 0 | 0 | 0 | 33406 | 9536.5 | 4.22 |
| *Verticillium* | 0.021204 | 0.039757 | 0 | 17244 | 0 | 33526 | 0 | 4.22 |
| *Polyscytalum* | 0.038745 | 0.055245 | 0 | 9729.3 | 0 | 25703 | 0 | 4.11 |
| *Leptodophora* | 0.026178 | 0.044273 | 0 | 0 | 0 | 17710 | 10185 | 3.95 |
| *Pleosporales_gen_Incertae_sedis* | 0.040513 | 0.055245 | 0 | 13755 | 0 | 8543.9 | 0 | 3.84 |
| *Acremonium* | 0.018433 | 0.039498 | 10693 | 0 | 0 | 690.54 | 0 | 3.73 |
| *Branch06_gen_Incertae_sedis* | 0.008153 | 0.038008 | 0 | 0 | 0 | 8023.4 | 9898.6 | 3.69 |
| *Brunneochlamydosporium* | 0.072751 | 0.072751 | 0 | 0 | 0 | 8414.7 | 0 | 3.62 |
| *Diaporthaceae_gen_Incertae_sedis* | 0.072751 | 0.072751 | 7618.9 | 0 | 0 | 0 | 0 | 3.58 |
| *Hymenoscyphus* | 0.072751 | 0.072751 | 0 | 0 | 0 | 0 | 6182.8 | 3.49 |

**Table S4** **Identification of fungal endophytes isolated from *P. kurrooa***

| **Plant Parts** | **Strain** | **Organism** | **Phylum** | **Class** | **NCBI accession number** |
| --- | --- | --- | --- | --- | --- |
| Rhizome | PKRF1 | *Trichoerma sp.* | Ascomycota | Sordariomycetes | PV528759 |
|  | PKRF2 | *Fusarium acuminatum* | Ascomycota | Sordariomycetes | PV528760 |
|  | PKRF3 | *Fusarium sp.* | Ascomycota | Sordariomycetes | PV528761 |
|  | PKRF4 | *Cladosporium tenuissimum* | Ascomycota | Dothideomycetes | PV528762 |
| Root | PKrF1 | *Fusarium tricinctum* | Ascomycota | Sordariomycetes | PV533722 |
|  | PKrF2 | *Pezicula ericae* | Ascomycota | Leotiomycetes | PV533723 |
|  | PKrF3 | *Pezicula ericae* | Ascomycota | Leotiomycetes | PV533724 |
|  | PKrF4 | *Fusarium sp.* | Ascomycota | Sordariomycetes | PV533725 |
|  | PKrF5 | *Alternaria sp* | Ascomycota | Dothideomycetes | PV533726 |
| Leaf | PKLF1 | *Alternaria alternata* | Ascomycota | Dothideomycetes | PV533727 |
|  | PKLF2 | *Ramularia cynarae* | Ascomycota | Dothideomycetes | PV533728 |
|  | PKLF3 | *Leptosphaeria sp* | Ascomycota | Dothideomycetes | PV533729 |
|  | PKLF4 | *Septoria carvi* | Ascomycota | Dothideomycetes | PV533730 |
|  | PKLF5 | *Septoria sp* | Ascomycota | Dothideomycetes | PV533731 |
|  | PKLF6 | *Bjerkandera adusta* | Basidiomycota | Agaricomycetes | PV533732 |
|  | PKFL7 | *Septoria carvi* | Ascomycota | Dothideomycetes | PV533763 |
|  | PKLF8 | *Septoria sp.* | Ascomycota | Dothideomycetes | PV533733 |

**Table S5** Quantitative estimation of *in-vitro* plant growth-promoting traits of the PKRF1 strain. Data are presented as mean ± standard deviation (SD) of three independent replicates.

| S/L No. | Isolates | IAA production (ug/ml) | Soluble Phosphorus (μg/mL) | % Siderophore units |
| --- | --- | --- | --- | --- |
| 1 | PKRF1 | 3.80±0.20 | 380.6±4.2 | 83.96±0.68 |
| 2 | PKRF2 | 2.46±0.62 | 250.5±4.6 | 70.99±0.78 |
| 3 | PKRF3 | 3.34±0.14 | 170.6±3.0 | 66.78±0.85 |
| 4 | PKRF4 | 2.65±0.11 | 190.8±2.5 | 67.45±0.98 |
| 5 | PKrF1 | 4.75±0.13 | 90.4±3.9 | 51.52±0.68 |
| 6 | PKrF2 | 3.80±0.11 | 360.3±4.4 | 78.22±0.85 |
| 7 | PKrF3 | 3.41±0.05 | 160.9±3.4 | 55.73±0.68 |
| 8 | PKrF4 | 3.97±0.16 | 270.5±3.7 | 27.59±0.81 |
| 9 | PKrF5 | 2.89±0.14 | 180.7±2.6 | ND |
| 10 | PKLF1 | 2.44±0.19 | 1.1±2.0 | 85.78±0.68 |
| 11 | PKLF2 | 3.91±0.06 | 8.9±3.9 | 38.01±0.85 |
| 12 | PKLF3 | 4.56±0.06 | 2.2±1.3 | 62.06±0.95 |
| 13 | PKLF4 | 4.02±0.08 | 210.1±5.2 | 67.45±0.98 |
| 14 | PKLF5 | 2.26±0.06 | 210.4±4.2 | 52.59±0.72 |
| 15 | PKLF6 | 3.76±0.38 | 210.4±4.6 | 24.76±0.62 |
| 16 | PKLF7 | 3.63±0.20 | 280.4±2.4 | 46.82±0.53 |
| 17 | PKLF8 | 3.04±0.26 | 310.4±4.0 | 60.84±0.82 |
|  |  |  |  | ND: not detected |

**Table S6** UHPLC estimation of secondary metabolites in PKRF1-treated and mock plants. The values are expressed as µg/mg dry weight (DW) of the respective tissues and are presented as mean ± standard deviation (SD) of five independent biological replicates , each consisting of pooled samples from eight plants per treatment. ND indicates non-detected

|  | **Control** | | **PKRF1** | |
| --- | --- | --- | --- | --- |
|  | **Leaf** | **Root** | **Leaf** | **Root** |
| PI | 5.028±1.87 | 0.0894±0.008 | 31.906±4.08 | 0.0842±0.019 |
| PII | 0.344±0.14 | 2.541±0.08 | 3.601±0.12 | 5.377±0.142 |
| Cinnamic acid | 0.398±0.019 | ND | 0.7444±0.0015 | ND |
| Caffeic acid | 0.379±0.004 | 0.0533±0.004 | 0.807±0.036 | 0.237±0.01 |
| Acubin | 1.166±0.369 | 0.422±0.305 | 6.736±0.668 | 3.044±0.461 |
| Catalpol | 2.217±0.57 | 16.574±0.687 | 7.101±0.46 | 16.66±0.144 |

**Table S7 Unique plant-associated gene present in PKRF1 in comparison to other closely related *Trichoderma* genomes**

| level1 | level2 | level3 | level4 | level5 | level6 | freq |
| --- | --- | --- | --- | --- | --- | --- |
| Indirect effects | Colonizing plant system | Cell wall/membrane degradation | Plant degradative glycosidases/glycosylhydrolases | Plant degradative gs/gh-glucosidase | mall->pgpt0019145 | 3 |
| Indirect effects | Colonizing plant system | Plant-derived substrate usage | Plant-derived glycoside utilization | Plant-derived glycoside-glycosidases/glycosylhydrolases | mall->pgpt0019145 | 3 |
| Indirect effects | Competitive exclusion | Bacterial fitness | Bacterial fitness-multidrug resistance | Multidrug efflux genes | efrb->pgpt0029030 | 1 |
| Indirect effects | Competitive exclusion | Bacterial fitness | Bacterial fitness-multidrug resistance | Multidrug efflux genes | mdlb/smdb->pgpt0029045 | 1 |
| Indirect effects | Competitive exclusion | Exopolysaccharide production(eps) | EPS-glycosidases/glycosylhydrolases | EPS-glucosidase | mall->pgpt0019145 | 3 |
| Indirect effects | Stress control | Neutralizing abiotic stress | Nitrosative/oxidative stress/ROS scavenging | Detoxification of peroxidized compounds | gst->pgpt0013170 | 1 |
| Indirect effects | Stress control | Neutralizing biotic stress | Bactericidal compounds\| antibiotics | Bacitracin A metabolism | bacc->pgpt0010190 | 1 |
| Indirect effects | Stress control | Neutralizing biotic stress | Bactericidal compounds\| antibiotics | Tyrocidin A metabolism | tycc->pgpt0010650 | 3 |
| Indirect effects | Stress control | Neutralizing biotic stress | Fungicidal compounds\| antibiotics | Fengycin/plipastatin metabolism | ppsB/fenD->PGPT0012305 | 1 |

| Record | Total count | NRPS | NRPS-like | T1PKS | fungal-RiPP-like | hybrid | isocyanide-nrp | terpene |
| --- | --- | --- | --- | --- | --- | --- | --- | --- |
| *Trichoderma*_*harzianum*_Tr1 | 48 | 5 | 2 | 15 | 4 | 16 | 1 | 5 |
| *Trichoderma*_*harzianum*_CBS_354.33 | 50 | 6 | 2 | 16 | 5 | 13 | 1 | 7 |
| *Trichoderma*_*harzianum*_T6776 | 50 | 7 | 4 | 18 | 6 | 8 | 1 | 6 |
| *Trichoderma*_sp._PKRF1 | 50 | 3 | 5 | 17 | 6 | 11 | 1 | 7 |
| *Trichoderma*_*harzianum*_TR274 | 51 | 5 | 7 | 19 | 7 | 7 | 1 | 5 |

**Table S9 Comparative Biosynthetic gene clusters predicted through AntiSMASH analysis for PKRF1 and other related genomes**

**Table S10 Detailed anti-smash predicted biosynthetic gene clusters in PKRF1**

| Start | End | Product | Known Biosynthetic gene Cluster (KBC) hit | KBC acc | KBC % |
| --- | --- | --- | --- | --- | --- |
| 503594 | 564355 | fungal-RiPP-like | - | - | - |
| 0 | 46359 | fungal-RiPP-like | - | - | - |
| 129134 | 190279 | fungal-RiPP-like | - | - | - |
| 136987 | 197433 | fungal-RiPP-like | - | - | - |
| 40330 | 101363 | fungal-RiPP-like | - | - | - |
| 50988 | 113869 | fungal-RiPP-like | - | - | - |
| 516966 | 608264 | fungal-RiPP-like / NRPS-like | - | - | - |
| 286251 | 365231 | fungal-RiPP-like / NRPS-like | choline | BGC0002276 | 100 |
| 52969 | 107877 | isocyanide-nrp | - | - | - |
| 844399 | 887822 | NRPS | - | - | - |
| 294172 | 339693 | NRPS | metachelin C/metachelin A/metachelin A-CE/metachelin B/dimerumic acid 11-mannoside/dimerumic acid | BGC0002710 | 62 |
| 0 | 39868 | NRPS | - | - | - |
| 0 | 92845 | NRPS / T1PKS | - | - | - |
| 0 | 87886 | NRPS / T1PKS | dichlorodiaporthin | BGC0002237 | 50 |
| 110994 | 168557 | NRPS / T1PKS | - | - | - |
| 47280 | 99949 | NRPS / T1PKS | - | - | - |
| 52731 | 104008 | NRPS / T1PKS / betalactone | harzianopyridone | BGC0002066 | 50 |
| 100523 | 143645 | NRPS-like | - | - | - |
| 403988 | 447057 | NRPS-like | - | - | - |
| 268561 | 311640 | NRPS-like |  | - | - |
| 109276 | 148370 | NRPS-like | - | - | - |
| 0 | 40450 | NRPS-like | - | - | - |
| 380878 | 429847 | NRPS-like / NRPS | - | - | - |
| 0 | 77477 | NRPS-like / NRPS | - | - | - |
| 505851 | 554073 | T1PKS | clavaric acid | BGC0001248 | 100 |
| 532059 | 579583 | T1PKS | - | - | - |
| 267049 | 325099 | T1PKS | tricholignan A | BGC0001854 | 88 |
| 118120 | 168176 | T1PKS | - | - | - |
| 219336 | 291117 | T1PKS | harziphilone/t22azaphilone/isoharziphilone-1/isoharziphilone-2/compound 4/compound 1 | BGC0002206 | 100 |
| 79561 | 125023 | T1PKS | aurofusarin | BGC0002709 | 18 |
| 133999 | 182573 | T1PKS | - | - | - |
| 0 | 32518 | T1PKS | decumbenone a/calbistrin A/calbistrin C/decumbenone B/decumbenone c/dioic acid moiety | BGC0002168 | 15 |
| 137903 | 185356 | T1PKS | - | - | - |
| 142122 | 190048 | T1PKS | tryptoquialanine | BGC0001142 | 9 |
| 127031 | 174197 | T1PKS | depudecin | BGC0000046 | 33 |
| 13575 | 61474 | T1PKS | - | - | - |
| 0 | 45430 | T1PKS | - | - | - |
| 0 | 36331 | T1PKS | trichoxide | BGC0002233 | 25 |
| 50731 | 95883 | T1PKS | - | - | - |
| 54550 | 100229 | T1PKS | - | - | - |
| 11957 | 53769 | T1PKS | - | - | - |
| 42675 | 126258 | T1PKS / NRPS | - | - | - |
| 44636 | 96930 | T1PKS / NRPS | - | - | - |
| 409360 | 430535 | terpene | - | - | - |
| 417534 | 438803 | terpene | - | - | - |
| 403913 | 424575 | terpene | - | - | - |
| 48066 | 69115 | terpene | - | - | - |
| 64453 | 86064 | terpene | trichobrasilenol/xylarenic acid B/brasilane A/brasilane F/brasilane E/brasilane D | BGC0002260 | 60 |
| 122965 | 137803 | terpene | squalestatin S1 | BGC0001839 | 40 |
| 39859 | 61209 | terpene | - | - | - |

**Table S11:** Detail of primer used in Real-time PCR analysis

| **Gene Name** | **Sequence 5´-3´** |
| --- | --- |
| *26S* | F-CACAATGATAGGAAGAGCCGAC  R-CAAGGGAACGGGCTTGGCAGAATC |
| *DXS* | F-ACATTTAAGTTCAAGTCTGGGAGTG  R-ATGTGCACTCTCTTCTCTTTTAGGA |
| *DXR* | F-GGAGGAACTATGACTGGTGTTCTT  R-CAGGTCATAGTGTACGATTTCCTCT |
| *G10H* | F-TATCGAGCTTTTCAGTGGAT  R-GATGTGAGTCCTGTCGATTT |
| *CAM* | F-GAAGATGCTCCTTCTTATCC  R-AACACTCGACCAGAATCAC |
| *C4H* | F-GCAACATTGATGTTCTCAAC  R-TCCAGCTCTTCAAGGACTAT |
| *DAHPS* | F-ACACCATTAAAGCTCCTTGT  R-TAACAGTCTGAGATCCACCA |
| *PAL* | F-GCAAGATAGATACGCTCTAA  R-GTTCCTTGAGACGTCAAT |
